# Performance of a Machine Learning Algorithm Using Electronic Health Record Data to Predict Postoperative Complications and Report on a Mobile Platform

**DOI:** 10.1001/jamanetworkopen.2022.11973

**Published:** 2022-05-16

**Authors:** Yuanfang Ren, Tyler J. Loftus, Shounak Datta, Matthew M. Ruppert, Ziyuan Guan, Shunshun Miao, Benjamin Shickel, Zheng Feng, Chris Giordano, Gilbert R. Upchurch, Parisa Rashidi, Tezcan Ozrazgat-Baslanti, Azra Bihorac

**Affiliations:** 1 Intelligent Critical Care Center, University of Florida, Gainesville; 2 Division of Nephrology, Hypertension, and Renal Transplantation, Department of Medicine, University of Florida, Gainesville; 3 Department of Surgery, University of Florida, Gainesville; 4 Department of Electrical and Computer Engineering, University of Florida, Gainesville; 5 Department of Anesthesiology, University of Florida, Gainesville; 6 Department of Biomedical Engineering, University of Florida, Gainesville

## Abstract

**Importance:**

Predicting postoperative complications has the potential to inform shared decisions regarding the appropriateness of surgical procedures, targeted risk-reduction strategies, and postoperative resource use. Realizing these advantages requires that accurate real-time predictions be integrated with clinical and digital workflows; artificial intelligence predictive analytic platforms using automated electronic health record (EHR) data inputs offer an intriguing possibility for achieving this, but there is a lack of high-level evidence from prospective studies supporting their use.

**Objective:**

To examine whether the MySurgeryRisk artificial intelligence system has stable predictive performance between development and prospective validation phases and whether it is feasible to provide automated outputs directly to surgeons’ mobile devices.

**Design, Setting, and Participants:**

In this prognostic study, the platform used automated EHR data inputs and machine learning algorithms to predict postoperative complications and provide predictions to surgeons, previously through a web portal and currently through a mobile device application. All patients 18 years or older who were admitted for any type of inpatient surgical procedure (74 417 total procedures involving 58 236 patients) between June 1, 2014, and September 20, 2020, were included. Models were developed using retrospective data from 52 117 inpatient surgical procedures performed between June 1, 2014, and November 27, 2018. Validation was performed using data from 22 300 inpatient surgical procedures collected prospectively from November 28, 2018, to September 20, 2020.

**Main Outcomes and Measures:**

Algorithms for generalized additive models and random forest models were developed and validated using real-time EHR data. Model predictive performance was evaluated primarily using area under the receiver operating characteristic curve (AUROC) values.

**Results:**

Among 58 236 total adult patients who received 74 417 major inpatient surgical procedures, the mean (SD) age was 57 (17) years; 29 226 patients (50.2%) were male. Results reported in this article focus primarily on the validation cohort. The validation cohort included 22 300 inpatient surgical procedures involving 19 132 patients (mean [SD] age, 58 [17] years; 9672 [50.6%] male). A total of 2765 patients (14.5%) were Black or African American, 14 777 (77.2%) were White, 1235 (6.5%) were of other races (including American Indian or Alaska Native, Asian, Native Hawaiian or Pacific Islander, and multiracial), and 355 (1.9%) were of unknown race because of missing data; 979 patients (5.1%) were Hispanic, 17 663 (92.3%) were non-Hispanic, and 490 (2.6%) were of unknown ethnicity because of missing data. A greater number of input features was associated with stable or improved model performance. For example, the random forest model trained with 135 input features had the highest AUROC values for predicting acute kidney injury (0.82; 95% CI, 0.82-0.83); cardiovascular complications (0.81; 95% CI, 0.81-0.82); neurological complications, including delirium (0.87; 95% CI, 0.87-0.88); prolonged intensive care unit stay (0.89; 95% CI, 0.88-0.89); prolonged mechanical ventilation (0.91; 95% CI, 0.90-0.91); sepsis (0.86; 95% CI, 0.85-0.87); venous thromboembolism (0.82; 95% CI, 0.81-0.83); wound complications (0.78; 95% CI, 0.78-0.79); 30-day mortality (0.84; 95% CI, 0.82-0.86); and 90-day mortality (0.84; 95% CI, 0.82-0.85), with accuracy similar to surgeons’ predictions. Compared with the original web portal, the mobile device application allowed efficient fingerprint login access and loaded data approximately 10 times faster. The application output displayed patient information, risk of postoperative complications, top 3 risk factors for each complication, and patterns of complications for individual surgeons compared with their colleagues.

**Conclusions and Relevance:**

In this study, automated real-time predictions of postoperative complications with mobile device outputs had good performance in clinical settings with prospective validation, matching surgeons’ predictive accuracy.

## Key Points

**Question:**

Is an artificial intelligence platform able to accurately predict postoperative complications using automated real-time electronic health record data and mobile device outputs?

**Findings:**

In this prognostic study of 74 417 inpatient surgical procedures involving 58 236 adult patients, random forest models using 135 features had the greatest overall discrimination and the best performance during prospective validation, matching surgeons’ predictive accuracy. Model outputs, including the top 3 risk factors associated with each postoperative complication, were exported to mobile devices with high speed and fidelity.

**Meaning:**

This study’s findings suggest that accurate data-based predictions of postoperative complications that are integrated with clinical workflow have the potential to augment surgical decision-making.

## Teaser

This prognostic study examines whether an artificial intelligence platform is able to accurately predict postoperative complications using automated real-time electronic health record data and provide automated outputs directly to surgeons’ mobile devices.

## Introduction

In the US alone, more than 15 million inpatient surgical procedures are performed annually.^1,2^ Postoperative complications occur in as many as 32% of procedures, increasing costs by as much as $11 000 per major complication.^3,4^ Cognitive and judgment errors are major sources of potentially preventable complications.^4,5^ For example, underestimation of the risk of complications may be associated with postoperative undertriage of high-risk patients to general wards rather than intensive care units (ICUs) and an increased prevalence of hospital mortality.^6^

High-performance data-based clinical decision support has the potential to mitigate harm from cognitive errors occurring when estimating the risk of postoperative complications. All patients have a unique risk profile that is specific to their demographic characteristics, comorbid conditions, physiological reserve, planned surgical procedure, and surgeon’s skill; clinicians have had mediocre performance in estimating risk probabilities.^7^ Decision support tools are intended to augment these estimations, but many are hindered by time-consuming manual data entry requirements and lack of integration with clinical workflow.^8,9,10,11,12,13^ Artificial intelligence (AI) predictive analytic platforms using automated electronic health record (EHR) data inputs may be able to mitigate these challenges, but there is a lack of high-level evidence from prospective studies supporting their use.^14,15^

The purpose of this prognostic study was to describe the prospective validation of the MySurgeryRisk platform, which uses automated EHR data to make data-based patient-level predictions of postoperative complications and mortality. Using a large inpatient surgical cohort, we tested the hypotheses that the system would have stable performance between development and prospective validation phases and that it would be feasible to provide automated outputs directly to surgeons’ mobile devices.

## Methods

### Study Design

An intelligent perioperative platform was developed and deployed to integrate EHR data, AI algorithms, and clinician interactions on mobile devices for real-time surgical risk prediction. Using this platform, we combined retrospectively and prospectively collected perioperative data linked with public data sets to optimize and prospectively validate an algorithmic toolkit for predicting the risk of 8 major postoperative complications and death after inpatient surgical procedures.^16^ A flow diagram showing temporal associations between automated real-time data inputs and outcome prediction windows is available in the Figure. The University of Florida Institutional Review Board and Privacy Office approved this study as an exempt study^12^ with a waiver of informed consent because this research presented no more than the minimal risk of harm to participants and involved no procedures for which written consent was required outside of the research context. This study followed the Transparent Reporting of a Multivariable Prediction Model for Individual Prognosis or Diagnosis (TRIPOD) reporting guideline.^17^

**Figure.**
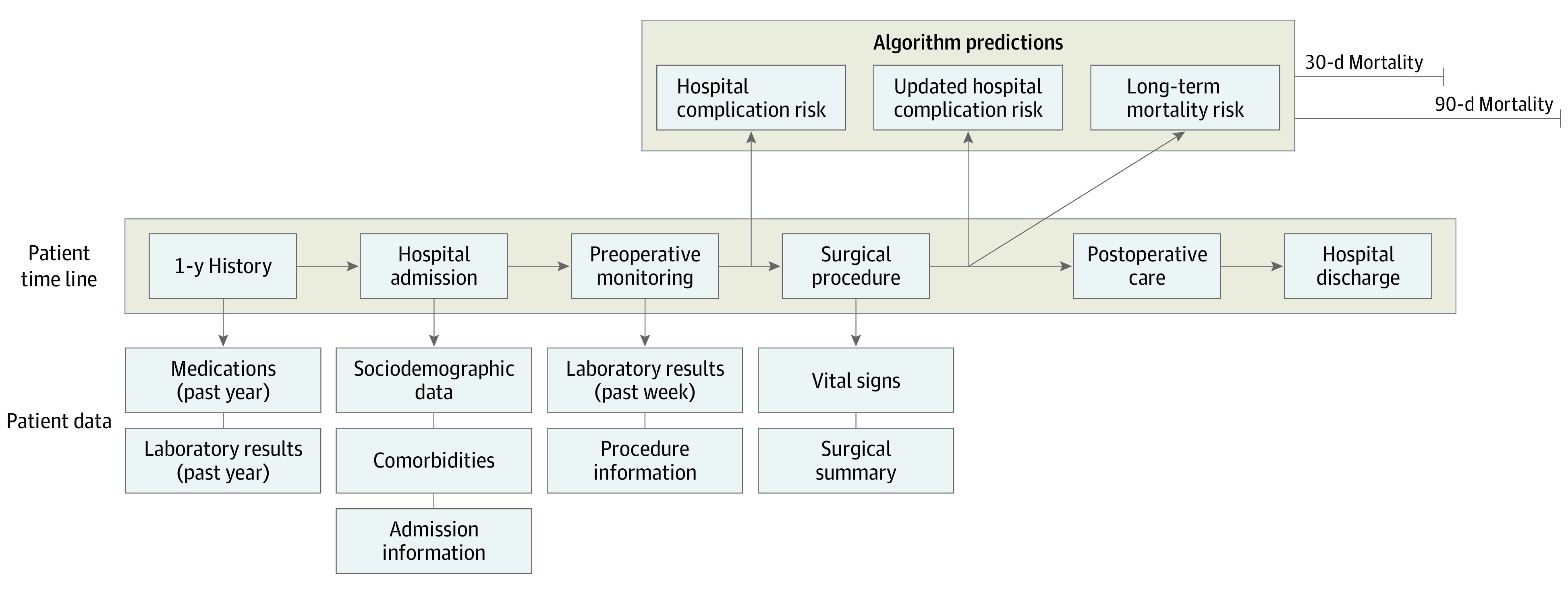
Temporal Associations Between Automated Real-Time Data Inputs and Outcome Prediction Windows Electronic health record data accrued 1 year before surgical procedures were used to predict the risk of postoperative complications occurring during admission as well as 30-day and 90-day mortality.

### Participants

We included all patients 18 years or older who were admitted to University of Florida Health Gainesville for any type of inpatient surgical procedure between June 1, 2014, and September 20, 2020. Minor procedures performed for the purpose of controlling pain, gastrointestinal-related minor procedures, and organ donation procedures were excluded. Detailed exclusion criteria used to identify encounters with completed inpatient surgical procedures are shown in eFigure 1 in the Supplement. When a patient received multiple surgical procedures during 1 admission, only the first procedure was used in the analysis. The total sample comprised 58 236 adult patients who received 74 417 inpatient surgical procedures. The final retrospective (development) cohort consisted of 41 812 patients who received 52 117 procedures between June 1, 2014, and November 27, 2018; the prospective (validation) cohort consisted of 19 132 patients who underwent 22 300 procedures between November 28, 2018, and September 20, 2020. Data were collected using a real-time intelligent perioperative platform.

### Data Integration and Harmonization

The University of Florida Integrated Data Repository functioned as an honest broker in deidentifying EHR data while preserving data set temporality and links between patient and surgeon identifiers. For both retrospective and prospective data sets, we developed extraction, transformation, and loading routines for converting native EHR formats to data standards, including the Observational Medical Outcomes Partnership common data model,^18^ RxNorm medication terminology from the National Library of Medicine,^19^ US Veterans Health Administration National Drug File reference terminology,^20^ and the Logical Observation Identifiers Names and Codes standards.^2^ For each patient’s medical record containing heterogeneous variables (eg, demographic characteristics and medical history, diagnoses and procedures, medications, laboratory results, and vital signs), we used several validated preprocessing algorithms for handling outliers, missing values, normalization, and resampling.^15,21,22,23,24,25,26^ We linked EHR data with US census data to ascertain social determinants of health and long-term mortality.^15,27^

### Algorithmic Toolkit

We developed and implemented several AI algorithms for perioperative real-time data integration, harmonization and preprocessing, computable phenotyping, and dynamic perioperative risk prediction for 8 postoperative complications, including prolonged (>48 hours) ICU stay; prolonged mechanical ventilation; neurological complications, including delirium; cardiovascular complications; acute kidney injury; venous thromboembolism; sepsis; and wound complications. We reported 6 model versions, including 3 generalized additive models and 3 random forest models using 55, 101, and 135 input features (eTable 1 in the Supplement). These input features included preoperative demographic, socioeconomic, administrative, clinical, pharmacy, and laboratory variables. These models followed the same methods for data preprocessing, feature selection, and model development as previously described^15^ and detailed in eMethods in the Supplement.

### Real-Time Intelligent Perioperative Platform

The MySurgeryRisk platform is an intelligent system for real-time processing of clinical data and deployment of analytic pipelines that push results to surgeons’ mobile devices (eFigure 2 in the Supplement). The platform provides a private cloud-based intelligent engine coupled with a standard data model (developed by the Observational Medical Outcomes Partnership^18^) and a standard data exchange protocol (Fast Healthcare Interoperability Resources^28^) to generate a unified real-time data analysis. Web and mobile applications provide a graphic visualization of surgical risk predictions to physicians (eFigures 3-11 in the Supplement).

### Sample

Algorithms were trained on data from the development cohort; most results reported in this article are from the validation cohort. Using the validation cohort (n = 22 300 surgical procedures) with the 1000-sample bootstrap method and assuming an area under the receiver operating characteristic curve (AUROC) of 0.80, the overall sample size allowed a maximum 95% CI width for the AUROC of 0.05 when the prevalence of a postoperative complication was 2% and 0.01 when the prevalence was 30%. Higher AUROCs would produce narrower 95% CIs.

### Statistical Analysis

We assessed each model’s discrimination using AUROC values. For each postoperative complication, low-risk vs high-risk groups were identified using cutoff values that yielded the highest Youden index (ie, the highest sum of sensitivity and specificity).^29^ These cutoff values were used to determine the fraction of correct classifications as well as sensitivity, specificity, positive predictive value, and negative predictive value for each model in the validation cohort. We used bootstrap sampling and nonparametric methods to obtain 95% CIs for all performance measures. Development cohort AUROC values were generated using 5-fold cross-validation. Data were analyzed using Python software, version 3.7 (Python Software Foundation). The threshold for statistical significance was 2-tailed *P* = .05.

## Results

### Participant Baseline Characteristics and Outcomes

Among 58 236 total adult patients who received 74 417 major inpatient surgical procedures, the mean (SD) age was 57 (17) years; 29 010 patients (49.8%) were female, and 29 226 (50.2%) were male (eTable 2 in the Supplement). The retrospective development cohort included 52 117 inpatient surgical procedures involving 41 812 patients (mean [SD] age, 56 [18] years; 20 982 [50.2%] female and 20 830 [49.8%] male).

The prospective validation cohort included 22 300 inpatient surgical procedures involving involving 19 132 patients (mean [SD] age, 58 [17] years; 9672 [50.6%] male). A total of 2765 patients (14.5%) were Black or African American, 14 777 (77.2%) were White, 1235 (6.5%) were of other races (including American Indian or Alaska Native, Asian, Native Hawaiian or Pacific Islander, and multiracial), and 355 (1.9%) were of unknown race because of missing data; 979 patients (5.1%) were Hispanic, 17 663 (92.3%) were non-Hispanic, and 490 (2.6%) were of unknown ethnicity because of missing data. All major procedure types (including cardiothoracic, gastrointestinal, neurological, obstetric, oncological, otolaryngological, urological, and vascular) were well represented. The prevalence of postoperative complications in the validation cohort was 28.5% for prolonged ICU stay; 5.6% for mechanical ventilation longer than 48 hours; 15.1% for neurological complications, including delirium; 15.7% for acute kidney injury; 16.4% for cardiovascular complications; 5.6% for venous thromboembolism; 21.6% for wound complications; 1.9% for 30-day mortality; and 3.0% for 90-day mortality.

There was slight variation in complication prevalence between the development and validation cohorts (eg, in the development cohort, the prevalence was 10.7% for neurological complications, including delirium; 23.3% for ICU stay longer than 48 hours; and 14.7% for wound complications). Additional details regarding patient demographic characteristics and complication prevalence in the development and validation cohorts are shown in Table 1 and eTable 2 in the Supplement.

**Table 1.** Patient Characteristics

Characteristic	No. (%)	
	Development cohort^a^	Validation cohort^b^
Total inpatient surgical procedures, No.	52 117	22 300
Age, mean (SD), y^c^	56 (18)	58 (17)
Sex		
Male	26 071 (50.0)	11 373 (51.0)
Female	26 046 (50.0)	10 927 (49.0)
Race^d^		
Black or African American	6225 (14.9)	2765 (14.5)
White	32 286 (77.2)	14 777 (77.2)
Other race^e^	2667 (6.4)	1235 (6.5)
Missing	634 (1.5)	355 (1.9)
Ethnicity^d^		
Hispanic	1987 (4.7)	979 (5.1)
Non-Hispanic	39 067 (93.4)	17 663 (92.3)
Missing	758 (1.8)	490 (2.6)
Marital status^c^		
Married	19 940 (47.7)	8986 (47.0)
Single	15 362 (36.7)	7303 (38.2)
Divorced	6190 (14.8)	2709 (14.2)
Missing	320 (0.8)	134 (0.7)
Insurance status^c^		
Medicare	18 451 (44.1)	9183 (47.0)
Private	13 255 (31.7)	5447 (28.5)
Medicaid	6727 (16.1)	2757 (14.4)
Uninsured	3379 (8.1)	1745 (9.1)
Complications^f^		
Acute kidney injury	6971 (13.4)	3506 (15.7)
Cardiovascular complications	6403 (12.3)	3659 (16.4)
Neurological complications, including delirium	5570 (10.7)	3376 (15.1)
Prolonged ICU stay	12 167 (23.3)	6363 (28.5)
Prolonged mechanical ventilation	2766 (5.3)	1247 (5.6)
Sepsis	3802 (7.3)	1966 (8.8)
Venous thromboembolism	2267 (4.3)	1256 (5.6)
Wound complications	7651 (14.7)	4827 (21.6)
30-d Mortality	1047 (2.0)	429 (1.9)
90-d Mortality	1893 (3.6)	663 (3.0)

Abbreviation: ICU, intensive care unit.

^a^ Includes 41 812 patients admitted between June 1, 2014, and November 27, 2018.

^b^ Includes 19 132 patients admitted between November 28, 2018, and September 20, 2020.

^c^ Data were reported based on values calculated at the latest hospital admission.

^d^ Race and ethnicity were self-reported.

^e^ Other races include American Indian or Alaska Native, Asian, Native Hawaiian or Pacific Islander, and multiracial.

^f^ Data were reported based on postoperative complication status for each surgical procedure.

### Generalized Additive Model Performance

Three generalized additive models were developed using 55, 101, and 135 input features. We evaluated model performance by calculating AUROC values (shown in Table 2), accuracy, sensitivity, specificity, positive predictive values, and negative predictive values (shown in eTable 3 in the Supplement). In the model with 135 features using data from the prospective validation cohort, AUROC values ranged from 0.77 for wound complications to 0.91 for prolonged mechanical ventilation.

**Table 2.** Automated Real-Time Predictions of Postoperative Complications and Outcomes by Number of Input Features in the Generalized Additive Model

Complication or outcome	AUROC (95% CI)^a^								
	55 features			101 features			135 features		
	Development cohort	Validation cohort	*P* value	Development cohort	Validation cohort	*P* value	Development cohort	Validation cohort	*P* value
Cardiovascular complications	0.82 (0.82-0.83)	0.80 (0.79-0.80)	<.001	0.82 (0.81-0.82)	0.78 (0.77-0.79)	<.001	0.83 (0.83-0.84)	0.81 (0.80-0.82)	<.001
Prolonged ICU stay	0.90 (0.90-0.90)	0.86 (0.86-0.87)	<.001	0.90 (0.90-0.90)	0.85 (0.84-0.86)	<.001	0.91 (0.91-0.92)	0.88 (0.87-0.88)	<.001
Neurological complications, including delirium	0.89 (0.88-0.89)	0.85 (0.85-0.86)	<.001	0.87 (0.86-0.87)	0.83 (0.82-0.84)	<.001	0.89 (0.89-0.90)	0.86 (0.86-0.87)	<.001
Wound complications	0.81 (0.81-0.82)	0.77 (0.76-0.77)	<.001	0.75 (0.74-0.76)	0.69 (0.68-0.70)	<.001	0.81 (0.80-0.81)	0.77 (0.77-0.78)	<.001
Sepsis	0.87 (0.86-0.88)	0.84 (0.83-0.84)	<.001	0.87 (0.86-0.87)	0.84 (0.83-0.85)	<.001	0.88 (0.88-0.89)	0.86 (0.85-0.86)	<.001
Venous thromboembolism	0.83 (0.83-0.84)	0.80 (0.79-0.81)	<.001	0.82 (0.81-0.83)	0.78 (0.77-0.79)	<.001	0.84 (0.83-0.85)	0.81 (0.80-0.83)	.001
Prolonged mechanical ventilation	0.91 (0.91-0.92)	0.89 (0.88-0.90)	<.001	0.90 (0.90-0.91)	0.87 (0.86-0.88)	<.001	0.92 (0.92-0.93)	0.91 (0.90-0.91)	<.001
Acute kidney injury	0.83 (0.82-0.83)	0.80 (0.79-0.80)	<.001	0.82 (0.82-0.83)	0.79 (0.78-0.79)	<.001	0.84 (0.84-0.85)	0.82 (0.81-0.83)	<.001
30-d Mortality	0.86 (0.84-0.87)	0.84 (0.82-0.86)	.07	0.86 (0.85-0.87)	0.82 (0.80-0.84)	.002	0.87 (0.86-0.88)	0.84 (0.82-0.86)	.007
90-d Mortality	0.84 (0.83-0.85)	0.82 (0.81-0.84)	.07	0.84 (0.83-0.85)	0.81 (0.80-0.83)	.003	0.85 (0.84-0.86)	0.82 (0.80-0.84)	.009

Abbreviations: AUROC, area under the receiver operating characteristic curve; ICU, intensive care unit.

^a^ AUROC values with 95% CIs were obtained from bootstrapping with 1000 samples. *P* values comparing AUROC values between the development vs validation cohorts were calculated using the DeLong unpaired method.

A greater number of input features was associated with stable or improved model performance. For example, the model using 135 features to predict acute kidney injury achieved an AUROC of 0.82 (95% CI, 0.81-0.83) in the validation cohort, which was significantly greater than the AUROC for the model using 55 features (0.80; 95% CI, 0.79-0.80). The model using 135 features to predict prolonged mechanical ventilation achieved an AUROC of 0.91 (95% CI, 0.90-0.91), which was significantly greater than the AUROC for the model using 55 features (0.89; 95% CI, 0.88-0.90). There were no postoperative complications for which 135 features yielded lower discrimination than 55 features.

We observed performance degradation in the prediction of several postoperative complications during prospective validation. In the model using 135 features, the AUROC values in the development cohort were greater than those of the validation cohort for all complications (eg, wound complications: 0.81 [95% CI, 0.80-0.81] vs 0.77 [95% CI, 0.77-0.78]; prolonged ICU stay: 0.91 [95% CI, 0.91-0.92] vs 0.88 [95% CI, 0.87-088]), with all AUROC improvements ranging from 0.01 for prolonged mechanical ventilation to 0.04 for wound complications. The relative contributions of each input feature for each model are shown in eTables 4 to 6 in the Supplement.

### Random Forest Model Performance

Three random forest models were developed using 55, 101, and 135 input features, matching the feature sets used for the generalized additive models. We evaluated model performance by calculating AUROC values (shown in Table 3), accuracy, sensitivity, specificity, positive predictive values, and negative predictive values (shown in eTable 7 in the Supplement). In the model with 135 features using data from the prospective validation cohort, AUROC values ranged from 0.78 to 0.91 (acute kidney injury: 0.82 [95% CI, 0.82-0.83]; cardiovascular complications: 0.81 [95% CI, 0.81-0.82]; neurological complications, including delirium: 0.87 [95% CI, 0.87-0.88]; prolonged ICU stay: 0.89 [95% CI, 0.88-0.89]; prolonged mechanical ventilation: 0.91 [95% CI, 0.90-0.91]; sepsis: 0.86 [95% CI, 0.85-0.87]; venous thromboembolism: 0.82 [95% CI, 0.81-0.83]; wound complications: 0.78 [95% CI, 0.78-0.79]; 30-day mortality: 0.84 [95% CI, 0.82-0.86]; and 90-day mortality: 0.84 [95% CI, 0.82-0.85]).

**Table 3.** Automated Real-Time Predictions of Postoperative Complications and Outcomes by Number of Input Features in the Random Forest Model

Complication or outcome	AUROC (95% CI)^a^								
	55 features			101 features			135 features		
	Development cohort	Validation cohort	*P* value	Development cohort	Validation cohort	*P* value	Development cohort	Validation cohort	*P* value
Cardiovascular complications	0.83 (0.82-0.83)	0.80 (0.79-0.81)	<.001	0.81 (0.81-0.82)	0.79 (0.78-0.80)	<.001	0.83 (0.82-0.84)	0.81 (0.81-0.82)	<.001
Prolonged ICU stay	0.91 (0.90-0.91)	0.87 (0.87-0.88)	<.001	0.90 (0.90-0.91)	0.87 (0.86-0.87)	<.001	0.92 (0.91-0.92)	0.89 (0.88-0.89)	<.001
Neurological complications, including delirium	0.89 (0.89-0.89)	0.87 (0.86-0.87)	<.001	0.87 (0.86-0.87)	0.85 (0.84-0.86)	<.001	0.89 (0.89-0.90)	0.87 (0.87-0.88)	<.001
Wound complications	0.81 (0.81-0.82)	0.78 (0.77-0.79)	<.001	0.74 (0.74-0.75)	0.71 (0.70-0.72)	<.001	0.80 (0.80-0.81)	0.78 (0.78-0.79)	<.001
Sepsis	0.87 (0.86-0.87)	0.84 (0.83-0.85)	<.001	0.86 (0.86-0.87)	0.84 (0.83-0.85)	<.001	0.87 (0.87-0.88)	0.86 (0.85-0.87)	.002
Venous thromboembolism	0.83 (0.82-0.84)	0.82 (0.81-0.83)	.12	0.81 (0.80-0.82)	0.81 (0.79-0.82)	.42	0.83 (0.82-0.84)	0.82 (0.81-0.83)	.37
Prolonged mechanical ventilation	0.91 (0.90-0.92)	0.90 (0.89-0.91)	.03	0.90 (0.89-0.91)	0.89 (0.88-0.90)	.01	0.92 (0.91-0.92)	0.91 (0.90-0.91)	.11
Acute kidney injury	0.82 (0.82-0.83)	0.81 (0.80-0.81)	<.001	0.82 (0.82-0.83)	0.80 (0.79-0.81)	<.001	0.84 (0.83-0.84)	0.82 (0.82-0.83)	<.001
30-d Mortality	0.86 (0.85-0.87)	0.84 (0.82-0.86)	.05	0.85 (0.84-0.87)	0.84 (0.82-0.86)	.18	0.86 (0.85-0.87)	0.84 (0.82-0.86)	.06
90-d Mortality	0.84 (0.84-0.85)	0.82 (0.81-0.84)	.02	0.84 (0.83-0.85)	0.83 (0.81-0.84)	.34	0.85 (0.84-0.85)	0.84 (0.82-0.85)	.29

Abbreviations: AUROC, area under the receiver operating characteristic curve; ICU, intensive care unit.

^a^ AUROC values with 95% CIs were obtained from bootstrapping with 1000 samples. *P* values comparing AUROC values between the development vs validation cohorts were calculated using the DeLong unpaired method.

A greater number of input features was associated with stable or improved model performance. For example, the model using 135 features to predict prolonged ICU stay achieved an AUROC of 0.89 (95% CI, 0.88-0.89) in the validation cohort, which was significantly greater than the AUROC for the model using 55 features (0.87; 95% CI, 0.87-0.88). The model using 135 features to predict sepsis achieved an AUROC of 0.86 (95% CI, 0.85-0.87) in the validation cohort, which was significantly greater than the AUROC for the model using 55 features (0.84; 95% CI, 0.83-0.85). There were no postoperative complications for which 135 features yielded worse discrimination than 55 features.

In the model using 135 features, AUROC values in the development cohort were greater than those of the validation cohort for predicting cardiovascular complications (0.83 [95% CI, 0.82-0.84] vs 0.81 [95% CI, 0.81-0.82]); prolonged ICU stay (0.92 [95% CI, 0.91-0.92] vs 0.89 [95% CI, 0.88-0.89]); neurological complications, including delirium (0.89 [95% CI, 0.89-0.90] vs 0.86 [95% CI, 0.86-0.87]); wound complications (0.81 [95% CI, 0.81-0.82] vs 0.77 [95% CI, 0.77-0.78]); sepsis (0.87 [95% CI, 0.87-0.88] vs 0.86 [95% CI, 0.85-0.86]); and acute kidney injury (0.84 [95% CI, 0.83-0.84] vs 0.82 [95% CI, 0.82-0.83]), with AUROC improvements ranging from 0.01 for sepsis, venous thromboembolism, prolonged mechanical ventilation, and 90-day mortality to 0.03 for prolonged ICU stay. There was no significant degradation in performance on prospective validation for predicting venous thromboembolism (AUROC, 0.83 [95% CI, 0.82-0.84] vs 0.82 [95% CI, 0.81-0.83]; *P* = .37), prolonged mechanical ventilation (AUROC, 0.92 [95% CI, 0.91-0.92] vs 0.91 [95% CI, 0.90-0.91]; *P* = .11), 30-day mortality (AUROC, 0.86 [95% CI, 0.85-0.87] vs 0.84 [95% CI, 0.82-0.86]; *P* = .06), or 90-day mortality (AUROC, 0.85 [95% CI, 0.84-0.85] vs 0.84 [95% CI, 0.82-0.85]; *P* = .29). The relative contributions of each input feature for each model are shown in eTables 8 to 10 in the Supplement.

### Determination of the Best Model and Feature Set

Comparisons of model AUC values, net reclassification indices, event reclassification fractions, and no-event reclassification fractions are shown in eTable 11 in the Supplement. Overall, the random forest model using 135 input features had similar or greater discrimination and net reclassification indices for all postoperative complications compared with random forest models with smaller feature sets and generalized additive models. For example, it had significantly better discrimination than the generalized additive model using 135 features for prolonged ICU stay (AUROC, 0.89 [95% CI, 0.88-0.89] vs 0.88 [95% CI, 0.87-0.88]; *P* < .001); neurological complications, including delirium (AUROC, 0.87 [95% CI, 0.87-0.88] vs 0.86 [95% CI, 0.86-0.87]; *P* < .001); wound complications (AUROC, 0.78 [95% CI, 0.78-0.79] vs 0.77 [95% CI, 0.77-0.78]; *P* < .001); sepsis (AUROC, 0.86 [95% CI, 0.85-0.87] vs 0.86 [95% CI, 0.85-0.86]; *P* < .001); and acute kidney injury (AUROC, 0.82 [95% CI, 0.82-0.83] vs 0.82 [95% CI, 0.81-0.83]; *P* = .002). In addition to these AUROC values, net reclassification index values for the random forest model using 135 features compared with the random forest model using 55 features were significant for cardiovascular complications (0.015; 95% CI, 0.003-0.027; *P* = .01), prolonged ICU stay (0.025; 95% CI, 0.015-0.035; *P* < .001), venous thromboembolism (0.031; 95% CI, 0.018-0.045; *P* < .001), and acute kidney injury (0.028; 95% CI, 0.017-0.039; *P* < .001). Net reclassification index values for the random forest model using 135 features compared with the generalized additive model using 135 features were significant for prolonged ICU stay (0.024; 95% CI, 0.016-0.033; *P* < .001); neurological complications, including delirium (0.028; 95% CI, 0.019-0.039; *P* < .001); wound complications (0.016; 95% CI, 0.005-0.025; *P* = .002); and prolonged mechanical ventilation (0.021; 95% CI, 0.004-0.038; *P* = .02). Absolute risks for high-risk and low-risk groups are shown in eTable 12 in the Supplement.

### Surgeon Use and Predictions

 A total of 67 surgeons registered for and used the web portal and mobile application. Compared with the original web portal, the mobile device application allowed efficient fingerprint login access and loaded data approximately 10 times faster. In addition to displaying the risk of postoperative complications and the top 3 features associated with the risk of each complication, the output displayed the surgeon’s list of operating room cases, information regarding individual patients, and patterns of complications for individual surgeons compared with their colleagues over time. Model outputs were successfully exported to mobile devices using both iOS (Apple Inc) and Android (Google LLC) operating systems with high speed and fidelity.

There were 193 cases for which an initial surgeon assessment was performed before the algorithms’ risk scores were provided. In a set of 100 cases, surgeons made initial predictions, viewed predictions generated by the algorithm, then made new predictions (surgeon and algorithm predictions are shown in Table 4). Initial surgeon assessments had variable discrimination in predicting postoperative complications, with AUROC values ranging from 0.60 for venous thromboembolism and 0.62 for cardiovascular complications to 0.92 for prolonged ICU stay and wound complications. Compared with initial surgeon assessments, the algorithm had significantly greater discrimination for predicting venous thromboembolism (AUROC, 0.92 [95% CI, 0.85-0.98] vs 0.60 [95% CI, 0.41-0.81]; *P* = .02) and higher but statistically insignificant discrimination for predicting neurological complications, including delirium (AUROC, 0.85 [95% CI, 0.68-0.99] vs 0.82 [95% CI, 0.61-1.00]; *P* = .60); sepsis (AUROC, 0.78 [95% CI, 0.65-0.91] vs 0.74 [95% CI, 0.56-0.89]; *P* = .61); and prolonged mechanical ventilation (AUROC, 0.96 [95% CI, 0.91-1.00] vs 0.80 [95% CI, 0.44-1.00]; *P* = .40). Surgeon predictive performance did not change significantly after viewing predictions generated by the algorithm.

**Table 4.** Surgeon vs Model Discrimination in Predicting Postoperative Complications

Complication	Cases, No.	AUROC (95% CI)			*P* value for surgeons’ initial assessments vs model predictions^a^	*P* value for surgeons’ postviewing assessments vs model predictions^a^	*P* value for surgeons’ initial vs postviewing assessments^a^
		Surgeons’ assessments before viewing model predictions	Model predictions	Surgeons’ assessments after viewing model predictions			
Cardiovascular complications	100	0.62 (0.45-0.78)	0.49 (0.31-0.67)	0.62 (0.45-0.78)	.43	.28	.35
Prolonged ICU stay	100	0.92 (0.83-0.99)	0.86 (0.75-0.96)	0.92 (0.83-0.99)	.14	.14	>.99
Neurological complications, including delirium	100	0.82 (0.61-1.00)	0.85 (0.68-0.99)	0.76 (0.61-0.91)	.60	.01	.33
Wound complications	100	0.92 (0.86-0.97)	0.90 (0.84-0.96)	0.92 (0.86-0.97)	.65	.65	>.99
Sepsis	100	0.74 (0.56-0.89)	0.78 (0.65-0.91)	0.74 (0.56-0.89)	.61	.61	.48
Venous thromboembolism	100	0.60 (0.41-0.81)	0.92 (0.85-0.98)	0.60 (0.40-0.81)	.02	.02	.48
Prolonged mechanical ventilation	100	0.80 (0.44-1.00)	0.96 (0.91-1.00)	0.80 (0.44-1.00)	.40	.39	>.99
Acute kidney injury	97	0.78 (0.65-0.88)	0.66 (0.49-0.82)	0.77 (0.65-0.88)	.12	.12	.41

Abbreviations: AUROC, area under the receiver operating characteristic curve; ICU, intensive care unit.

^a^ *P* values comparing AUROC values were calculated using the DeLong unpaired method.

## Discussion

In this prognostic study involving a prospective cohort of patients receiving major inpatient surgical procedures, the platform accurately predicted postoperative complications using automated real-time EHR data and mobile device outputs. Previous versions of the platform exhibited good predictive accuracy using retrospective data while providing model outputs to a web portal.^15^ The current study built on those results by finding minimal performance degradation during prospective validation and by providing model outputs to mobile devices with efficient fingerprint login access, faster data loading, and expanded outputs that included patterns of postoperative complications for individual surgeons compared with their colleagues over time. For most complications, random forest models outperformed generalized additive models, and a greater number of input features was associated with stable or improved model performance. Increasing the number of input features can become tedious and inefficient when clinicians must manually enter features.^13^ Therefore, the platform automatically imported EHR data and included as many input variables as would augment model performance without substantially increasing the model footprint or training time. The best model had predictive performance matching that of surgeons.

Other data-based approaches to predicting postoperative complications have reported accuracy, precision, and external validity, but few have optimized interpretability by conveying the relative importance of model inputs in determining outputs, and none have incorporated both automated data acquisition and mobile device outputs.^14,30,31,32,33,34^ The American College of Surgeons National Surgical Quality Improvement Program surgical risk calculator^30^ is the most prominent and well-validated data-based method for predicting postoperative complications. The American College of Surgeons risk calculator maintains data security and interoperability by presenting users with an online platform for manual data entry. However, lack of clinical workflow integration and automaticity have been deterrents for physician use of surgical decision-support platforms.^13^ Meguid et al^34^ began working toward automated clinical integration by developing a regression-based calculator that predicted postoperative complications using 8 input features; most of these features could be automatically accrued from EHRs. Bertsimas et al^14^ developed an optimal classification tree algorithm that made data-based predictions with discrimination slightly greater than those made by the American College of Surgeons risk calculator and did so through a mobile application. Although the application required manual data entry, the algorithm adapted to each entry to minimize the number of input variables required, rarely requiring more than 10 manual inputs. To our knowledge, MySurgeryRisk is the only published platform that accurately predicts postoperative complications with fully automated data entry and mobile device outputs; many major health care systems are already capable of extracting data from EHRs and providing surgeon-level analytics, suggesting the potential generalizability of this approach.

To achieve real-time automated data acquisition and provide outputs to mobile devices, we expanded and enhanced the previously reported^15^ system architecture as a scalable real-time platform. The previously reported web-based user interface lacked a message-pushing mechanism to provide timely model outputs to physicians, and its data visualizations did not scale well to small screens on mobile devices. The mobile application resolved these issues. The mobile application’s security was enhanced with options for personal identification number and biometric fingerprint security authentication. In addition, the mobile application collected and stored physicians’ predictions before and after they viewed the algorithm’s predictions, which may facilitate future studies assessing the impact of algorithm predictions for surgical decision-making in clinical settings. The application also displayed patterns of postoperative complications for individual surgeons compared with their colleagues over time, which could be used for data-based quality improvement initiatives.

### Limitations

This study has several limitations. The primary limitation of the platform is the lack of external validation. To achieve external validity, the platform’s automated input features will need to be mapped to interoperable common data standards. In addition, predictions made with machine learning methods rely on associations between outcomes and inputs rather than causality. Although our algorithm provides data on feature importance for each postoperative complication for each patient, this approach explains only how predictions occurred and does not identify which features may have caused the complication.

Differences between the performance of model predictions and physicians’ initial assessments before viewing model outputs did not reach statistical significance because of the small sample size and the high variability within a sample that may not be representative of the whole cohort. Physician’s predictive performance did not change significantly after viewing model outputs, suggesting opportunities to improve the clinical impact of model predictions, especially when model discrimination is greater than that of physicians (as observed in the prediction of venous thromboembolism). Information provided by the platform is unlikely to augment decision-making, mitigation of modifiable risk factors, or prognostication among experienced, highly skilled surgeons who already make highly accurate predictions of postoperative complications.

To avoid the creation of biases and inequalities in surgical care, risk prediction algorithms need to use unbiased source data and variables. The fairness of surgical risk calculators, including our algorithm, has been questioned but not formally tested.^35^ Therefore, future research may seek to achieve data and algorithm interoperability and fairness.

## Conclusions

In this prognostic study, postoperative complications were accurately predicted by an artificial intelligence system using automated real-time EHR data, with minimal performance degradation during prospective validation and accuracy that matched surgeons’ predictions. Predictive performance was optimized by the use of larger input feature sets and random forest architectures that accurately represented complex nonlinear associations among features. To facilitate integration with clinical workflow, model outputs were provided to mobile device applications. To our knowledge, this system is the only one to accurately predict postoperative complications with fully automated data acquisition and mobile device outputs. Further work is necessary to achieve data and algorithm interoperability and fairness.

## Notes

Supplement. **eMethods.** Source of Data, Participants, Outcomes, Predictor Variables, Data Transformer, Optimization of Surgical Procedure Codes, Calculation of Risk Score, Model Performance, General Additive Model, Random Forests Classifier, Real-Time Intelligent Perioperative Platform, and Analytic Workflow of MySurgeryRisk **eFigure 1.** Flow Diagram of MySurgeryRisk Model Development and Validation Cohorts **eFigure 2.** MySurgeryRisk System Design **eFigure 3.** Initial Signup Page of the MySurgeryRisk Web Platform **eFigure 4.** Postlogin Page of the MySurgeryRisk Web Platform **eFigure 5.** Risk Assessment Page of the MySurgeryRisk Web Platform **eFigure 6.** Risk Assessment Comparison Page of the MySurgeryRisk Web Platform **eFigure 7.** Outcome Dashboard Page of the MySurgeryRisk Web Platform **eFigure 8.** Postlogin Page of the MySurgeryRisk Mobile App **eFigure 9.** Risk Assessment Page of the MySurgeryRisk Mobile App **eFigure 10.** Patient Similarity Comparison Page of the MySurgeryRisk Mobile App **eFigure 11.** Outcome Dashboard Page of the MySurgeryRisk Mobile App **eTable 1.** Input Features Used in Versions **eTable 2.** Development and Validation Cohort Characteristics of Features Used for MySurgeryRisk Model Development **eTable 3.** Generalized Additive Model Performance Measurements for Complications With 95% CIs Calculated by 1000 Bootstrap Samples on the Validation Cohort **eTable 4.** Summary of the Contributions of Each Input Feature to Each Outcome for a Generalized Additive Model With 55 Input Features **eTable 5.** Summary of the Contributions of Each Input Feature to Each Outcome for a Generalized Additive Model With 101 Input Features **eTable 6.** Summary of the Contributions of Each Input Feature to Each Outcome for a Generalized Additive Model With 135 Input Features **eTable 7.** Random Forest Model Performance Measurements for Complications With 95% CIs Calculated by 1000 Bootstrap Samples on the Validation Cohort **eTable 8.** Summary of the Contributions of Each Input Feature to Each Outcome for a Random Forest Model With 55 Input Features **eTable 9.** Summary of the Contributions of Each Input Feature to Each Outcome for a Random Forest Model With 105 Input Features **eTable 10.** Summary of the Contributions of Each Input Feature to Each Outcome for a Random Forest Model With 135 Input Features **eTable 11.** Comparison of Validation Cohort Model Performance With Respect to AUROC, Net Reclassification Index, Percentage of Event Reclassification, and Percentage of No Event Reclassification **eTable 12.** Absolute and Relative Risk Associated With High-Risk and Low-Risk Groups for Postoperative Complications With 95% CIs Calculated by 1000 Bootstrap Samples on the Validation Cohort
eReferences
