# Supplementary material for: Performance of a Machine Learning Algorithm Using Electronic Health Record Data to Predict Postoperative Complications and Report on a Mobile Platform
Source: JAMA Netw Open. 2022 May 16;5(5):e2211973. doi: 10.1001/jamanetworkopen.2022.11973 (PMC9112066; doi:10.1001/jamanetworkopen.2022.11973)
Supplement: Supplement. — eMethods. Source of Data, Participants, Outcomes, Predictor Variables, Data Transformer, Optimization of Surgical Procedure Codes, Calculation of Risk Score, Model Performance, General Additive Model, Random Forests Classifier, Real-Time Intelligent Perioperative Platform, and Analytic Workflow of MySurgeryRisk eFigure 1. Flow Diagram of MySurgeryRisk Model Development and Validation Cohorts eFigure 2. MySurgeryRisk System Design eFigure 3. Initial Signup Page of the MySurgeryRisk Web Platform eFigure 4. Postlogin Page of the MySurgeryRisk Web Platform eFigure 5. Risk Assessment Page of the MySurgeryRisk Web Platform eFigure 6. Risk Assessment Comparison Page of the MySurgeryRisk Web Platform eFigure 7. Outcome Dashboard Page of the MySurgeryRisk Web Platform eFigure 8. Postlogin Page of the MySurgeryRisk Mobile App eFigure 9. Risk Assessment Page of the MySurgeryRisk Mobile App eFigure 10. Patient Similarity Comparison Page of the MySurgeryRisk Mobile App eFigure 11. Outcome Dashboard Page of the MySurgeryRisk Mobile App eTable 1. Input Features Used in Versions eTable 2. Development and Validation Cohort Characteristics of Features Used for MySurgeryRisk Model Development eTable 3. Generalized Additive Model Performance Measurements for Complications With 95% CIs Calculated by 1000 Bootstrap Samples on the Validation Cohort eTable 4. Summary of the Contributions of Each Input Feature to Each Outcome for a Generalized Additive Model With 55 Input Features eTable 5. Summary of the Contributions of Each Input Feature to Each Outcome for a Generalized Additive Model With 101 Input Features eTable 6. Summary of the Contributions of Each Input Feature to Each Outcome for a Generalized Additive Model With 135 Input Features eTable 7. Random Forest Model Performance Measurements for Complications With 95% CIs Calculated by 1000 Bootstrap Samples on the Validation Cohort eTable 8. Summary of the Contributions of Each Input Feature to Each Outcome for a Random Forest Model [file jamanetwopen-e2211973-s001.pdf]

Supplementary Online Content

Ren Y, Loftus TJ, Datta S, et al. Performance of a machine learning algorithm using electronic health record data to predict postoperative complications and report on a mobile platform. *JAMA Netw Open*. 2022;5(5):e2211973. doi:10.1001/jamanetworkopen.2022.11973

eMethods. Source of Data, Participants, Outcomes, Predictor Variables, Data Transformer, Optimization of Surgical Procedure Codes, Calculation of Risk Score, Model Performance, General Additive Model, Random Forests Classifier, Real-Time Intelligent Perioperative Platform, and Analytic Workflow of MySurgeryRisk

eFigure 1. Flow Diagram of MySurgeryRisk Model Development and Validation Cohorts

eFigure 2. MySurgeryRisk System Design

eFigure 3. Initial Signup Page of the MySurgeryRisk Web Platform

eFigure 4. Postlogin Page of the MySurgeryRisk Web Platform

eFigure 5. Risk Assessment Page of the MySurgeryRisk Web Platform

eFigure 6. Risk Assessment Comparison Page of the MySurgeryRisk Web Platform

eFigure 7. Outcome Dashboard Page of the MySurgeryRisk Web Platform

eFigure 8. Postlogin Page of the MySurgeryRisk Mobile App

eFigure 9. Risk Assessment Page of the MySurgeryRisk Mobile App

eFigure 10. Patient Similarity Comparison Page of the MySurgeryRisk Mobile App

eFigure 11. Outcome Dashboard Page of the MySurgeryRisk Mobile App

eTable 1. Input Features Used in Versions

eTable 2. Development and Validation Cohort Characteristics of Features Used for MySurgeryRisk Model Development

eTable 3. Generalized Additive Model Performance Measurements for Complications With 95% CIs Calculated by 1000 Bootstrap Samples on the Validation Cohort

eTable 4. Summary of the Contributions of Each Input Feature to Each Outcome for a Generalized Additive Model With 55 Input Features

eTable 5. Summary of the Contributions of Each Input Feature to Each Outcome for a Generalized Additive Model With 101 Input Features

eTable 6. Summary of the Contributions of Each Input Feature to Each Outcome for a Generalized Additive Model With 135 Input Features

eTable 7. Random Forest Model Performance Measurements for Complications With 95% CIs Calculated by 1000 Bootstrap Samples on the Validation Cohort

eTable 8. Summary of the Contributions of Each Input Feature to Each Outcome for a Random Forest Model With 55 Input Features

eTable 9. Summary of the Contributions of Each Input Feature to Each Outcome for a Random Forest Model With 105 Input Features

eTable 10. Summary of the Contributions of Each Input Feature to Each Outcome for a Random Forest Model With 135 Input Features

© 2022 Ren Y et al. *JAMA Network Open*.

eTable 11. Comparison of Validation Cohort Model Performance With Respect to AUROC, Net Reclassification Index, Percentage of Event Reclassification, and Percentage of No Event Reclassification

eTable 12. Absolute and Relative Risk Associated With High-Risk and Low-Risk Groups for Postoperative Complications With 95% CIs Calculated by 1000 Bootstrap Samples on the Validation Cohort

eReferences

This supplementary material has been provided by the authors to give readers additional information about their work.

© 2022 Ren Y et al. *JAMA Network Open*.

eMethods. Source of Data, Participants, Outcomes, Predictor Variables, Data Transformer, Optimization of Surgical Procedure Codes, Calculation of Risk Score, Model Performance, General Additive Model, Random Forests Classifier, Real-Time Intelligent Perioperative Platform, and Analytic Workflow of MySurgeryRisk

Source of Data

Using the University of Florida Health (UFH) Integrated Data Repository as Honest Broker for data de-identification we have created a single-center perioperative longitudinal cohort that integrated electronic health record (EHR) data with administrative datasets as previously described.[1,2] Using residency zip code, we linked the cohort with the United State Census data[3,4] to calculate residing neighborhood characteristics and distance from hospital.[5] We included all inpatient operative procedures performed between June 1, 2014 and September 20, 2020. The date of death was determined using hospital records and the search of the Social Security Death Index.[6]

Participants

We included all patients with age greater or equal to 18 years admitted between June 1, 2014 and September 20, 2020 with the admission involving any type of inpatient operative procedure. Detailed exclusion criteria are shown in **eFigure 1**. When a patient had multiple surgeries during one admission, only the first surgery was used in the analysis. The final retrospective cohort consisted of 41,812 patients undergoing 52,117 surgeries; the prospective cohort consisted of 19,132 patients undergoing 22,300 surgeries.

Outcomes

We have developed and implemented an array of AI algorithms for perioperative real-time data integration, harmonization and preprocessing, computable phenotyping and dynamic perioperative risk assessment for eight postoperative complications including prolonged (>48 hours) intensive care unit (ICU) stay and mechanical ventilation (MV), neurological complications including delirium, cardiovascular complications, acute kidney injury (AKI), venous thromboembolism (VTE), sepsis, and wound complications that include infectious and mechanical wound complications.[2,7,8] We used the exact dates to calculate the duration of MV and ICU stay. We defined AKI using KDIGO consensus criteria[9] while a set of previously described criteria was applied to annotate the remaining complications. The algorithm also calculates risk probabilities for death at 30 days and 90 days 1, 3, 6, 12 and 24 months after index surgery.

We report six *MySurgeryRisk* model versions, including three generalized additive models and three random forest models using 55, 101 and 135 input features (**eTable 11**). These models followed the same rules for data preprocessing, feature selection, and model development methods as previously described.[8]

Predictor Variables

From 285 available preoperative demographic, socio-economic, administrative, clinical, pharmacy and laboratory variables we have derived the set of preoperative predictor features. For each patient we considered all potential preoperative predictors available in the harmonized dataset. Patient comorbidities were derived using up to 50 preoperative The International Classification of Diseases (ICD), Ninth Revision, Clinical Modification (ICD-9-CM) and the International Classification of Diseases, Tenth Revision, Clinical Modification (ICD-10-CM) codes. We used validated methods to define binary preoperative comorbidity variables [11,12] and composite Charlson comorbidity index. [13] We extracted medications dispensed in the one year prior to surgery day using RxNorms data grouped into drug classes according to the United States Department of Veterans Affairs National Drug File-Reference Terminology; medications were derived from RxNorm codes grouped into drug classes as previously described. [14] We modeled primary procedure type on Current Procedural Terminology (CPT) with a forest structure in which nodes represented groups of procedures, roots presented the most general groups of procedures, and leaf nodes represented specific procedures.

Supervised feature selection was performed using the variance inflation factors to evaluate collinearity and remove highly collinear predictors (VIF package in R [10]). Due to the nature of clinical data many predictors are derived from a single attribute thus the likelihood of collinearity among variables is high.

Data Transformer

MySurgeryRisk transforms data from any native EHR format to the processed dataset optimized for use in predictive models. New complex variables are created (as described above in "*Predictor Variables*") and are used in data preprocessing, feature transformation and feature selection.

Data preprocessing included data cleaning, outlier removal and missing data imputation. [15,16] For initial data cleaning we tagged the inconclusive test results as missing. Set of automatic rules was used for the removal of outliers that were considered unreasonable observations by medical experts. For continuous variables, observations that fell in the top and bottom 1% of the distribution were considered as outliers, and they were imputed with a random number generated from the range between 0.5th and 5th percentiles and between 95th and 99.5th percentiles, respectively. All missing observations were imputed using automated algorithm. For nominal variables with missing entries, a distinct "missing" category was created. For continuous variables, the mean value for a given variable was used for imputation.

Feature transformation was applied to reduce dimensionality of the data and decrease the chance of overfitting. We optimized categorical and nominal variables with multiple levels (such as zip code or surgeon's ID) using conditional probabilities for a patient to have a particular variable value conditioning on each outcome separately [17]. We substituted the values x_i of categorical variables with the ratios

$$\log[\mathrm{P}(X_i = x_i \mid C = 1\,) \,/\, \mathrm{P}(X_i = x_i \mid C = 0)]$$

where

$$\mathrm{P}(X_i = x \mid C = c) = \#\{j : C^j = c, x_i^j = x\}/\#\{j : C^j = c\}$$

and then treated each categorical variable as an ordered variable. In case of classification trees such substitution gives the optimal splits, in terms of cross-entropy or Gini index. Based on our experiments, such modeling of categorical variables provides less overfitting than when using binary dummy variables (data not shown).

In order to obtain a reliable estimate of $\mathrm{P}(X_i = x \mid C = c)$, categorical risk factors with categories with fewer than 100 records were grouped together and labeled "other". This "other" group was further split into several subgroups where each subgroup contained categories with similar proportions of patients from different classes. This was achieved by performing k-means clustering[18] on the set of categories in the "other" group. We set the number of clusters to 2 if there were at least 3 categories in the "other" group; otherwise we set it to 1 (no clustering). The computations were implemented in python software using the kmeans function from scipy package. The rational for this approach was to reduce the over-fitting of the data, since large clusters are less affected by random data splits, than small individual categories. Computational results showed that indeed this technique improved the stability of the model and reduced the effects of over-fitting.

Surgical procedure codes were optimized using forest of trees approach to group Current Procedural Terminology (CPT) codes [17] followed by a conditional probability calculation approach described above. Each node represents a group of procedures, with roots representing most general groups of procedures and leaf nodes representing specific procedures.

Optimization of Surgical Procedure Codes

The types of surgical procedures were determined using the primary CPT processing codes. The existing ~ 9000 codes are prefix-based on anatomical location of surgery and often lack detailed descriptions of surgical approach. Although they are important features for risk stratification, their high dimensionality renders them challenging for the use in predictive models. In addition, while for some procedure codes only a few patients were encountered in the cohort, the estimation of probabilities by counting the number of such patients in each class would be unreliable. To overcome this issue we combined procedures with small number of patients into groups of procedures based on their similarity according to the CPT classification using forest of trees approach as previously described [17] and then optimized the CPT codes using condition probability described above. We created a hierarchical tree of CPT, which is structured by levels. Level 0 (root) represents all CPT codes; level 1 contains Clinical Classifications Software

© 2022 Ren Y et al. *JAMA Network Open*.

(CCS)[19] codes; level 2 contains ranges of CPT codes and level 3 contains specific CPT codes. For example, “ALL”, “CCS 1”, “61000-61050” and “61000” is an example of the path from root to the leaf node. We added a node of ‘Missing’ to level 1 of this tree. For each CPT code without matched CCS code, we generated a branch of ‘Missing’ node at level 1 with the CPT code itself as the nodes at level 2 as well as level 3. For each leaf node, we assigned a number of patients who had a type of surgical procedure described by this node’s code, and for each non-leaf node (such nodes represent general classes of procedures) we assigned a number of patients whose type of surgical procedure belongs to this class. Procedures were aggregated up to the top level of the CPT hierarchy such that each procedure/group of procedures contained at least 100 patients. The value of this parameter was selected based on a grid search through the values 50, 100, 150, 250 and 500. To assign continuous values to CPT codes, we located the leaf nodes containing CPT codes and find the continuous value using the log of conditional odds of positive outcome described above.

Calculation of Risk Score

A set of algorithms was trained to calculate patient-level risk probabilities for each of eight complications. The calculated risk probabilities were subsequently used as input data for the algorithm trained to calculate mortality risk scores. The final output produces *MySurgeryRisk*, a personalized risk panel for eight major complications and mortality risk at 30 and 90 days after surgery together with a list of the top three features contributing to each of the calculated risk scores.

Patient-Level Risk Scores, representing the probability of each complication during hospitalization after index surgery, were calculated. For each complication separately, we used risk probabilities calculated by the algorithm to define the optimal cutoff values that best categorize patients into low and high risk categories by maximizing the Youden index.[20] The most important features contributing to the risk for an individual patient were derived based on how different she or he is from the patient with an “average” risk. Features that contribute the most to a high or low risk score for a particular complication in a specific patient were determined by creating an average patient for each complication. For categorical features, numerical values were assigned to each class and then features for average patient were set to an actually observed value, closest to the median value among all patients of each feature. Next, each feature of the average patient was replaced with the value of that feature from a certain patient and the change in the risk score was calculated. This procedure was repeated for all features one at a time and features with the highest positive and negative change were determined as the features contributing the most to a high and low risk score, respectively.

Patient-Level Mortality Scores, representing the probability of death at 30 and 90 days after index surgery, were calculated using a random forests classifier trained over the individual complication risk probabilities within a 5-fold cross validation design.[21] We automatically tuned the parameters for each classifier through maximizing accuracy as the cross validation performance score over searching a parameter space.

Model Performance

We assessed each model’s discrimination using the AUROC and model accuracy by determining the fraction of correct classification for each model. Using the optimal thresholds for risk probabilities we built the classification table from which we calculated sensitivity, specificity, and positive and negative predictive values for each model. Relative risk was calculated as the ratio of the absolute risk of the complication for high and low risk groups for each complication. We used bootstrap sampling and nonparametric methods to obtain 95% confidence intervals for all performance measures.

Generalized Additive Model

We estimated the probability of outcome ($C = 1$, otherwise $C = 0$) by using a generalized additive model:

$$\text{logit } \mathrm{P}(C = 1 | X = x) = \alpha + \sum_{i=1}^{m} f_i\,(x_i) \qquad (1)$$

where m is the number of risk factors, $X = (X_1, \dots, X_m)$ are the risk factors, $x = (x_1, \dots, x_m)$ are the values of these factors, f_i is a nonlinear risk function associated with the ith risk factor and α is a free term. Nonlinear risk functions f_i were estimated for each feature with cubic splines via a local scoring algorithm [22]. The degrees of freedom for

© 2022 Ren Y et al. *JAMA Network Open*.

each spline were estimated by maximizing restricted likelihood function [23]. Degrees of freedom characterize a curvature of a spline, with value 1 corresponding to a linear function. Risk predictors with estimated degrees of freedom close to 1 were not smoothed in the final model; instead the original values of risk predictors x_i were used. Therefore, the final model has the following form as in equation (2) where $\bar{I}$ is a set of risk predictors with estimated degrees of freedom close to 1 and w_i is the linear weight of the ith risk predictors.

$$\text{logit } \mathrm{P}(E = 1|X = x) = \alpha + \sum_{i \in \bar{I}} w_i * x_i + \sum_{i \in \underline{I}} f_i\,(x_i) \qquad (2)$$

Random Forests Classifier

Random forests (RF) is a machine learning method which generates and uses a collection of numerous classification and regression trees (CART) and aggregates their results [24]. Each of the generated classifiers are trained on a bootstrap sample of the training data. Each generated tree in the random forests predicts the outcome and then the model generates the final outcome through aggregating the outcomes of all trees using majority voting of the trees. Random forests employ bootstrap aggregation [25] and random feature selection [26] to construct a set of decision trees, thus, applying controlled variation. [27] We used the R implementation of the random forests classifier. [28] We used the mean decrease in accuracy measure to determine feature importance. In this approach, a feature is determined to be more important if removing it from the random forests causes a greater decrease in accuracy.

The Real-Time Intelligent Perioperative Platform

Web Application Design

The previously reported *MySurgeryRisk* output platform was a secure web interface.[8] The web application is based on Tomcat and Java Jersey EE frameworks. The front-end interfaces are written with PHP, HTML, and relative languages; the back-end follows RESTful APIs. The *MySurgeryRisk* web portal uses a Shibboleth identification service for authorization and is confined to the University of Florida Health secure network, which is accessible offsite through a virtual private network. The web portal provides surgeons with their patients' demographic information, planned surgery, risk factors, and predicted probabilities for postoperative complications and mortality (**eFigures 2-6**). Surgeons can record their own predictions for complications and mortality before and after viewing model predictions.

Mobile Device Application Design

The *MySurgeryRisk* mobile application performs all the same functions as the website, with the convenience of being accessible on mobile devices. In addition, the mobile application identifies similar patients from the retrospective database, and provides surgeons with risk profiles for those patients. Similar patients are identified using primary surgeon performing the operation, primary procedure code, patient demographics, and patient Charleston Comorbidity Index. The application has two modes: 1) data collection mode, in which surgeons make their own risk assessments before and after viewing model risk assessments, and 2) view only mode, in which surgeons view model predictions without submitting their own risk assessments.

The application is built using the dart programming language and Flutter framework. It run on iOS, android, and macOS with plans to expand to run on Windows operating systems and the Web. The application has been designed and built with speed and security in mind. All data is received and transmitted with secure socket layer (SSL) encryption. The mobile application uses a Shibboleth identification service for authorization and is confined to the University of Florida Health secure network, accessible offsite through a virtual private network. Users can also choose to configure a pin code or biometric security (fingerprint or face scan) for efficient login access. The mobile application loads data approximately ten times faster than the web portal. Application screenshots are provided in e**Figure 2** and **eFigures 8-11**. The output displays a list of operating room cases, information regarding each individual patient, each patient's risk for eight postoperative complications plus 30-day and 90-day mortality, the top three features conferring risk for those complications, and complication trends for individual surgeons compared with their colleagues over time.

Analytic Workflow of MySurgeryRisk

MySurgeryRisk is an automated EHR algorithm that will be implemented in real-time using the intelligent perioperative platform developed by our group.[29] This platform resides in a secure environment and in real time integrates and transforms EHR data, runs predictive algorithms, produces outputs for physicians, inputs their feedback and prospectively collects data for the future retraining of the prediction models.

The *MySurgeryRisk* platform consists of three main components, including Permanent Data Store, FHIR Adapter, and Intelligent Engine, as previously described. The Permanent Data Store directly stores the data from hospital data warehouse and other sources through FHIR adapter then applying standard data model OMOP to formulate the uniformed clinical database for analysis. The FHIR adapter works as the hub of data exchanging which provides Restful API and format transforming services. It not only serves as the messaging protocol for other components of the platform but also expose the data services for applications with the widely accepted interpretable specification. The Intelligent Engine receives the transformed data through FHIR adapter and performs computational algorithms to compute patient-level risk scores for complications and mortality. It provides containerized development environments for researchers to develop their models and pipelines and easy final deployment in a real-time productional setting. The *MySurgeryRisk* platform architecture is illustrated in e**Figure 2**. We use the NoSQL database Cassandra to store large volumes of intermediate and output data for streaming services. Finally, for the Application platform, we developed a web portal and mobile device application to provide real-time displays of risk scores, as described in detail below. To comply with the Health Insurance Portability and Accountability Act, authorized surgeons can only access the web portal and mobile application within the UF Health virtual private network, which requires multiple layers of authorization to maintain system security. All predictions are saved in the Cassandra database for future comparative studies.

© 2022 Ren Y et al. *JAMA Network Open*.

eFigure 1. Flow Diagram of MySurgeryRisk Model Development and Validation Cohorts

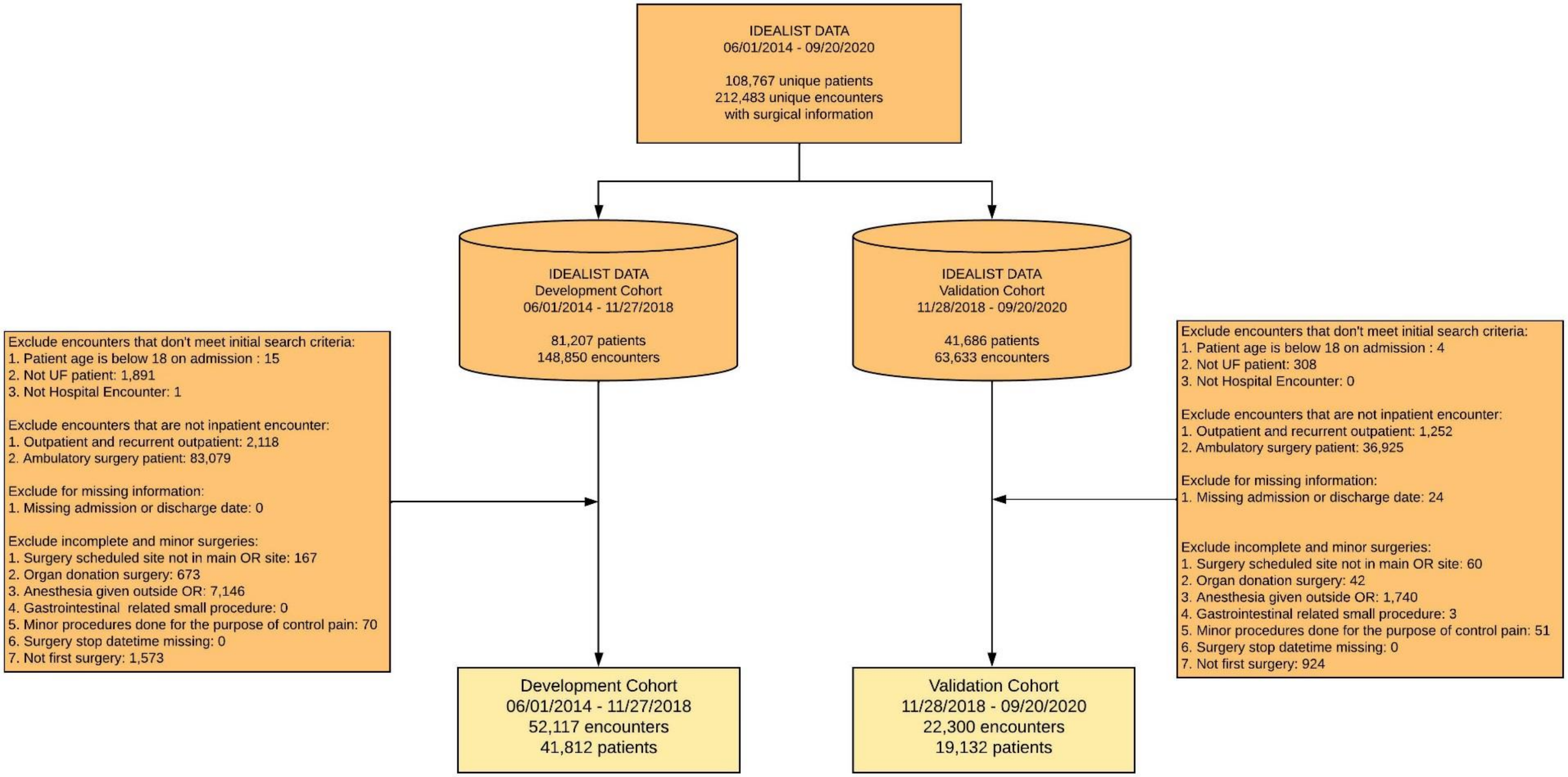

© 2022 Ren Y et al. *JAMA Network Open*.

eFigure 2. MySurgeryRisk System Design

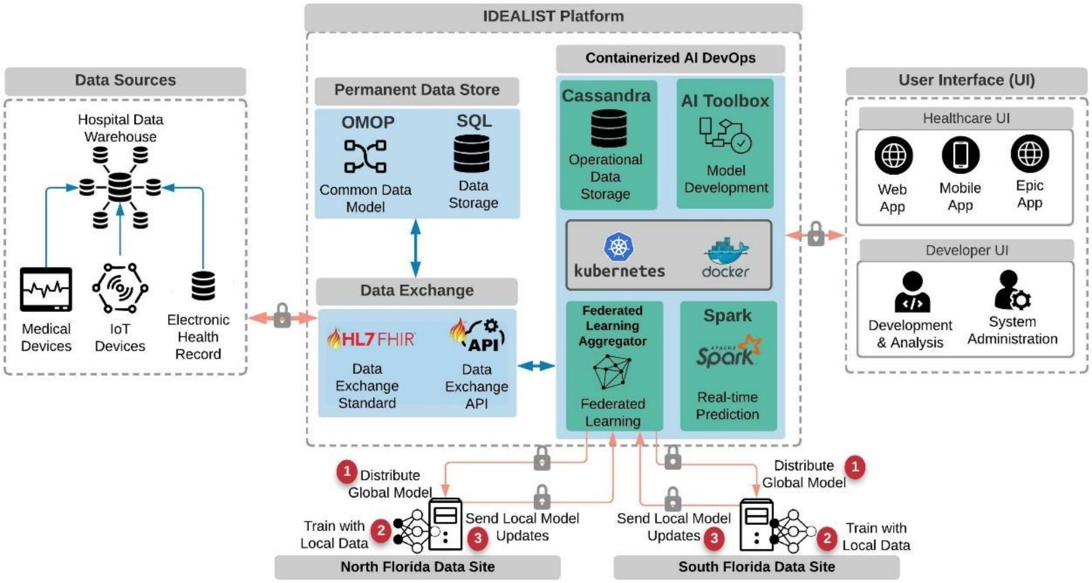

The Integrated Data Repository (IDR) supplies raw data, which is supplied to the intelligent engine using the Kafka messaging protocol. All Analytics Engine components are deployed as Docker containers to isolate each component so that the system does not need to be reconfigured with each deployment in a new data environment. The NoSQL database Cassandra is used to store large volumes of intermediate and output data for streaming services, deploying the web portal and mobile device applications via a web server.

© 2022 Ren Y et al. *JAMA Network Open*.

eFigure 3. Initial Signup Page of the MySurgeryRisk Web Platform

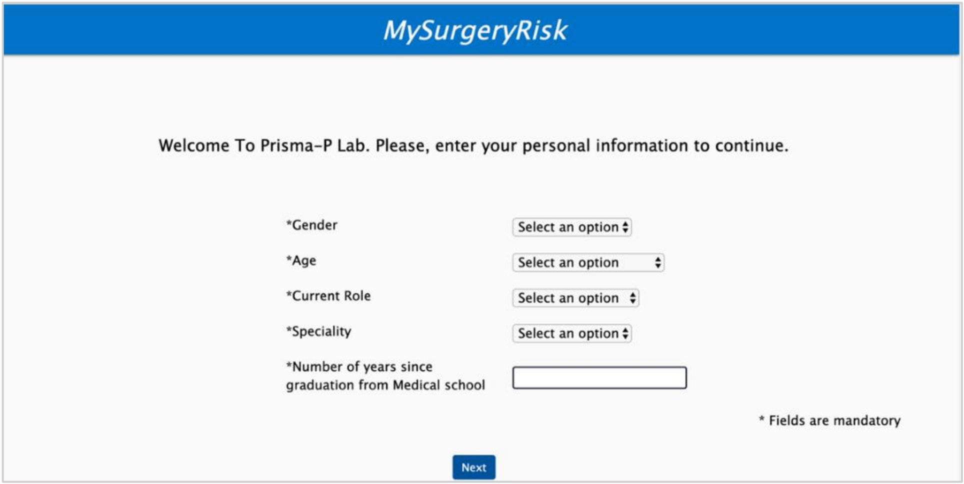

First-time users are greeted with a registration page on which they complete a user agreement and configure their doctor profile

© 2022 Ren Y et al. *JAMA Network Open*.

eFigure 4. Postlogin Page of the MySurgeryRisk Web Platform

(A)

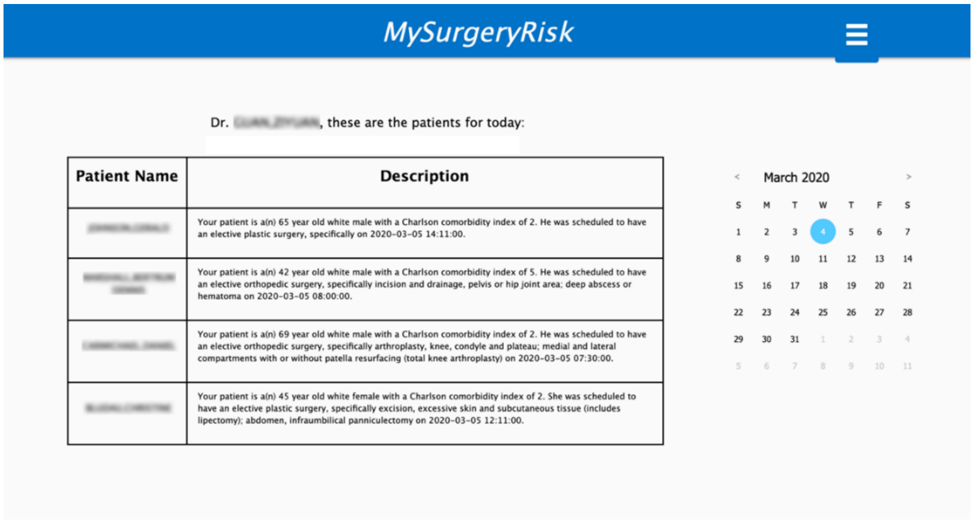

(B)

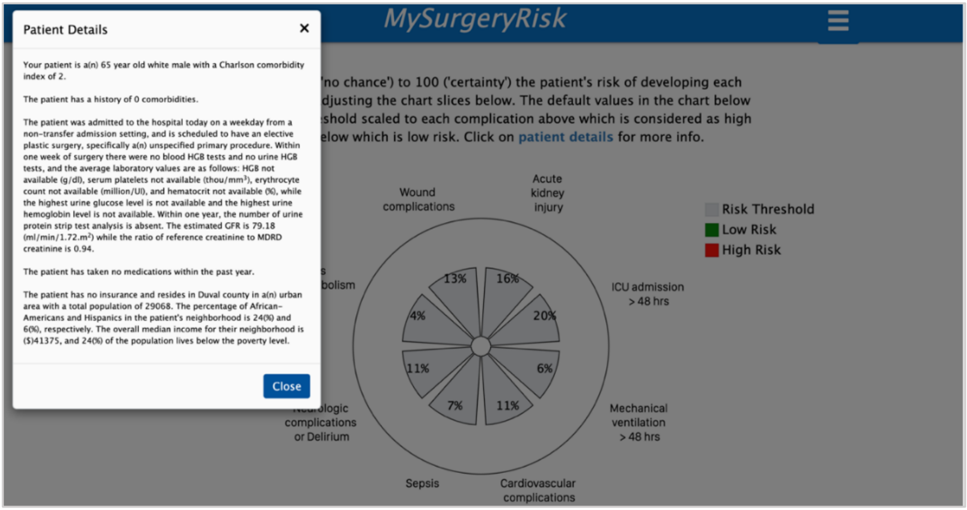

The page starts with an opening page with records of patients (A). Tapping a patient's information leads to details for the patients, including age, gender, surgery information, lab results, and comorbidity information (B).

© 2022 Ren Y et al. *JAMA Network Open*.

eFigure 5. Risk Assessment Page of the MySurgeryRisk Web Platform

(A)

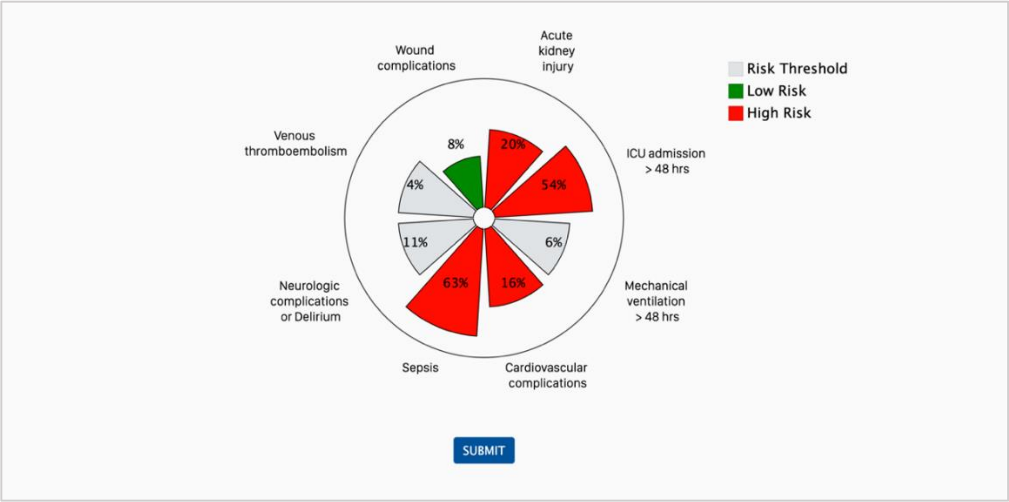

(B)

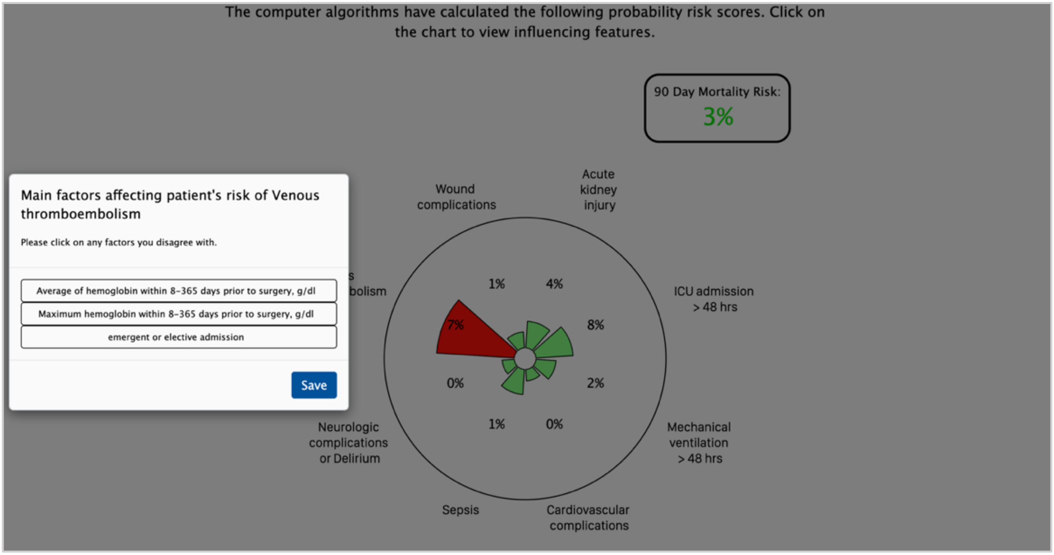

The webpage provides opportunity to the physicians to make their risk assessment of a patient based on the provided information (A). Following the risk assessment from the physicians, the app reveals risk predictions from the algorithm along with major factors leading to the prediction (B).

© 2022 Ren Y et al. *JAMA Network Open*.

eFigure 6. Risk Assessment Comparison Page of the MySurgeryRisk Web Platform

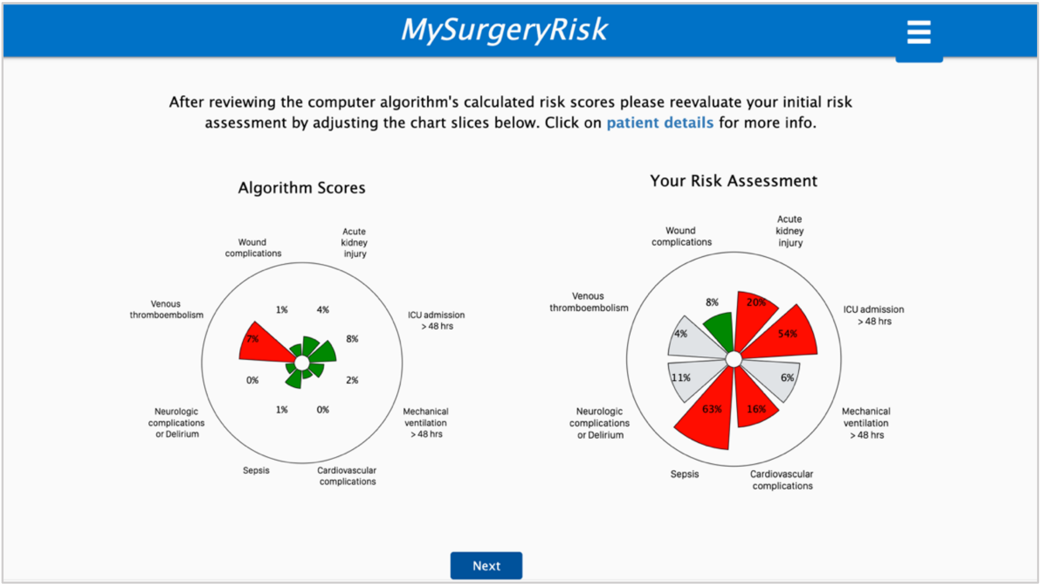

The webpage provides opportunity to the physicians to compare their risk assessment with algorithm's assessment. Physicians are also given an opportunity to adjust in their predictions after seeing the algorithm predictions, if deemed necessary.

eFigure 7. Outcome Dashboard Page of the MySurgeryRisk Web Platform

(A)

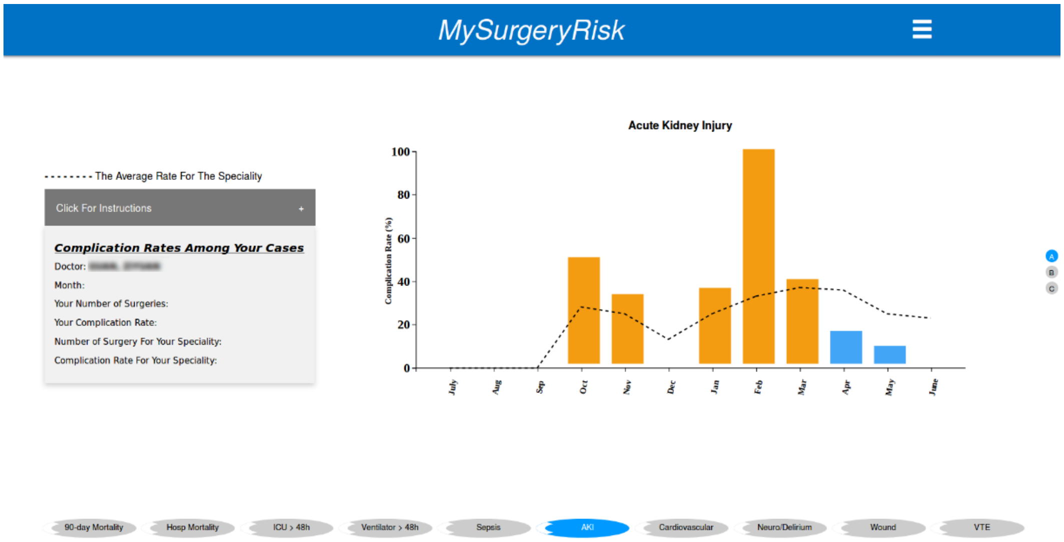

(B)

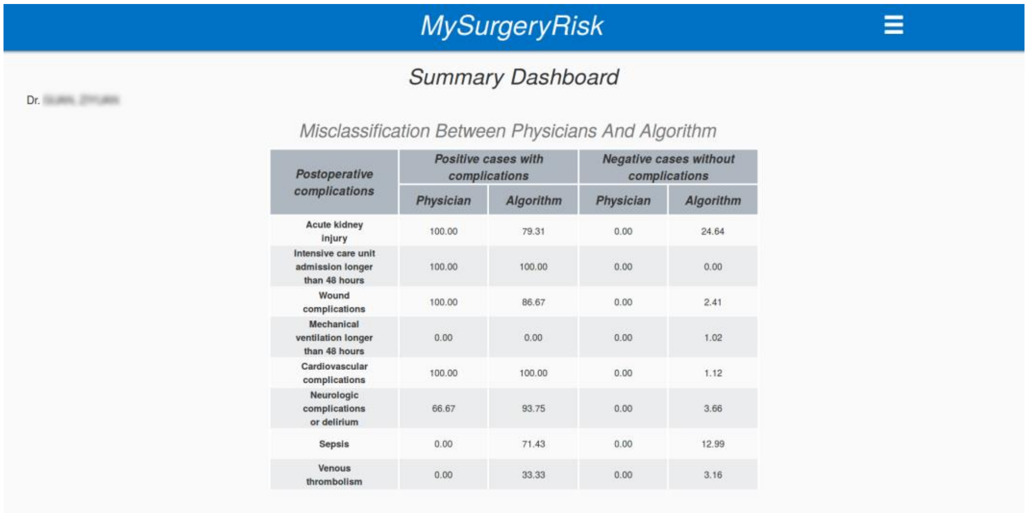

Postoperative complications	Positive cases with complications		Negative cases without complications	
	Physician	Algorithm	Physician	Algorithm
Acute kidney injury	100.00	79.31	0.00	24.64
Intensive care unit admission longer than 48 hours	100.00	100.00	0.00	0.00
Wound complications	100.00	86.67	0.00	2.41
Mechanical ventilation longer than 48 hours	0.00	0.00	0.00	1.02
Cardiovascular complications	100.00	100.00	0.00	1.12
Neurologic complications or delirium	66.67	93.75	0.00	3.66
Sepsis	0.00	71.43	0.00	12.99
Venous thrombolism	0.00	33.33	0.00	3.16

The webpage provides detailed information on each complication, including total patients in the specialty, number of patients with complication, percentage of patients with complication in a monthly basis (A). Physicians can also compare their misclassification rates with the algorithm performance (B).

© 2022 Ren Y et al. *JAMA Network Open*.

eFigure 8. Postlogin Page of the MySurgeryRisk Mobile App

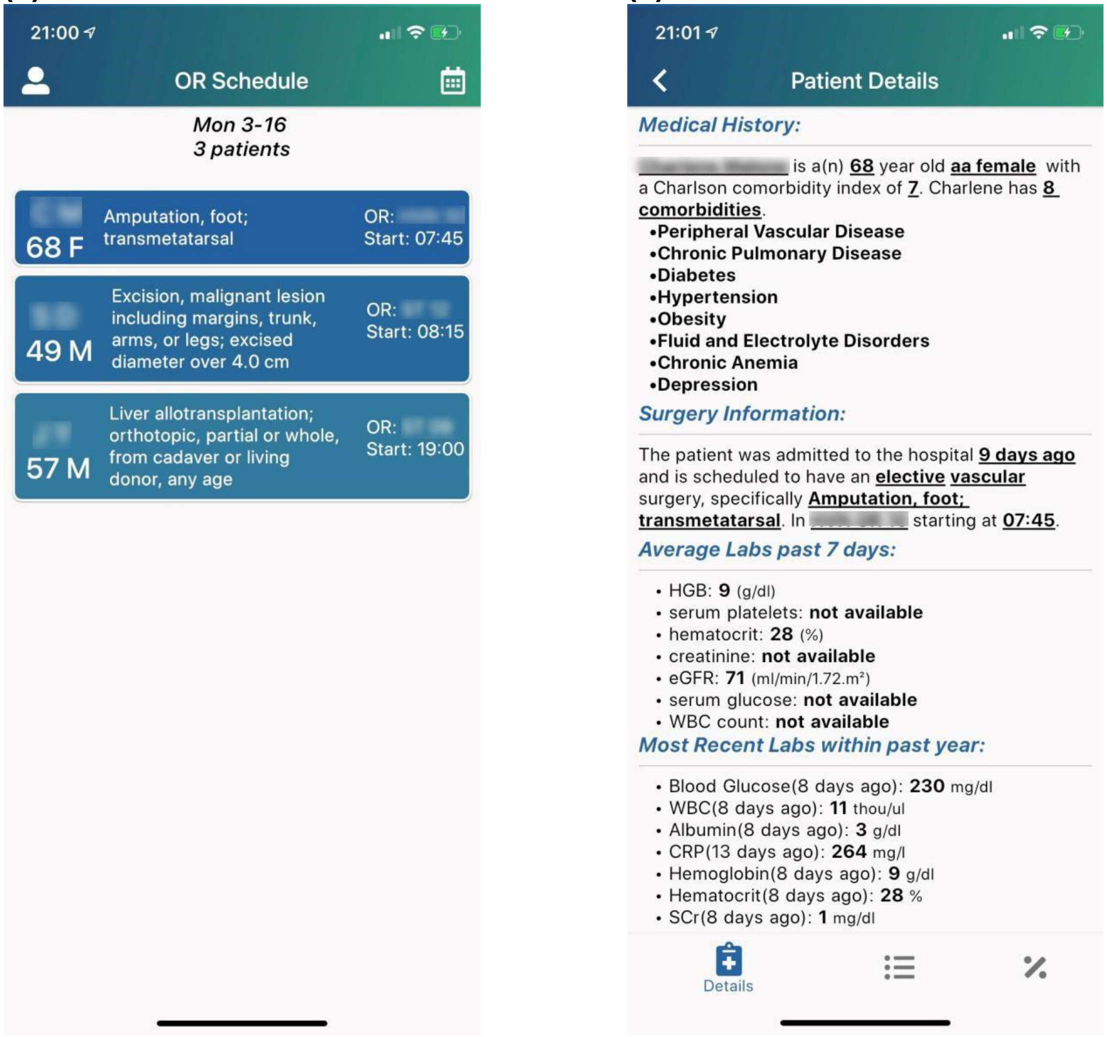

The app starts with an opening page with records of patients (A). Tapping a patient's information leads to details for the patients, including age, gender, surgery information, lab results, and comorbidity information (B).

© 2022 Ren Y et al. *JAMA Network Open*.

eFigure 9. Risk Assessment Page of the MySurgeryRisk Mobile App

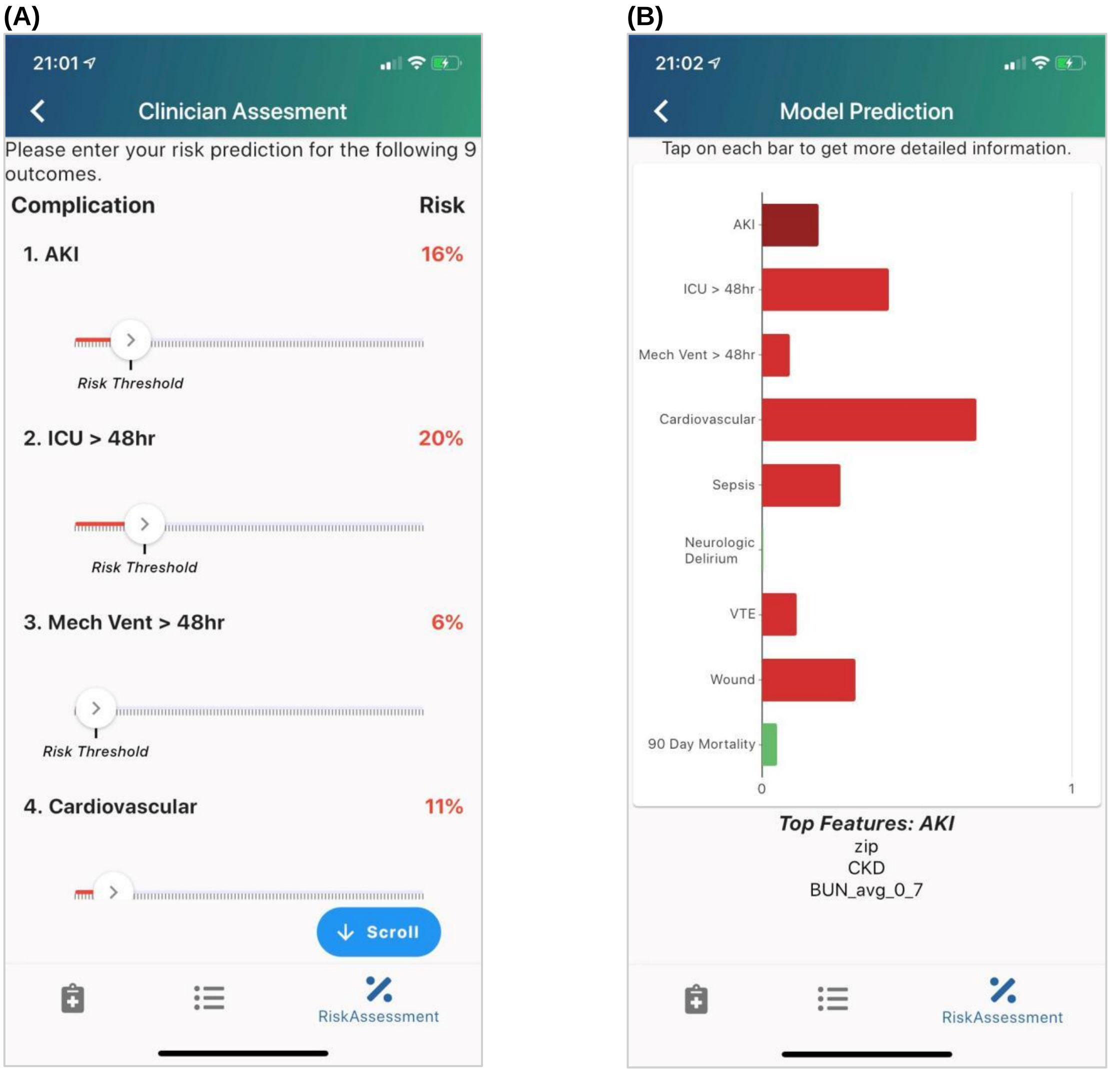

The app provides opportunity to the physicians to make their risk assessment of a patient based on the provided information (A). Following the risk assessment from the physicians, the app reveals risk predictions from the algorithm along with major factors leading to the prediction (B).

© 2022 Ren Y et al. *JAMA Network Open*.

eFigure 10. Patient Similarity Comparison Page of the MySurgeryRisk Mobile App

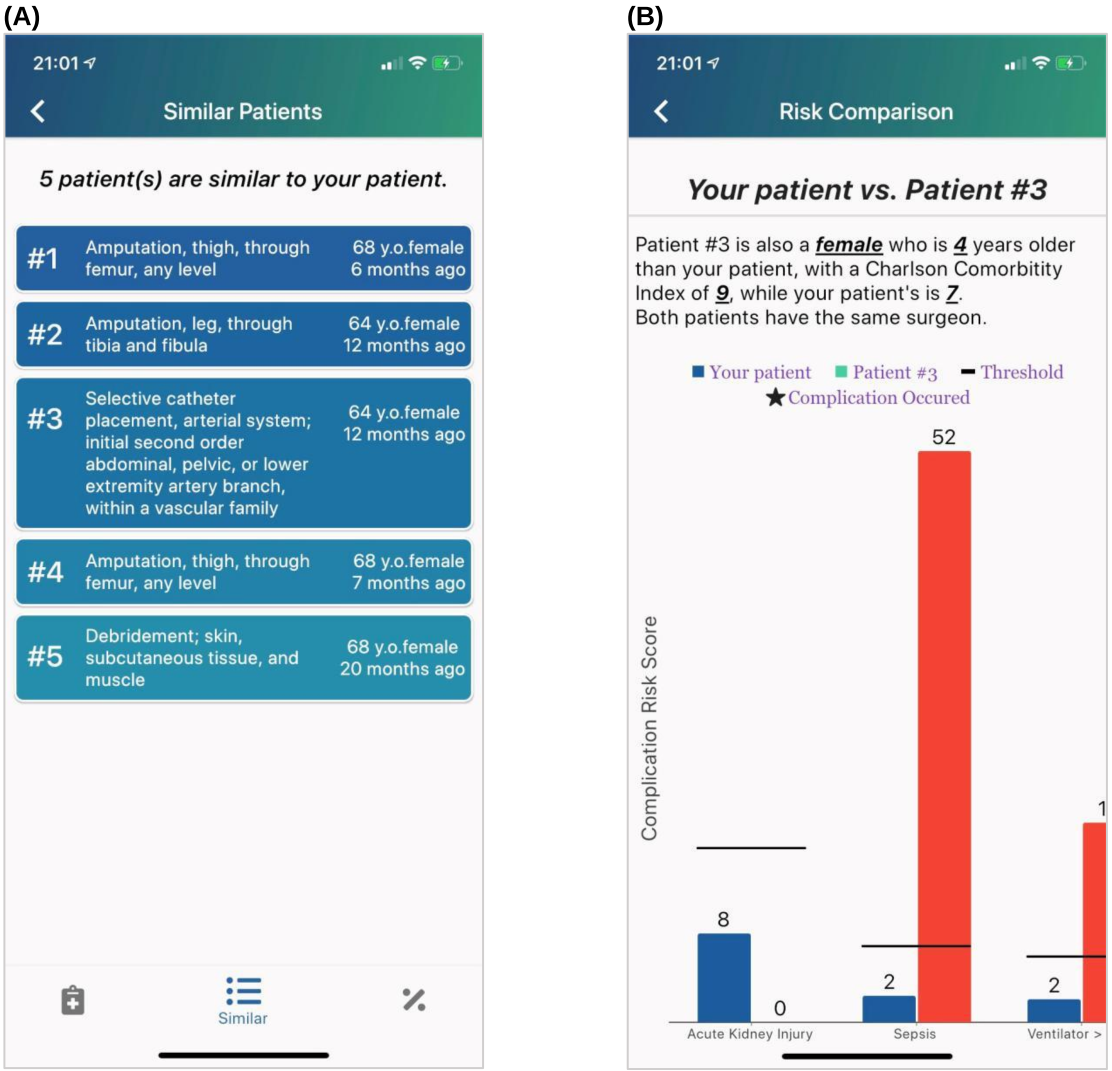

The app provides opportunity to the physicians to see the list of previous patients with similar clinical conditions (A). Physicians can see side-by-side comparison of risk scores for current and previous patients (B).

© 2022 Ren Y et al. *JAMA Network Open*.

eFigure 11. Outcome Dashboard Page of the MySurgeryRisk Mobile App

(A) (B)

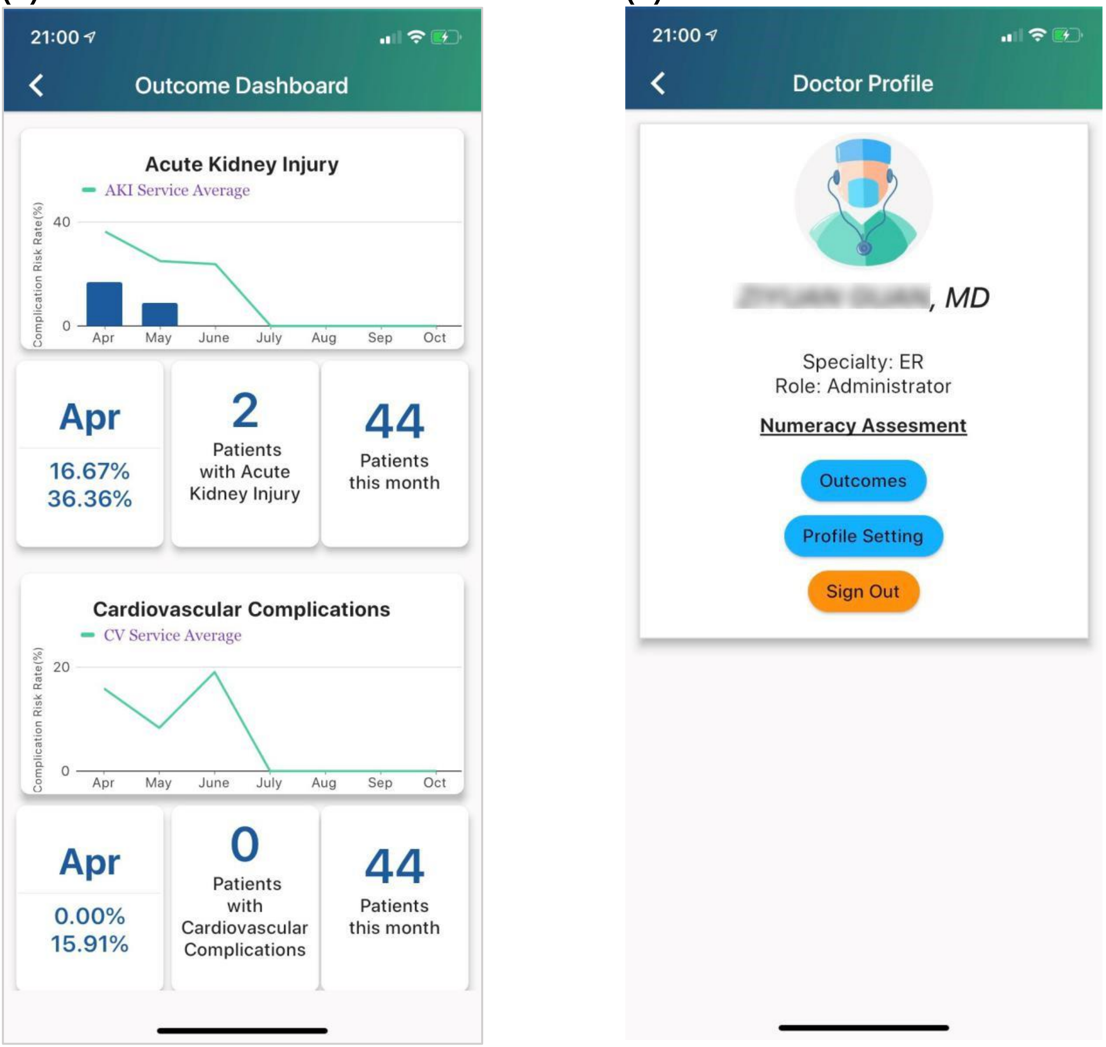

The app provides detailed information on each complication, including total patients in the specialty, number of patients with complication, percentage of patients with complication in a monthly basis (A). Physicians can also see their risk assessment history from *Outcomes* tab in their profile page (B).

© 2022 Ren Y et al. *JAMA Network Open*.

eTable 1. Input Features Used in Versions

	Number of input features in the model					
**Feature**	55	101	135	55 (type)	101 (type)	135 (type)
Sex	X	X	X	Binary	Binary	Binary
Native Language Spoken		X	X		Binary	Binary
Scheduled post operation location		X	X		Binary	Binary
Scheduled room is trauma room or not		X	X		Binary	Binary
rural or city at patient residential area	X	X	X	Binary	Binary	Binary
emergent or elective admission	X	X	X	Binary	Binary	Binary
Anesthesia Type		X	X		Binary	Binary
Yes, if the admission we happened at night		X	X		Binary	Binary
CKD status at admission		X	X		Categorical (Binary for AKI)	Categorical (Binary for AKI)
Myocardial Infarction	X		X	Binary		Binary
Congestive Heart Failure	X		X	Binary		Binary
Cerebrovascular Disease	X		X	Binary		Binary
Chronic Pulmonary Disease	X		X	Binary		Binary
Peripheral Vascular Disease	X		X	Binary		Binary
Cancer	X		X	Binary		Binary
Liver Disease	X		X	Binary		Binary
Valvular Disease	X		X	Binary		Binary
Coagulopathy	X		X	Binary		Binary
Weight Loss	X		X	Binary		Binary
alcohol abuse or drug	X		X	Binary		Binary
Fluid and electrolyte disorders			X			Binary
Chronic anemia	X		X	Binary		Binary
Hypertension	X		X	Binary		Binary
Obesity			X			Binary
Diabetes	X		X	Binary		Binary
Metastatic Carcinoma			X			Binary
Depression			X			Binary
Indicator of receiving Betablockers within one year before admission date	X	X	X	Binary	Binary	Binary
Indicator of receiving Diuretics within one year before admission date	X	X	X	Binary	Binary	Binary

© 2022 Ren Y et al. *JAMA Network Open*.

	Number of input features in the model					
**Feature**	55	101	135	55 (type)	101 (type)	135 (type)
Indicator of receiving statin within one year before admission date	X	X	X	Binary	Binary	Binary
Indicator of receiving Aspirin within one year before admission date	X		X	Binary		Binary
Indicator of receiving ACE Inhibitors within one year before admission date	X	X	X	Binary	Binary	Binary
Indicator of receiving vasopressors or inotropes within one year before admission date	X	X	X	Binary	Binary	Binary
Indicator of receiving Bicarbonate within one year before admission date	X	X	X	Binary	Binary	Binary
Indicator of receiving Antiemetic within one year before admission date	X	X	X	Binary	Binary	Binary
Indicator of receiving Aminoglycosides within one year before admission date	X		X	Binary		Binary
Indicator of receiving Vancomycin within one year before admission date	X		X	Binary		Binary
No of nephrotoxic drugs received within one year before admission date			X			Binary
Nonsteroidal anti-inflammatory drugs	X			Binary		
Race	X	X	X	Categorical	Categorical	Categorical
Ethnicity		X	X		Categorical	Categorical
Insurance paying the bills	X	X	X	Categorical	Categorical	Categorical
Admission Source	X	X	X	Categorical	Binary	Binary
Scheduled surgery room		X	X		Categorical	Categorical
Smoking Status	X	X	X	Categorical	Categorical	Categorical
Automated urinalysis, urine protein presence within 365 days prior to surgery, mg/dL	X		X	Categorical		Categorical
Automated urinalysis, urine glucose within 7 days prior to surgery, mg/dL	X			Categorical		

© 2022 Ren Y et al. *JAMA Network Open*.

	Number of input features in the model					
**Feature**	55	101	135	55 (type)	101 (type)	135 (type)
Number of urine glucose tests within 0-7 days prior to surgery		X			Categorical	
Automated urinalysis, urine glucose within 8-365 days prior to surgery, mg/dL		X			Categorical	
Number of urine glucose tests within 8-365 days prior to surgery		X			Categorical	
Automated urinalysis, urine hemoglobin within 7 days prior to surgery, mg/dL	X		X	Categorical		Categorical
Automated urinalysis, urine hemoglobin within 8-365 days prior to surgery, mg/dL		X	X		Categorical	Categorical
Number of urine hemoglobin tests within 8-365 days prior to surgery		X	X		Categorical	Categorical
Surgery Type	X	X	X	Categorical	Categorical	Categorical
Marital Status		X	X		Categorical	Categorical
ZIP Code of home address	X	X	X	Categorical	Categorical	Categorical
County of home address	X	X	X	Categorical	Categorical	Categorical
Admission Day	X		X	Categorical		Categorical
Admission Month	X	X	X	Categorical	Categorical	Categorical
ID of Attending Surgeon	X	X		Categorical	Categorical	
medicine or surgery admitting	X	X	X	Categorical	Categorical	Categorical
Charlson comorbidity index	X	X	X	Categorical	Categorical	Categorical
Current Procedural Terminology code of the primary procedure	X		X	Categorical		Categorical
Age	X	X	X	Numerical	Numerical	Numerical
Body Mass Index		X	X		Numerical	Numerical
Distance of residence to hospital, km	X	X	X	Numerical	Numerical	Numerical
total population at patient residential area	X			Numerical		
median total income at patient residential area, USD	X	X	X	Numerical	Numerical	Numerical

© 2022 Ren Y et al. *JAMA Network Open*.

	Number of input features in the model					
**Feature**	55	101	135	55 (type)	101 (type)	135 (type)
prevalence of African American residents living below poverty at patient residential area, %	X		X	Numerica l		Numerical
prevalence of Hispanic residents living below poverty at patient residential area, %	X		X	Numerica l		Numerical
Prevalence of residents living below poverty at patient residential area, %	X		X	Numerica l		Numerical
Time from Admission to Surgery, days	X	X	X	Numerica l	Numerical	Numerical
Reference estimated glomerular filtration rate	X		X	Numerica l		Numerical
Min hemoglobin within 7 days prior to surgery, g/dl	X		X	Numerica l		Numerical
Max hemoglobin within 7 days prior to surgery, g/dl		X	X		Numerical	Numerical
Average of hemoglobin within 7 days prior to surgery, g/dl		X	X		Numerical	Numerical
Variance of hemoglobin within 7 days prior to surgery, g/dl		X	X		Numerical	Numerical
Number of hemoglobin tests within 7 days prior to surgery	X		X	Numerica l		Numerical
Min hemoglobin within 8-365 days prior to surgery, g/dl		X	X		Numerical	Numerical
Max hemoglobin within 8-365 days prior to surgery, g/dl		X	X		Numerical	Numerical
Variance of hemoglobin within 8-365 days prior to surgery, g/dl		X			Numerical	
Average of hemoglobin within 8-365 days prior to surgery, g/dl		X	X		Numerical	Numerical
Number of hemoglobin tests within 8-365 days prior to surgery		X	X		Numerical	Numerical
Min of Serum Calcium, mmol/L		X	X		Numerical	Numerical
Max of Serum Calcium, mmol/L			X			Numerical
Average of Serum Calcium, mmol/L		X	X		Numerical	Numerical
Variance of Serum Calcium, mmol/L		X	X		Numerical	Numerical
Count of Serum Calcium test		X	X		Numerical	Numerical
Min of anion gap in blood, mmol/L		X			Numerical	

© 2022 Ren Y et al. *JAMA Network Open*.

	Number of input features in the model					
**Feature**	55	101	135	55 (type)	101 (type)	135 (type)
Average of anion gap in blood, mmol/L		X	X		Numerical	Numerical
Count of anion gap in blood test		X	X		Numerical	Numerical
Min of White Blood Cell in blood, thou/uL			X			Numerical
Max of White Blood Cell in blood, thou/uL		X	X		Numerical	Numerical
Average of White Blood Cell in blood, thou/uL		X	X		Numerical	Numerical
Variance of White Blood Cell in blood, thou/uL			X			Numerical
Count of White Blood Cell in blood test		X	X		Numerical	Numerical
Max of Hematocrit in blood, %		X			Numerical	
Min of Hematocrit in blood, %		X	X		Numerical	Numerical
Average of Hematocrit in blood, %		X	X		Numerical	Numerical
Variance of Hematocrit in blood, %		X	X		Numerical	Numerical
Count of Hematocrit in blood test		X	X		Numerical	Numerical
Max of Serum Red Blood Cell, Million/uL			X			Numerical
Average of Serum Red Blood Cell, Million/uL			X			Numerical
Average of Mean Corpuscular Volume in blood, fL		X			Numerical	
Max of the amount of hemoglobin relative to the size of the cell in blood, g/dL			X			Numerical
Average of the amount of hemoglobin relative to the size of the cell in blood, g/dL		X	X		Numerical	Numerical
Count of the amount of hemoglobin relative to the size of the cell in blood test		X			Numerical	
Min of Mean platelet volume in blood, fL		X			Numerical	
Average of Mean platelet volume in blood, fL		X			Numerical	
Min of Glucose in blood, mg/dL		X	X		Numerical	Numerical
Max of Glucose in blood, mg/dL		X	X		Numerical	Numerical
Average of Glucose in blood, mg/dL		X	X		Numerical	Numerical

© 2022 Ren Y et al. *JAMA Network Open*.

	Number of input features in the model					
**Feature**	55	101	135	55 (type)	101 (type)	135 (type)
Variance of Glucose in blood, mg/dL		X			Numerical	
Count of Glucose in blood test		X	X		Numerical	Numerical
Min of Serum CO2 , mmol/L		X	X		Numerical	Numerical
Max of Serum CO2, mmol/L		X	X		Numerical	Numerical
Average of Serum CO2, mmol/L		X	X		Numerical	Numerical
Variance of Serum CO2, mmol/L		X	X		Numerical	Numerical
Count of Serum CO2 test		X	X		Numerical	Numerical
Min of Urea nitrogen in blood, mg/dL		X	X		Numerical	Numerical
Max of Urea nitrogen in blood, mg/dL		X	X		Numerical	Numerical
Average of Urea nitrogen in blood, mg/dL		X	X		Numerical	Numerical
Variance of Urea nitrogen in blood, mg/dL		X	X		Numerical	Numerical
Count of Urea nitrogen in blood test		X	X		Numerical	Numerical
Min of Urea Nitrogen-Creatinine ratio		X	X		Numerical	Numerical
Max of Urea Nitrogen-Creatinine ratio		X	X		Numerical	Numerical
Average of Urea Nitrogen-Creatinine ratio		X	X		Numerical	Numerical
Variance of Urea Nitrogen-Creatinine ratio		X	X		Numerical	Numerical
Count of Urea Nitrogen-Creatinine ratio		X	X		Numerical	Numerical
Min of Serum Sodium, mmol/L		X			Numerical	
Max of Serum Sodium, mmol/L		X	X		Numerical	Numerical
Variance of Serum Sodium, mmol/L		X			Numerical	
Average of Serum Sodium, mmol/L		X	X		Numerical	Numerical
Count of Serum Sodium test		X	X		Numerical	Numerical
Average of Potassium in serum, mmol/L		X	X		Numerical	Numerical
Count of Potassium in serum test		X	X		Numerical	Numerical
Max of Red cell distribution width in Blood, %		X	X		Numerical	Numerical
Min of Red cell distribution width in Blood, %		X			Numerical	

© 2022 Ren Y et al. *JAMA Network Open*.

	Number of input features in the model					
**Feature**	55	101	135	55 (type)	101 (type)	135 (type)
Average of Red cell distribution width in Blood, %		X	X		Numerical	Numerical
Variance of Red cell distribution width in Blood, %			X			Numerical
Min of platelet in blood, thou/uL			X			Numerical
Max of platelet in blood, thou/uL		X	X		Numerical	Numerical
Average of platelet in blood, thou/uL		X	X		Numerical	Numerical
Variance of platelet in blood, thou/uL		X	X		Numerical	Numerical
Min of Serum creatinine, mg/dL			X			Numerical
Max of Serum creatinine, mg/dL			X			Numerical
Average of Serum creatinine, mg/dL			X			Numerical
Variance of Serum creatinine, mg/dL			X			Numerical
Count of Serum creatinine test			X			Numerical
Max of chloride in Serum, mmol/L			X			Numerical
Average of chloride in Serum, mmol/L			X			Numerical
Variance of chloride in Serum, mmol/L			X			Numerical
Count of chloride in Serum test			X			Numerical

‘X’ marks use of the feature.
Abbreviations: ACE, Angiotensin-converting enzyme; CKD, chronic kidney disease.

© 2022 Ren Y et al. *JAMA Network Open*.

eTable 2. Development and Validation Cohort Characteristics of Features Used for MySurgeryRisk Model Development

Features	Development Cohort	Validation Cohort
**Number of patients, n**	41,812	19,132
**Demographic information**		
Age, years, mean (SD)[a]	56 (18)	58 (17)
Sex, n (%)		
Male	20,830 (49.8)	9,672 (50.6)
Female	20,982 (50.2)	9,460 (49.4)
Race, n (%)[b]		
African American	6,225 (14.9)	2,765 (14.5)
White	32,286 (77.2)	14,777 (77.2)
Other[c]	2,667 (6.4)	1,235 (6.5)
Missing	634 (1.5)	355 (1.9)
Ethnicity, n (%)[b]		
Non-Hispanic	39,067 (93.4)	17,663 (92.3)
Hispanic	1,987 (4.8)	979 (5.1)
Missing	758 (1.8)	490 (2.6)
Native Language Spoken, n (%)		
English	41,034 (98.1)	18,769 (98.1)
Non-English	778 (1.9)	363 (1.9)
Marital Status, n (%)[a]		
Married	19,940 (47.7)	8,986 (47.0)
Single	15,362 (36.7)	7,303 (38.2)
Divorced	6,190 (14.8)	2,709 (14.2)
Missing	320 (0.8)	134 (0.7)
Insurance paying the bills, n (%)[a]		
Medicare	18,451 (44.1)	9,183 (48.0)
Private	13,255 (31.7)	5,447 (28.5)
Medicaid	6,727 (16.1)	2,757 (14.4)
Uninsured	3,379 (8.1)	1,745 (9.1)
**Number of encounters, n**	52,117	22,300
**Socio-economical information**		
Distance of residence to hospital, km, mean (SD)	71 (119)	71 (113)
Rural at patient residential area, n (%)	17,979 (34)	7,807 (35)
Total population at patient residential area, mean (SD)	20,579 (13,440)	20,475 (13,324)
Median total income at patient residential area, USD, mean (SD)	43,129 (12,440)	43,323 (12,550)
Prevalence of residents living below poverty at patient residential area, %, mean (SD)	20.27 (9.65)	19.98 (9.35)
Prevalence of African American residents living below poverty at patient residential area, %, mean (SD)	0.16 (0.16)	0.16 (0.16)
Prevalence of Hispanic residents living below poverty at patient residential area, %, mean (SD)	0.08 (0.08)	0.08 (0.07)
Smoking Status, n (%)		
Never	22,640 (43)	9,932 (45)
Former	17,571 (34)	7,578 (34)

© 2022 Ren Y et al. *JAMA Network Open*.

Features	Development Cohort	Validation Cohort
Current	9,400 (18)	3,779 (17)
Missing	2,506 (5)	1,011 (5)
Body Mass Index, median (IQR)	28 (24, 33)	28 (24, 33)
**Surgical information, n (%)**		
Time from Admission to Surgery, days, median (IQR)	3 (2, 21)	3 (2, 23)
Emergency admission	18,753 (36)	8,457 (38)
The admission happened at night	24,128 (46)	10,546 (47)
Admission type		
Surgery	20,630 (40)	8,769 (39)
Medicine	22,363 (43)	8,931 (40)
Transferred from another hospital	7,770 (15)	3,582 (16)
Anesthesia Type		
General	47,492 (91)	20,538 (92)
Local/regional	4,625 (9)	1,762 (8)
Scheduled room is trauma room	359 (1)	139 (1)
Scheduled post operation location is ICU	10,668 (20)	2,441 (11)
Scheduled surgery room		
1st Rank	3,285 (6)	1,141 (5)
2nd Rank	2,976 (6)	1,115 (5)
3rd Rank	2,748 (5)	1,053 (5)
Attending Surgeon		
1st Rank	1,522 (3)	619 (3)
2nd Rank	1,393 (3)	544 (2)
3rd Rank	1,187 (2)	512 (2)
Current Procedural Terminology code of the primary procedure		
1st Rank	2,213 (4)	709 (3)
2nd Rank	1,832 (4)	641 (3)
3rd Rank	1,490 (3)	615 (3)
Admission Day		
Monday	10,107 (19)	4,411 (20)
Tuesday	9,932 (19)	4,201 (19)
Wednesday	8,970 (17)	3,878 (17)
Sunday	8,605 (17)	3,752 (17)
Thursday	8,421 (16)	3,424 (15)
Friday	3,285 (6)	1,355 (6)
Saturday	2,797 (5)	1,279 (6)
Admission Month		
Aug	4,987 (10)	2,083 (9)
Jul	4,836 (9)	2,230 (10)
Jun	4,855 (9)	2,105 (9)

© 2022 Ren Y et al. *JAMA Network Open*.

**Features**	**Development Cohort**	**Validation Cohort**
Oct	5,118 (10)	1,169 (5)
Sep	4,662 (9)	1,613 (7)
Mar	3,995 (8)	2,046 (9)
Jan	3,919 (8)	2,063 (9)
May	3,791 (7)	2,173 (10)
Feb	3,854 (7)	2,015 (9)
Dec	3,809 (7)	1,951 (9)
Nov	4,440 (9)	1,147 (5)
Apr	3,851 (7)	1,705 (8)
Surgery Type		
Orthopedic surgery	12,335 (24)	5,381 (24)
Neurosurgery	7,082 (14)	3,469 (16)
Vascular surgery	5,092 (10)	2,233 (10)
Urology	4,697 (9)	1,613 (7)
Ear Nose Throat	3,723 (7)	1,343 (6)
Cardio Thoracic Surgery	3,563 (7)	1,098 (5)
Gastrointestinal Surgery	3,089 (6)	1,258 (6)
OB Gynecology	2,173 (4)	685 (3)
Surgical Oncology	2,038 (4)	545 (2)
Other[d]	8,325 (16)	4,675 (21)
**Medication within one year before admission date, n (%)**		
Number of nephrotoxic drugs received, median (IQR)	0 (0, 1)	0 (0, 1)
ACE Inhibitors	4,917 (9)	1,930 (9)
Aminoglycosides	1,885 (4)	994 (4)
Antiemetic	15,296 (29)	6,691 (30)
Aspirin	6,789 (13)	2,931 (13)
Betablockers	8,638 (17)	3,883 (17)
Bicarbonate	2,046 (4)	654 (3)
Diuretics	5,544 (11)	2,246 (10)
Vasopressors or inotropes	10,778 (21)	4,925 (22)
Statin	4,586 (9)	2,199 (10)
Vancomycin	5,935 (11)	2,949 (13)
Nonsteroidal anti-inflammatory drugs	7,155 (14)	3,555 (16)
**Comorbidities within one year before admission date, n (%)**		
Charlson comorbidity index, median (IQR)	4 (2, 6)	4 (2, 7)
Alcohol or drug abuse	7,845 (15)	3,523 (16)
Myocardial Infarction	3,954 (8)	1,829 (8)
Congestive Heart Failure	8,433 (16)	4,153 (19)
Peripheral Vascular Disease	11,308 (22)	5,483 (25)
Cerebrovascular Disease	8,747 (17)	3,995 (18)

© 2022 Ren Y et al. *JAMA Network Open*.

Features	Development Cohort	Validation Cohort
Chronic Pulmonary Disease	16,269 (31)	7,431 (33)
Cancer	14,581 (28)	5,871 (26)
Metastatic Carcinoma	4,799 (9)	2,102 (9)
Liver Disease	7,651 (15)	3,529 (16)
Diabetes	12,554 (24)	5,463 (24)
Hypertension	32,477 (62)	14,786 (66)
Obesity	16,510 (32)	9,609 (43)
Fluid and electrolyte disorders	15,757 (30)	8,667 (39)
Valvular Disease	7,060 (14)	3,819 (17)
Coagulopathy	7,342 (14)	3,243 (15)
Weight Loss	7,272 (14)	3,583 (16)
Depression	14,462 (28)	6,677 (30)
Chronic anemia	10,884 (21)	6,106 (27)
Chronic Kidney Disease	10,138 (19)	4,840 (22)
End Stage Renal Disease	2,512 (5)	945 (4)
**Laboratory results information**		
Automated urinalysis, urine hemoglobin within 7 days prior to surgery, mg/dL, n (%)		
Missing	45,670 (88)	22,280 (100)
Negative	3,998 (8)	15 (0)
Small	1,271 (2)	1 (0)
Moderate	640 (1)	0 (0)
Large	538 (1)	4 (0)
Automated urinalysis, urine hemoglobin within 8-365 days prior to surgery, mg/dL, n (%)		
Missing	43,540 (84)	22,008 (99)
Negative	6,175 (12)	222 (1)
Small	1,040 (2)	41 (0)
Large	746 (1)	16 (0)
Moderate	616 (1)	13 (0)
Automated urinalysis, urine protein presence within 365 days prior to surgery, mg/dL, n (%)		
Missing	27,711 (53)	12,595 (56)
Negative	15,848 (30)	6,358 (29)
Moderate	5,632 (11)	2,518 (11)
Large	1,565 (3)	635 (3)
Small	1,361 (3)	194 (1)
Hemoglobin within 7 days prior to surgery, g/dl, median (IQR)		
Minimum	12.60 (10.70, 13.90)	12.45 (10.60, 13.80)
Maximum	13.10 (11.60, 14.40)	13.10 (11.50, 14.40)
Average	12.80 (11.14, 14.10)	12.75 (11.07, 14.00)

© 2022 Ren Y et al. *JAMA Network Open*.

Features	Development Cohort	Validation Cohort
Variance	0.44 (0.15, 1.11)	0.39 (0.13, 0.98)
Count	1 (0, 2)	1 (0, 2)
Hemoglobin within 8-365 days prior to surgery, g/dl, median (IQR)		
Minimum	11.90 (9.60, 13.60)	11.90 (9.50, 13.50)
Maximum	13.60 (12.40, 14.80)	13.60 (12.40, 14.70)
Average	12.60 (10.92, 13.90)	12.60 (10.90, 13.89)
Variance	0.91 (0.32, 1.86)	0.82 (0.31, 1.78)
Count	1 (0, 4)	1 (0, 4)
Glucose in blood, mg/dL, median (IQR)		
Minimum	100 (88, 119)	100 (89, 120)
Maximum	118 (98, 154)	119 (99, 155)
Average	110 (95, 135)	110 (96, 135)
Variance	8.33 (0.00, 280.33)	16.33 (0.00, 299.51)
Count	2 (0, 4)	2 (1, 4)
Urea nitrogen in blood, mg/dL, median (IQR)		
Minimum	14 (10, 19)	15 (11, 20)
Maximum	16 (12, 22)	17 (13, 23)
Average	15 (11, 21)	16 (12, 22)
Variance	0.00 (0.00, 3.00)	0.00 (0.00, 3.00)
Count	2 (0, 4)	2 (0, 4)
Serum creatinine, mg/dL, median (IQR)		
Minimum	0.85 (0.69, 1.08)	0.86 (0.69, 1.10)
Maximum	0.92 (0.74, 1.19)	0.93 (0.76, 1.19)
Average	0.89 (0.72, 1.12)	0.89 (0.73, 1.14)
Variance	0.00 (0.00, 0.00)	0.00 (0.00, 0.00)
Count	2 (0, 4)	2 (0, 4)
Serum Calcium, mmol/L, median (IQR)		
Minimum	9.10 (8.60, 9.60)	9.10 (8.60, 9.60)
Maximum	9.40 (9.00, 9.70)	9.40 (9.00, 9.70)
Average	9.21 (8.80, 9.60)	9.25 (8.80, 9.60)
Variance	0.00 (0.00, 0.05)	0.00 (0.00, 0.05)
Count	2 (0, 4)	2 (0, 4)
Serum Sodium, mmol/L, median (IQR)		
Minimum	138 (136, 140)	138 (135, 140)
Maximum	140 (138, 141)	139 (137, 141)
Average	139 (137, 141)	138 (136, 140)
Variance	2.45 (1.00, 6.25)	2.33 (0.92, 5.50)
Count	1 (0, 2)	1 (0, 2)
Urea nitrogen-Creatinine ratio, median (IQR)		
Minimum	15.19 (11.36, 19.72)	16.00 (12.00, 20.70)

© 2022 Ren Y et al. *JAMA Network Open*.

Features	Development Cohort	Validation Cohort
Maximum	17.20 (13.24, 22.35)	18.18 (14.00, 23.50)
Average	16.22 (12.50, 20.90)	17.13 (13.23, 21.96)
Variance	0.00 (0.00, 2.09)	0.00 (0.00, 2.25)
Count	4 (0, 8)	5 (0, 10)
Potassium in serum, mmol/L, median (IQR)		
Average	4.04 (3.80, 4.30)	4.00 (3.80, 4.30)
Count	1 (0, 2)	1 (0, 2)
Chloride in Serum, mmol/L, median (IQR)		
Maximum	102 (100, 105)	104 (101, 106)
Average	102 (99, 104)	103 (101, 105.25)
Variance	4.24 (1.48, 8.54)	3.50 (1.24, 8.00)
Count	1 (0, 2)	1 (0, 2)
Serum CO2, mmol/L, median (IQR)		
Minimum	24 (22, 26)	25 (22, 27)
Maximum	26 (24, 28)	26 (24, 28)
Average	25 (23, 27)	26 (23, 28)
Variance	2.86 (1.00, 6.33)	3.00 (1.07, 6.80)
Count	1 (0, 2)	1 (0, 2)
Anion gap in blood, mmol/L, median (IQR)		
Minimum	11 (9, 14)	9 (7, 11)
Average	12.33 (10.00, 15.00)	10.00 (8.00, 11.33)
Count	1 (0, 2)	1 (0, 2)
White Blood Cell in blood, thou/uL, median (IQR)		
Minimum	7.60 (5.90, 9.90)	7.50 (5.80, 9.80)
Maximum	8.70 (6.60, 12.00)	8.60 (6.50, 11.90)
Average	8.20 (6.40, 10.90)	8.03 (6.20, 10.75)
Variance	1.75 (0.50, 5.92)	1.51 (0.43, 4.94)
Count	1 (0, 2)	1 (0, 2)
Serum Red Blood Cell, Million/uL, median (IQR)		
Maximum	4.37 (3.92, 4.77)	4.33 (3.87, 4.75)
Average	4.29 (3.79, 4.69)	4.24 (3.74, 4.67)
Hematocrit in blood, %, median (IQR)		
Minimum	37.90 (32.70, 41.70)	37.20 (32.00, 41.10)
Maximum	39.50 (35.30, 43.00)	39.00 (34.70, 42.50)
Average	38.70 (34.10, 42.20)	38.03 (33.40, 41.70)
Variance	0.00 (0.00, 2.43)	0.00 (0.00, 2.43)
Count	2 (0, 4)	2 (0, 4)
The amount of hemoglobin relative to the size of the cell in blood, g/dL, median (IQR)		
Maximum	33.60 (32.70, 34.30)	33.70 (33.00, 34.30)
Average	33.25 (32.30, 34.00)	33.50 (32.87, 34.10)

© 2022 Ren Y et al. *JAMA Network Open*.

Features	Development Cohort	Validation Cohort
Red cell distribution width in Blood, %, median (IQR)		
Minimum	14.10 (13.30, 15.30)	14.10 (13.40, 15.50)
Maximum	14.40 (13.50, 15.60)	14.30 (13.50, 15.80)
Average	14.30 (13.45, 15.46)	14.20 (13.40, 15.60)
Variance	0.08 (0.02, 0.28)	0.04 (0.02, 0.09)
Platelet in blood, thou/uL, median (IQR)		
Minimum	222 (174, 277)	228 (179, 286)
Maximum	239 (191, 299)	246 (196, 308)
Average	230 (183.33, 287.00)	236 (188.00, 295.00)
Variance	412.92 (120.75, 1152.00)	372.17 (112.50, 1036.72)
Mean platelet volume, fL, median (IQR)		
Minimum	8.00 (7.40, 8.80)	8.20 (7.60, 9.00)
Average	8.20 (7.54, 8.94)	8.39 (7.70, 9.10)
Automated urinalysis, urine glucose within 7 days prior to surgery, mg/dL, median (IQR)		
Missing	39,924 (77)	17,429 (78)
Negative	10,730 (21)	4,247 (19)
Small	811 (2)	328 (1)
Moderate	396 (1)	274 (1)
Large	256 (0)	22 (0)
Automated urinalysis, urine glucose within 8-365 days prior to surgery, mg/dL, median (IQR)		
Missing	35,982 (69)	16,028 (72)
Negative	14,813 (28)	5,692 (26)
Small	570 (1)	236 (1)
Large	387 (1)	65 (0)
Moderate	365 (1)	279 (1)
Average of Mean Corpuscular Volume in blood, fL, median (IQR)	90.28 (86.30, 94.10)	89.50 (85.50, 93.35)
Number of urine glucose tests within 0-7 days prior to surgery, median (IQR)	0 (0, 0)	0 (0, 0)
Number of urine glucose tests within 8-365 days prior to surgery, median (IQR)	0 (0, 1)	0 (0, 1)
Number of urine hemoglobin tests within 8-365 days prior to surgery, median (IQR)	0 (0, 0)	0 (0, 0)
Count of the amount of hemoglobin relative to the size of the cell in blood test, median (IQR)	2 (0, 2)	1 (0, 2)
Reference estimated glomerular filtration rate, median (IQR)	94.04 (78.53, 109.34)	92.46 (77.62, 107.26)

Abbreviations: SD, standard deviation; IQR, interquartile range; OB, obstetrician; ACE, Angiotensin-converting enzyme.
[a]Data is reported based on values calculated at the latest hospital admission.
[b]Race and ethnicity are self-reported.
[c]Other race includes American Indian/Alaskan Native, Asian, Native Hawaiian/other Pacific Islander, and multiracial.
[d]Other surgery type includes plastic surgery, burn surgery, pediatric surgery, transplantation, ophthalmology, medicine gastroenterology, and interventional cardiology.

© 2022 Ren Y et al. *JAMA Network Open*.

eTable 3. Generalized Additive Model Performance Measurements for Complications With 95% CIs Calculated by 1000 Bootstrap Samples on the Validation Cohort

Complication	Input features (n)	Sensitivity	Specificity	PPV	NPV	Accuracy	AUROC	AUPRC	Threshold
Cardiovascular complication	55	0.7 (0.69, 0.71)	0.75 (0.73, 0.76)	0.33 (0.32, 0.34)	0.93 (0.93, 0.94)	0.71 (0.7, 0.71)	0.8 (0.79, 0.8)	0.45 (0.43, 0.46)	0.13
	101	0.67 (0.66, 0.68)	0.75 (0.74, 0.76)	0.31 (0.3, 0.32)	0.93 (0.93, 0.94)	0.68 (0.68, 0.69)	0.78 (0.77, 0.79)	0.44 (0.42, 0.46)	0.11
	135	0.71 (0.71, 0.72)	0.75 (0.74, 0.77)	0.34 (0.33, 0.35)	0.94 (0.93, 0.94)	0.72 (0.71, 0.72)	0.81 (0.8, 0.82)	0.48 (0.47, 0.5)	0.12
Prolonged ICU stay	55	0.74 (0.73, 0.74)	0.81 (0.8, 0.82)	0.55 (0.54, 0.56)	0.91 (0.9, 0.91)	0.76 (0.75, 0.76)	0.86 (0.86, 0.87)	0.74 (0.72, 0.75)	0.2
	101	0.75 (0.75, 0.76)	0.77 (0.76, 0.78)	0.55 (0.54, 0.56)	0.89 (0.89, 0.9)	0.76 (0.75, 0.76)	0.85 (0.84, 0.86)	0.71 (0.7, 0.73)	0.17
	135	0.79 (0.79, 0.8)	0.78 (0.77, 0.79)	0.6 (0.59, 0.61)	0.9 (0.89, 0.9)	0.79 (0.78, 0.79)	0.88 (0.87, 0.88)	0.77 (0.76, 0.78)	0.19
Neurological complication and delirium	55	0.79 (0.78, 0.79)	0.77 (0.75, 0.78)	0.39 (0.38, 0.4)	0.95 (0.95, 0.95)	0.78 (0.78, 0.79)	0.85 (0.85, 0.86)	0.57 (0.55, 0.59)	0.12
	101	0.72 (0.71, 0.72)	0.78 (0.77, 0.8)	0.33 (0.32, 0.34)	0.95 (0.95, 0.95)	0.73 (0.72, 0.73)	0.83 (0.82, 0.84)	0.5 (0.48, 0.52)	0.1
	135	0.77 (0.77, 0.78)	0.79 (0.78, 0.8)	0.38 (0.37, 0.39)	0.95 (0.95, 0.96)	0.77 (0.77, 0.78)	0.86 (0.86, 0.87)	0.58 (0.57, 0.6)	0.1
Wound complication	55	0.69 (0.68, 0.69)	0.7 (0.69, 0.72)	0.38 (0.37, 0.39)	0.89 (0.89, 0.9)	0.69 (0.68, 0.7)	0.77 (0.76, 0.77)	0.52 (0.51, 0.54)	0.16
	101	0.64 (0.63, 0.64)	0.65 (0.64, 0.66)	0.33 (0.32, 0.34)	0.87 (0.86, 0.87)	0.64 (0.63, 0.65)	0.69 (0.68, 0.7)	0.4 (0.39, 0.42)	0.16
	135	0.71 (0.71, 0.72)	0.69 (0.68, 0.7)	0.4 (0.39, 0.41)	0.89 (0.89, 0.9)	0.71 (0.7, 0.72)	0.77 (0.77, 0.78)	0.53 (0.52, 0.55)	0.17
Sepsis	55	0.72 (0.72, 0.73)	0.8 (0.78, 0.81)	0.22 (0.21, 0.23)	0.97 (0.97, 0.98)	0.73 (0.72, 0.73)	0.84 (0.83, 0.84)	0.36 (0.34, 0.38)	0.07
	101	0.83 (0.82, 0.83)	0.69 (0.67, 0.71)	0.28 (0.27, 0.29)	0.97 (0.96, 0.97)	0.82 (0.81, 0.82)	0.84 (0.83, 0.85)	0.41 (0.39, 0.43)	0.09
	135	0.74 (0.74, 0.75)	0.8 (0.79, 0.82)	0.23 (0.22, 0.24)	0.98 (0.97, 0.98)	0.75 (0.74, 0.75)	0.86 (0.85, 0.86)	0.43 (0.4, 0.45)	0.06
Venous thromboembolism	55	0.66 (0.66, 0.67)	0.8 (0.78, 0.82)	0.12 (0.12, 0.13)	0.98 (0.98, 0.98)	0.67 (0.66, 0.68)	0.8 (0.79, 0.81)	0.23 (0.21, 0.25)	0.04

© 2022 Ren Y et al. *JAMA Network Open*.

Complication	Input features (n)	Sensitivity	Specificity	PPV	NPV	Accuracy	AUROC	AUPRC	Threshold
	101	0.67 (0.67, 0.68)	0.77 (0.74, 0.79)	0.12 (0.12, 0.13)	0.98 (0.98, 0.98)	0.68 (0.67, 0.69)	0.78 (0.77, 0.79)	0.2 (0.18, 0.22)	0.04
	135	0.75 (0.74, 0.76)	0.74 (0.72, 0.76)	0.15 (0.14, 0.16)	0.98 (0.98, 0.98)	0.75 (0.74, 0.75)	0.81 (0.8, 0.83)	0.24 (0.22, 0.27)	0.05
Prolonged mechanical ventilation	55	0.79 (0.78, 0.79)	0.84 (0.82, 0.86)	0.19 (0.18, 0.2)	0.99 (0.99, 0.99)	0.79 (0.78, 0.79)	0.89 (0.88, 0.9)	0.41 (0.38, 0.44)	0.05
	101	0.8 (0.8, 0.81)	0.76 (0.74, 0.79)	0.19 (0.18, 0.2)	0.98 (0.98, 0.98)	0.8 (0.8, 0.81)	0.87 (0.86, 0.88)	0.39 (0.36, 0.42)	0.04
	135	0.84 (0.84, 0.85)	0.8 (0.78, 0.82)	0.23 (0.22, 0.24)	0.99 (0.98, 0.99)	0.84 (0.84, 0.85)	0.91 (0.9, 0.91)	0.47 (0.44, 0.5)	0.05
Acute kidney injury	55	0.73 (0.72, 0.73)	0.72 (0.71, 0.74)	0.34 (0.33, 0.35)	0.93 (0.93, 0.93)	0.72 (0.72, 0.73)	0.8 (0.79, 0.8)	0.46 (0.44, 0.48)	0.15
	101	0.71 (0.71, 0.72)	0.72 (0.71, 0.74)	0.33 (0.32, 0.34)	0.93 (0.93, 0.93)	0.71 (0.71, 0.72)	0.79 (0.78, 0.79)	0.45 (0.44, 0.47)	0.13
	135	0.69 (0.68, 0.7)	0.79 (0.78, 0.8)	0.33 (0.32, 0.35)	0.94 (0.94, 0.95)	0.71 (0.7, 0.71)	0.82 (0.81, 0.83)	0.51 (0.49, 0.53)	0.12
30-day mortality	55	0.69 (0.69, 0.7)	0.83 (0.79, 0.87)	0.05 (0.04, 0.05)	1.0 (0.99, 1.0)	0.69 (0.69, 0.7)	0.84 (0.82, 0.86)	0.1 (0.08, 0.12)	0.06
	101	0.68 (0.67, 0.68)	0.81 (0.77, 0.85)	0.04 (0.04, 0.05)	0.99 (0.99, 1.0)	0.68 (0.67, 0.69)	0.82 (0.8, 0.84)	0.1 (0.08, 0.12)	0.05
	135	0.73 (0.73, 0.74)	0.81 (0.77, 0.85)	0.05 (0.05, 0.06)	1.0 (0.99, 1.0)	0.73 (0.73, 0.74)	0.84 (0.82, 0.86)	0.12 (0.1, 0.15)	0.06
90-day mortality	55	0.72 (0.71, 0.73)	0.79 (0.76, 0.82)	0.08 (0.07, 0.08)	0.99 (0.99, 0.99)	0.72 (0.72, 0.73)	0.82 (0.81, 0.84)	0.14 (0.11, 0.16)	0.12
	101	0.67 (0.67, 0.68)	0.81 (0.78, 0.84)	0.07 (0.06, 0.07)	0.99 (0.99, 0.99)	0.68 (0.67, 0.68)	0.81 (0.8, 0.83)	0.13 (0.11, 0.15)	0.1
	135	0.77 (0.76, 0.77)	0.73 (0.69, 0.76)	0.08 (0.07, 0.09)	0.99 (0.99, 0.99)	0.76 (0.76, 0.77)	0.82 (0.8, 0.84)	0.14 (0.12, 0.16)	0.13

Abbreviations: PPV, positive predictive value; NPV, negative predictive value; AUROC, area under the receiver operating characteristic curve; AUPRC, area under the precision-recall curve; ICU, intensive care unit.

© 2022 Ren Y et al. *JAMA Network Open*.

eTable 4. Summary of the Contributions of Each Input Feature to Each Outcome for a Generalized Additive Model With 55 Input Features

Features	Cardiovascular complication	Prolonged ICU stay	Neurological complication and delirium	Wound complication	Sepsis	Venous thromboembolism	Prolonged mechanical ventilation	Acute kidney injury
	**Odds ratio (99% confidence interval)**							
Indicator of receiving ACE Inhibitors within one year before admission date	1.04 (0.9, 1.22)	1.05 (0.91, 1.22)	0.98 (0.82, 1.17)	0.98 (0.86, 1.12)	1.0 (0.83, 1.2)	0.98 (0.78, 1.23)	1.01 (0.78, 1.32)	1.05 (0.91, 1.22)
Admission Source (Transfer vs other)	1.33 (1.19, 1.5)*	1.8 (1.61, 2.01)*	1.42 (1.25, 1.61)*	1.07 (0.96, 1.2)	1.45 (1.27, 1.64)*	1.36 (1.16, 1.59)*	1.58 (1.34, 1.87)*	1.35 (1.21, 1.52)*
Alcohol abuse or drug	1.06 (0.95, 1.19)	1.09 (0.98, 1.21)	1.18 (1.04, 1.34)*	1.04 (0.94, 1.16)	1.06 (0.93, 1.21)	0.97 (0.83, 1.15)	1.06 (0.9, 1.26)	1.11 (1.0, 1.24)
Indicator of receiving Aminoglycosides within one year before admission date	1.04 (0.83, 1.3)	1.12 (0.9, 1.39)	0.95 (0.71, 1.28)	1.15 (0.96, 1.39)	1.12 (0.88, 1.42)	1.18 (0.87, 1.59)	0.96 (0.63, 1.45)	1.14 (0.94, 1.39)
Chronic anemia	1.09 (0.99, 1.21)	0.96 (0.86, 1.06)	1.02 (0.9, 1.16)	1.24 (1.13, 1.36)*	1.13 (0.99, 1.28)	1.26 (1.08, 1.46)*	0.91 (0.77, 1.08)	1.13 (1.02, 1.25)*
Indicator of receiving Antiemetic within one year before admission date	0.92 (0.81, 1.05)	0.91 (0.81, 1.02)	0.85 (0.73, 0.99)*	1.2 (1.07, 1.35)*	0.94 (0.8, 1.11)	0.99 (0.81, 1.2)	0.86 (0.7, 1.07)	0.96 (0.85, 1.08)
Indicator of receiving Aspirin within one year before admission date	0.95 (0.83, 1.1)	0.79 (0.69, 0.91)*	0.93 (0.79, 1.11)	1.02 (0.9, 1.16)	0.99 (0.83, 1.19)	0.93 (0.75, 1.15)	0.79 (0.62, 1.01)	0.9 (0.78, 1.03)
Indicator of receiving Beta-blockers within one year before admission date	0.83 (0.72, 0.96)*	1.0 (0.87, 1.14)	0.87 (0.73, 1.03)	1.15 (1.02, 1.3)*	0.91 (0.77, 1.09)	1.05 (0.85, 1.31)	0.85 (0.67, 1.09)	1.11 (0.97, 1.27)
Indicator of receiving Bicarbonate within one year before admission date	0.88 (0.73, 1.06)	0.81 (0.67, 0.98)*	0.81 (0.64, 1.02)	0.85 (0.72, 1.01)	0.82 (0.66, 1.03)	1.07 (0.82, 1.39)	0.67 (0.48, 0.94)*	0.87 (0.71, 1.05)
Coagulopathy	1.23 (1.1, 1.37)*	1.07 (0.96, 1.19)	1.33 (1.17, 1.51)*	1.17 (1.06, 1.3)*	1.25 (1.09, 1.42)*	1.59 (1.36, 1.85)*	1.42 (1.19, 1.68)*	1.21 (1.09, 1.35)*
Diabetes	0.86 (0.78, 0.95)*	0.96 (0.87, 1.05)	0.85 (0.76, 0.96)*	0.9 (0.81, 0.99)*	0.99 (0.87, 1.13)	0.85 (0.72, 0.99)*	0.87 (0.74, 1.03)	1.01 (0.91, 1.11)

© 2022 Ren Y et al. *JAMA Network Open*.

Features	Cardiovascular complication	Prolonged ICU stay	Neurological complication and delirium	Wound complication	Sepsis	Venous thromboembolism	Prolonged mechanical ventilation	Acute kidney injury
Indicator of receiving Diuretics within one year before admission date	1.07 (0.93, 1.24)	1.11 (0.96, 1.28)	0.98 (0.82, 1.18)	0.88 (0.77, 1.0)	1.03 (0.86, 1.23)	0.9 (0.72, 1.12)	1.36 (1.05, 1.75)*	1.19 (1.03, 1.37)*
Emergent or elective admission	1.22 (1.09, 1.36)*	1.24 (1.12, 1.38)*	0.97 (0.86, 1.1)	0.82 (0.74, 0.9)*	0.75 (0.66, 0.85)*	0.81 (0.7, 0.95)*	1.35 (1.14, 1.58)*	1.05 (0.94, 1.17)
Hypertension	1.05 (0.94, 1.16)	1.32 (1.2, 1.45)*	1.24 (1.11, 1.4)*	1.09 (0.99, 1.2)	1.13 (0.99, 1.29)	1.21 (1.03, 1.41)*	1.33 (1.13, 1.57)*	1.4 (1.26, 1.54)*
Cancer	1.04 (0.92, 1.18)	1.13 (1.01, 1.27)*	0.84 (0.73, 0.97)*	0.97 (0.86, 1.09)	1.03 (0.88, 1.21)	1.05 (0.87, 1.27)	0.86 (0.7, 1.06)	1.01 (0.9, 1.14)
Congestive Heart Failure	1.47 (1.32, 1.64)*	1.27 (1.13, 1.42)*	1.2 (1.05, 1.37)*	1.07 (0.96, 1.2)	1.24 (1.08, 1.43)*	1.08 (0.91, 1.27)	1.46 (1.23, 1.73)*	1.36 (1.22, 1.52)*
Chronic Pulmonary Disease	1.06 (0.97, 1.16)	1.05 (0.96, 1.14)	0.96 (0.87, 1.07)	1.03 (0.94, 1.12)	0.94 (0.84, 1.05)	0.9 (0.79, 1.04)	1.12 (0.97, 1.29)	0.96 (0.88, 1.05)
Cerebrovascular Disease	1.03 (0.93, 1.14)	1.23 (1.12, 1.36)*	3.09 (2.77, 3.45)*	0.91 (0.82, 1.01)	0.91 (0.8, 1.05)	1.22 (1.06, 1.42)*	1.53 (1.31, 1.78)*	1.03 (0.93, 1.14)
Myocardial Infarction	1.14 (1.0, 1.31)	1.03 (0.89, 1.18)	0.98 (0.83, 1.15)	0.99 (0.87, 1.14)	1.03 (0.87, 1.23)	1.12 (0.91, 1.38)	1.22 (0.98, 1.52)	0.94 (0.82, 1.08)
Peripheral Vascular Disease	1.08 (0.98, 1.2)	0.99 (0.89, 1.09)	0.89 (0.79, 1.01)	1.07 (0.96, 1.19)	1.05 (0.92, 1.2)	0.93 (0.79, 1.09)	0.94 (0.81, 1.1)	1.01 (0.91, 1.12)
Liver Disease	1.09 (0.98, 1.23)	0.91 (0.81, 1.02)	0.92 (0.79, 1.06)	0.86 (0.77, 0.96)*	1.02 (0.89, 1.17)	0.94 (0.79, 1.12)	1.01 (0.83, 1.22)	0.99 (0.88, 1.1)
Nonsteroidal anti-inflammatory drugs	0.98 (0.85, 1.12)	0.93 (0.82, 1.06)	1.05 (0.9, 1.24)	1.14 (1.02, 1.28)*	1.14 (0.97, 1.33)	1.01 (0.82, 1.23)	1.0 (0.79, 1.26)	1.03 (0.91, 1.17)
Indicator of receiving pressors or inotropes within one year before admission date	1.0 (0.88, 1.15)	0.83 (0.73, 0.94)*	0.86 (0.73, 1.02)	1.36 (1.21, 1.53)*	0.89 (0.76, 1.05)	0.93 (0.76, 1.14)	0.79 (0.63, 1.0)*	0.83 (0.73, 0.95)*
Sex (Male vs female)	0.93 (0.85, 1.01)	1.08 (1.0, 1.17)	1.14 (1.04, 1.26)*	1.07 (0.99, 1.16)	1.2 (1.08, 1.33)*	1.06 (0.94, 1.2)	1.05 (0.92, 1.2)	1.04 (0.96, 1.13)
Indicator of receiving statin within one year before admission date	0.93 (0.8, 1.09)	0.97 (0.83, 1.13)	1.11 (0.91, 1.34)	1.01 (0.87, 1.16)	0.88 (0.72, 1.07)	0.82 (0.64, 1.05)	0.77 (0.58, 1.02)	0.88 (0.76, 1.03)

© 2022 Ren Y et al. *JAMA Network Open*.

Features	Cardiovascular complication	Prolonged ICU stay	Neurological complication and delirium	Wound complication	Sepsis	Venous thromboembolism	Prolonged mechanical ventilation	Acute kidney injury
Valvular Disease	1.09 (0.97, 1.22)	1.04 (0.93, 1.17)	0.84 (0.73, 0.96)*	1.08 (0.97, 1.2)	1.04 (0.9, 1.2)	0.98 (0.83, 1.16)	0.81 (0.68, 0.97)*	1.0 (0.89, 1.12)
Indicator of receiving Vancomycin within one year before admission date	1.0 (0.86, 1.16)	0.86 (0.74, 0.99)*	0.86 (0.72, 1.03)	1.11 (0.98, 1.25)	1.3 (1.09, 1.54)*	1.09 (0.88, 1.34)	0.9 (0.7, 1.16)	1.08 (0.94, 1.24)
Weight Loss	1.72 (1.56, 1.9)*	2.04 (1.85, 2.25)*	1.67 (1.48, 1.88)*	1.35 (1.23, 1.49)*	1.71 (1.52, 1.92)*	1.36 (1.17, 1.58)*	2.21 (1.9, 2.56)*	1.23 (1.11, 1.36)*
	**Degrees of freedom (p value)**							
Admission Day	1.5098 (0.18)	1.0001 (<0.001)*	1.0 (<0.001)*	1.0002 (0.19)	1.0008 (0.07)	1.0321 (0.02)	1.0001 (0.01)*	1.0001 (0.89)
Admission Month	1.0001 (<0.001)*	1.0002 (<0.001)*	1.2524 (<0.001)*	1.0001 (<0.001)*	1.0002 (0.003)*	1.0002 (0.08)	1.0002 (<0.001)*	1.0002 (<0.001)*
Medicine or surgery admitting	1.8424 (<0.001)*	1.854 (<0.001)*	1.9367 (<0.001)*	1.9006 (0.002)*	1.0003 (<0.001)*	1.0001 (0.92)	1.9282 (<0.001)*	1.0001 (0.66)
Age	3.3289 (<0.001)*	2.8822 (<0.001)*	1.0001 (<0.001)*	2.5977 (<0.001)*	2.787 (<0.001)*	1.0844 (0.56)	2.9891 (0.009)*	2.9349 (<0.001)*
ID of Attending Surgeon	1.5801 (<0.001)*	3.4827 (<0.001)*	3.7365 (<0.001)*	1.3691 (<0.001)*	2.8631 (<0.001)*	3.0598 (<0.001)*	2.2472 (<0.001)*	2.1625 (<0.001)*
Charlson comorbidity index	2.5768 (0.005)*	2.4877 (0.03)	3.7929 (0.002)*	2.7247 (0.001)*	2.5768 (0.004)*	2.3515 (0.22)	2.2234 (0.33)	3.0969 (<0.001)*
Number of hemoglobin tests within 7 days prior to surgery	3.8239 (<0.001)*	3.2297 (<0.001)*	3.529 (<0.001)*	1.0001 (0.03)	3.8641 (<0.001)*	3.7024 (<0.001)*	3.1826 (<0.001)*	3.7891 (<0.001)*
County of home address	3.0863 (0.11)	1.3308 (0.05)	1.0001 (0.46)	1.0001 (0.79)	2.7571 (0.13)	1.4373 (0.22)	1.0002 (0.24)	1.0001 (0.03)
Distance of residence to hospital, km	1.88 (0.002)*	1.0009 (0.46)	1.7502 (<0.001)*	1.0003 (0.006)*	1.0017 (<0.001)*	1.0003 (<0.001)*	1.0001 (<0.001)*	1.7305 (0.10)
Reference estimated glomerular filtration rate	3.0924 (<0.001)*	3.8893 (<0.001)*	3.3378 (<0.001)*	1.0002 (0.09)	3.3507 (0.004)*	1.0001 (<0.001)*	3.4254 (<0.001)*	3.7513 (<0.001)*
Automated urinalysis, urine glucose within 7 days prior to surgery, mg/dL	1.0002 (0.02)	1.9877 (<0.001)*	1.0 (<0.001)*	1.0001 (0.02)	1.0001 (<0.001)*	1.6744 (0.29)	1.9581 (<0.001)*	1.9844 (<0.001)*

© 2022 Ren Y et al. *JAMA Network Open*.

Features	Cardiovascular complication	Prolonged ICU stay	Neurological complication and delirium	Wound complication	Sepsis	Venous thromboembolism	Prolonged mechanical ventilation	Acute kidney injury
Min hemoglobin within 7 days prior to surgery, g/dl	2.0092 (<0.001)*	3.206 (<0.001)*	1.0003 (<0.001)*	2.4843 (<0.001)*	3.5175 (<0.001)*	1.2745 (<0.001)*	3.3699 (<0.001)*	3.588 (<0.001)*
Automated urinalysis, urine hemoglobin within 7 days prior to surgery, mg/dL	2.6552 (<0.001)*	1.0003 (0.002)*	2.3985 (<0.001)*	1.848 (<0.001)*	2.6174 (<0.001)*	2.1791 (0.07)	1.7991 (0.002)*	1.9838 (0.06)
Median total income at patient residential area, USD	1.241 (0.36)	1.0001 (0.84)	1.0001 (0.82)	1.0001 (0.10)	1.0009 (0.26)	1.0002 (0.82)	1.0001 (0.77)	1.2457 (0.85)
Insurance paying the bills	1.8572 (<0.001)*	1.951 (<0.001)*	1.0001 (0.37)	1.7663 (<0.001)*	1.0002 (<0.001)*	1.0002 (0.84)	1.0001 (0.006)*	1.8354 (0.002)*
Prevalence of residents living below poverty at patient residential area, %	1.0002 (0.62)	2.8412 (0.02)	1.0001 (0.63)	1.0001 (0.62)	1.001 (0.18)	1.6562 (0.71)	1.0001 (0.72)	1.001 (0.74)
Current Procedural Terminology code of the primary procedure	3.1802 (<0.001)*	3.4318 (<0.001)*	1.0002 (<0.001)*	3.7095 (<0.001)*	3.1177 (<0.001)*	2.7864 (<0.001)*	2.0574 (<0.001)*	3.7337 (<0.001)*
Prevalence of African American residents living below poverty at patient residential area, %	1.0002 (0.71)	1.0001 (0.28)	1.0001 (0.15)	1.7377 (0.58)	1.0004 (0.64)	1.0002 (0.02)	1.0001 (0.88)	2.2278 (0.05)
Prevalence of Hispanic residents living below poverty at patient residential area, %	2.8157 (0.16)	1.0002 (0.81)	2.7882 (0.19)	1.6307 (0.58)	1.761 (0.41)	1.0004 (0.80)	2.6446 (0.40)	2.8967 (0.14)
Race	1.6459 (0.15)	1.2861 (0.76)	1.69 (0.04)	1.0001 (0.29)	1.6151 (0.06)	1.0001 (0.12)	1.1101 (0.006)*	1.0002 (0.70)
Rural or city at patient residential area	1.0001 (0.32)	1.0003 (0.83)	1.0001 (0.24)	1.5005 (0.43)	1.7185 (0.23)	1.0154 (0.18)	1.0001 (0.98)	1.0001 (0.49)
Smoking Status	1.9581 (<0.001)*	1.9164 (<0.001)*	1.0001 (<0.001)*	1.8188 (0.09)	1.0022 (0.04)	1.8053 (0.10)	1.0001 (<0.001)*	1.7064 (0.04)
Surgery Type	3.5907 (<0.001)*	3.7322 (<0.001)*	3.6761 (<0.001)*	3.554 (<0.001)*	3.6889 (<0.001)*	1.8742 (0.36)	3.7307 (<0.001)*	3.198 (<0.001)*
Time from Admission to Surgery, days	3.9306 (<0.001)*	3.9494 (<0.001)*	3.6413 (<0.001)*	2.2518 (0.17)	3.878 (<0.001)*	1.0002 (0.02)	3.9659 (<0.001)*	3.7195 (0.002)*

© 2022 Ren Y et al. *JAMA Network Open*.

Features	Cardiovascular complication	Prolonged ICU stay	Neurological complication and delirium	Wound complication	Sepsis	Venous thromboembolism	Prolonged mechanical ventilation	Acute kidney injury
Total population at patient residential area	1.0002 (0.42)	1.0001 (0.05)	1.0001 (0.81)	1.6019 (0.69)	1.0002 (0.22)	1.0002 (0.24)	1.0001 (0.72)	1.6555 (0.54)
Automated urinalysis, urine protein presence within 365 days prior to surgery, mg/dL	2.0877 (0.006)*	2.9889 (<0.001)*	1.0019 (0.26)	3.5415 (<0.001)*	2.2968 (<0.001)*	1.0001 (0.47)	2.4826 (<0.001)*	3.0263 (<0.001)*
ZIP Code of home address	2.0425 (<0.001)*	2.697 (<0.001)*	2.3877 (<0.001)*	2.2397 (<0.001)*	3.3912 (<0.001)*	2.6053 (<0.001)*	3.4707 (<0.001)*	1.7438 (<0.001)*

*Statistically significant feature for the model
Abbreviations: ICU, intensive care unit; ACE, Angiotensin-converting enzyme.

© 2022 Ren Y et al. *JAMA Network Open*.

eTable 5. Summary of the Contributions of Each Input Feature to Each Outcome for a Generalized Additive Model With 101 Input Features

Feature	Cardiovascular complication	Prolonged ICU stay	Neurological complication and delirium	Wound complication	Sepsis	Venous thromboembolism	Prolonged mechanical ventilation	Acute kidney injury
	Odds ratio (99% Confidence Interval)							
Indicator of receiving ACE Inhibitors within one year before admission date	0.96 (0.83, 1.11)	0.92 (0.8, 1.06)	1.06 (0.91, 1.24)	1.03 (0.92, 1.16)	0.94 (0.78, 1.12)	0.96 (0.77, 1.2)		1.01 (0.87, 1.16)
Admission Source (Transfer vs other)	1.23 (1.09, 1.38)*	1.58 (1.4, 1.77)*	1.49 (1.33, 1.66)*	1.05 (0.94, 1.17)	1.33 (1.17, 1.52)*	1.22 (1.04, 1.42)*	1.45 (1.23, 1.71)*	1.38 (1.24, 1.54)*
Anesthesia Type (Local vs other)	0.93 (0.79, 1.08)	0.69 (0.59, 0.8)*	0.73 (0.6, 0.87)*	0.55 (0.48, 0.63)*	0.73 (0.58, 0.92)*		0.55 (0.38, 0.78)*	0.85 (0.73, 1.0)*
Indicator of receiving Antiemetic within one year before admission date	1.04 (0.94, 1.16)	1.06 (0.94, 1.19)	0.89 (0.79, 1.01)	1.35 (1.21, 1.5)*	1.13 (0.98, 1.31)	0.98 (0.83, 1.17)	1.0 (0.81, 1.22)	1.0 (0.89, 1.13)
Indicator of receiving Beta-blockers within one year before admission date	0.89 (0.78, 1.02)	1.03 (0.91, 1.17)		1.15 (1.03, 1.29)*	0.94 (0.8, 1.11)	0.99 (0.81, 1.22)		1.15 (1.01, 1.32)*
Indicator of receiving Bicarbonate within one year before admission date	0.87 (0.72, 1.05)		0.8 (0.63, 1.01)		0.74 (0.58, 0.93)*	0.97 (0.74, 1.26)		0.71 (0.58, 0.87)*
Indicator of receiving Diuretics within one year before admission date	1.07 (0.92, 1.24)							1.1 (0.95, 1.27)
Emergent or elective admission	0.98 (0.88, 1.1)	1.01 (0.9, 1.12)		0.68 (0.62, 0.76)*	0.68 (0.59, 0.79)*	0.6 (0.51, 0.71)*	0.93 (0.79, 1.1)	
Yes if the admission we happened at night	1.07 (0.99, 1.16)	1.29 (1.19, 1.38)*		0.89 (0.83, 0.95)*				
Scheduled post operation location	0.49 (0.45, 0.55)*	0.18 (0.16, 0.19)*	0.49 (0.44, 0.54)*	0.82 (0.74, 0.89)*	0.56 (0.49, 0.63)*	0.67 (0.58, 0.77)*	0.32 (0.28, 0.37)*	0.67 (0.61, 0.74)*
Scheduled room is trauma room or not	2.14 (1.42, 3.21)*		0.95 (0.64, 1.4)	2.18 (1.5, 3.17)*	1.08 (0.7, 1.69)	2.23 (1.41, 3.54)*	1.39 (0.92, 2.12)	1.46 (1.0, 2.12)*

© 2022 Ren Y et al. *JAMA Network Open*.

Feature	Cardiovascular complication	Prolonged ICU stay	Neurological complication and delirium	Wound complication	Sepsis	Venous thromboembolism	Prolonged mechanical ventilation	Acute kidney injury
Sex (Male vs female)	0.88 (0.81, 0.96)*	1.09 (1.01, 1.18)*		1.1 (1.02, 1.18)*	1.27 (1.14, 1.42)*			0.97 (0.89, 1.05)
Indicator of receiving pressors or inotropes within one year before admission date		0.84 (0.74, 0.96)*		1.49 (1.33, 1.66)*			0.88 (0.7, 1.11)	0.86 (0.76, 0.98)*
Indicator of receiving statin within one year before admission date						0.73 (0.58, 0.91)*	0.75 (0.59, 0.94)*	0.8 (0.69, 0.93)*
Age							1.69 (1.11, 2.58)*	
CKD status at admission								0.9 (0.81, 1.0)
	**Degrees of freedom (p value)**							
Admission Month	1.0002 (<0.001)*	1.0003 (0.003)*	1.2746 (0.03)	1.0001 (<0.001)*	1.0 (<0.001)*		1.0065 (0.08)	1.0001 (0.02)
Medicine or surgery admitting	1.8331 (0.008)*	1.7124 (<0.001)*	1.9627 (<0.001)*	1.6129 (<0.001)*	1.9679 (<0.001)*	1.0001 (<0.001)*	1.7777 (0.03)	1.9472 (<0.001)*
Age	3.0182 (<0.001)*	2.7487 (<0.001)*	1.0 (<0.001)*	2.6402 (<0.001)*	2.6581 (0.003)*		3.1255 (<0.001)*	2.3614 (<0.001)*
ID of Attending Surgeon	1.0003 (<0.001)*	3.6195 (<0.001)*	1.4528 (<0.001)*	2.4855 (<0.001)*	1.0 (<0.001)*	2.9976 (<0.001)*	3.4071 (<0.001)*	1.0008 (<0.001)*
Body Mass Index	3.4186 (<0.001)*	3.4922 (<0.001)*	2.6968 (<0.001)*				3.4427 (<0.001)*	3.4896 (<0.001)*
Average of Urea nitrogen in blood, mg/dL	3.3713 (0.003)*	3.6002 (0.03)	1.8925 (0.12)	1.0001 (0.12)	1.0 (0.25)	1.0002 (0.95)	1.0002 (0.96)	1.0001 (0.81)
Max of Urea nitrogen in blood, mg/dL	1.0002 (0.02)	1.001 (0.77)	1.0 (0.31)	1.0004 (0.30)	1.5392 (0.02)	1.0001 (0.44)	1.0005 (0.03)	3.3817 (<0.001)*
Min of Urea nitrogen in blood, mg/dL	1.0001 (0.49)		1.0 (0.13)		2.5039 (0.26)	1.2037 (0.84)	1.0002 (0.02)	2.9518 (<0.001)*
Variance of Urea nitrogen in blood, mg/dL	1.0003 (0.18)	1.0003 (0.05)	1.6409 (0.56)		1.9607 (0.06)		2.2467 (0.05)	1.0 (0.92)

© 2022 Ren Y et al. *JAMA Network Open*.

Feature	Cardiovascular complication	Prolonged ICU stay	Neurological complication and delirium	Wound complication	Sepsis	Venous thromboembolism	Prolonged mechanical ventilation	Acute kidney injury
Charlson comorbidity index	3.1405 (<0.001)*	3.7237 (<0.001)*	3.8508 (<0.001)*		2.8516 (<0.001)*	3.0713 (<0.001)*		3.6258 (<0.001)*
Count of Urea nitrogen in blood test	1.0023 (0.008)*	1.0001 (<0.001)*	1.0001 (0.60)	2.8257 (<0.001)*	1.0003 (<0.001)*	3.5675 (<0.001)*	1.0 (0.45)	1.0 (0.19)
Count of Hematocrit in blood test	1.0001 (<0.001)*	1.0003 (0.01)	1.0005 (0.19)	1.0 (0.14)	1.0 (0.52)	3.4812 (<0.001)*	1.0001 (0.14)	1.0 (0.06)
Number of hemoglobin tests within 8-365 days prior to surgery	2.1812 (0.002)*	1.0002 (0.01)	1.9043 (0.12)	1.0001 (0.03)	2.2381 (<0.001)*	1.0006 (0.49)	1.2185 (0.43)	2.3553 (<0.001)*
Count of The amount of hemoglobin relative to the size of the cell in blood test	2.6571 (0.001)*	2.6011 (<0.001)*	2.7616 (<0.001)*	3.285 (<0.001)*	3.0712 (0.002)*	2.5565 (<0.001)*	2.8277 (<0.001)*	2.4768 (<0.001)*
Count of anion gap in blood test	1.0001 (0.52)	1.0001 (0.30)	2.1112 (0.17)		1.0 (0.58)		1.2432 (0.43)	
Count of Serum Calcium test	3.4779 (0.001)*	3.3747 (<0.001)*	3.3759 (<0.001)*		1.0006 (0.002)*		3.5627 (<0.001)*	1.0 (0.08)
Count of Serum CO2 test	1.0001 (0.005)*	2.5936 (<0.001)*	1.0 (0.06)		1.4498 (<0.001)*		1.0002 (0.07)	1.0 (0.15)
Count of Potassium in serum test	1.0003 (0.35)	2.8823 (<0.001)*	3.2111 (<0.001)*		2.8138 (0.003)*	1.0002 (0.49)	2.0331 (<0.001)*	
Count of Urea nitrogen-Creatinine ratio	2.992 (0.005)*	1.758 (0.36)	2.6765 (0.004)*		2.353 (0.15)		2.8918 (0.03)	
Count of White Blood Cell in blood test	2.4464 (0.04)		1.7353 (0.001)*	3.1648 (<0.001)*	1.0001 (0.70)	2.9246 (0.01)	1.0005 (>0.99)	
County of home address	1.0005 (0.40)	2.1143 (0.21)	1.0 (0.002)*	1.0001 (0.006)*	3.2485 (0.01)	1.0009 (0.11)	2.749 (0.31)	1.0001 (0.41)
Average of Hematocrit in blood, %	2.4076 (<0.001)*	1.0002 (0.04)		1.0012 (<0.001)*	1.0001 (0.001)*	1.0021 (0.43)	1.0003 (<0.001)*	1.0003 (0.49)
Max of Hematocrit in blood, %	1.0013 (0.34)	2.3352 (0.48)			1.1377 (0.05)		1.0002 (<0.001)*	2.5295 (0.20)
Min of Hematocrit in blood, %	1.0002 (<0.001)*	1.0002 (0.05)			1.0003 (0.25)		1.0001 (<0.001)*	

© 2022 Ren Y et al. *JAMA Network Open*.

Feature	Cardiovascular complication	Prolonged ICU stay	Neurological complication and delirium	Wound complication	Sepsis	Venous thromboembolism	Prolonged mechanical ventilation	Acute kidney injury
Average of hemoglobin within 7 days prior to surgery, g/dl	1.0004 (<0.001)*	2.1171 (<0.001)*		2.6439 (<0.001)*	3.0839 (<0.001)*	1.0003 (0.01)	3.0044 (<0.001)*	3.5691 (<0.001)*
Max hemoglobin within 7 days prior to surgery, g/dl	2.9044 (0.02)	1.9698 (<0.001)*			3.1343 (<0.001)*	1.0003 (<0.001)*	1.7069 (<0.001)*	1.0003 (0.15)
Average of Mean platelet volume in blood, fL	1.0005 (0.51)		2.194 (0.39)	3.1908 (<0.001)*	2.9737 (<0.001)*		1.0001 (0.06)	3.3174 (0.006)*
Min of Mean platelet volume in blood, fL	3.0718 (0.02)		1.2102 (0.48)				1.0002 (0.01)	
Insurance paying the bills	1.9087 (<0.001)*	1.9504 (<0.001)*	1.0 (<0.001)*	1.7376 (<0.001)*	1.0 (<0.001)*		1.7585 (<0.001)*	1.8347 (<0.001)*
Average of platelet in blood, thou/uL	1.5431 (<0.001)*			1.0001 (<0.001)*	3.0251 (<0.001)*		2.3102 (0.002)*	3.0969 (<0.001)*
Average of Mean Corpuscular Volume in blood, fL	1.6356 (<0.001)*			2.3119 (0.009)*			1.0001 (<0.001)*	1.0001 (0.006)*
Average of Red cell distribution width in Blood, %	3.6986 (<0.001)*	2.5873 (0.001)*	1.0012 (0.18)	2.9061 (<0.001)*	3.1853 (<0.001)*	3.3818 (<0.001)*	2.945 (<0.001)*	2.2815 (0.03)
Scheduled surgery room	3.2273 (<0.001)*	3.6887 (<0.001)*	3.897 (<0.001)*	3.207 (<0.001)*	1.8141 (<0.001)*	2.3654 (<0.001)*	1.0004 (<0.001)*	3.842 (<0.001)*
Average of anion gap in blood, mmol/L	2.9935 (<0.001)*	2.5956 (<0.001)*	2.4366 (0.03)		1.0001 (0.003)*		1.8562 (<0.001)*	2.1538 (<0.001)*
Average of Serum Calcium, mmol/L	3.261 (<0.001)*	3.5946 (<0.001)*	2.0778 (<0.001)*	3.18 (<0.001)*	2.6519 (<0.001)*	2.731 (<0.001)*	2.6 (0.005)*	3.0869 (<0.001)*
Min of Glucose in blood, mg/dL	1.5262 (0.02)	1.8191 (<0.001)*	1.0 (<0.001)*		2.3984 (0.02)		1.0003 (<0.001)*	
Variance of Glucose in blood, mg/dL	2.2085 (<0.001)*							
Average of Serum Sodium, mmol/L	2.7147 (<0.001)*		2.787 (<0.001)*		3.5838 (<0.001)*	1.0003 (0.14)	3.067 (<0.001)*	2.682 (<0.001)*
Average of Urea nitrogen-Creatinine ratio	1.0001 (0.87)	1.0002 (<0.001)*	1.0402 (0.06)		3.3709 (0.10)	1.0009 (0.98)	1.4945 (0.42)	2.9943 (<0.001)*
Max of Urea nitrogen-Creatinine ratio	2.8814 (0.02)				1.0 (0.51)			

© 2022 Ren Y et al. *JAMA Network Open*.

Feature	Cardiovascular complication	Prolonged ICU stay	Neurological complication and delirium	Wound complication	Sepsis	Venous thromboembolism	Prolonged mechanical ventilation	Acute kidney injury
Min of Urea nitrogen-Creatinine ratio	1.0002 (0.29)				1.0001 (0.20)	2.8032 (0.004)*	1.0015 (0.03)	
Surgery Type	2.5561 (<0.001)*							
Time from Admission to Surgery, days	3.7243 (<0.001)*	3.553 (<0.001)*	2.1422 (<0.001)*	1.5772 (0.04)	2.6719 (<0.001)*	1.0001 (<0.001)*		
Average of White Blood Cell in blood, thou/uL	1.8353 (0.005)*	1.0001 (<0.001)*	1.9531 (0.003)*		2.4584 (0.13)	1.0002 (0.03)	1.4025 (<0.001)*	1.0 (<0.001)*
ZIP Code of home address	1.0002 (<0.001)*	2.7398 (<0.001)*	2.1703 (<0.001)*	1.1752 (<0.001)*	2.9904 (<0.001)*	2.096 (<0.001)*	3.0762 (<0.001)*	1.8391 (<0.001)*
CKD status at admission		1.9649 (<0.001)*	1.8479 (<0.001)*	1.7849 (0.05)			1.0001 (<0.001)*	
Number of urine glucose tests within 0-7 days prior to surgery		1.5322 (<0.001)*	1.0 (<0.001)*	3.2282 (0.008)*	2.8141 (<0.001)*		1.0001 (<0.001)*	
Count of Glucose in blood test		1.0002 (<0.001)*	2.1461 (0.09)	1.0001 (0.006)*		2.8083 (0.08)	2.2225 (<0.001)*	
Count of Serum Sodium test		1.0002 (0.004)*	1.1406 (<0.001)*		1.0 (<0.001)*	2.1978 (0.11)	3.0697 (0.005)*	
Variance of Hematocrit in blood, %		1.0001 (0.67)	3.46 (0.001)*					2.4539 (0.18)
Average of hemoglobin within 8-365 days prior to surgery, g/dl		2.211 (0.21)		1.7376 (0.56)			1.0004 (0.07)	1.0 (<0.001)*
Automated urinalysis, urine hemoglobin within 8-365 days prior to surgery, mg/dL		2.0008 (0.04)						
Variance of Serum CO2, mmol/L		1.0002 (0.02)					1.0002 (0.22)	
Average of Glucose in blood, mg/dL		3.3199 (0.009)*	1.0002 (0.85)		2.6762 (0.33)		2.4198 (0.48)	2.1562 (<0.001)*
Max of Glucose in blood, mg/dL		1.0027 (0.008)*	2.823 (0.09)	1.0001 (0.03)	1.0001 (0.55)		2.1544 (0.02)	

© 2022 Ren Y et al. *JAMA Network Open*.

Feature	Cardiovascular complication	Prolonged ICU stay	Neurological complication and delirium	Wound complication	Sepsis	Venous thromboembolism	Prolonged mechanical ventilation	Acute kidney injury
Smoking Status		1.0009 (<0.001)*	1.0001 (<0.001)*				1.0001 (<0.001)*	
Number of urine glucose tests within 8-365 days prior to surgery			1.0 (0.03)	1.0001 (0.001)*	1.7839 (0.43)			
Automated urinalysis, urine glucose within 8-365 days prior to surgery, mg/dL			1.5472 (0.16)		1.0 (0.98)	1.7837 (0.05)		1.0 (<0.001)*
Max hemoglobin within 8-365 days prior to surgery, g/dl			1.0005 (0.07)	2.1597 (0.23)	2.2442 (0.12)	1.0002 (<0.001)*	1.0005 (0.03)	
Race			1.8221 (0.001)*					
Min of anion gap in blood, mmol/L			1.0 (0.19)					
Average of Serum CO2, mmol/L			2.6423 (<0.001)*		2.7259 (<0.001)*			
Average of Potassium in serum, mmol/L			2.351 (0.14)	1.0001 (0.01)	1.0 (<0.001)*			
Number of urine hemoglobin tests within 8-365 days prior to surgery				2.0901 (0.06)		1.5937 (0.44)		
Ethnicity				1.0001 (0.01)*			1.0001 (0.005)*	
Variance of hemoglobin within 8-365 days prior to surgery, g/dl				2.7605 (0.001)*		1.0008 (<0.001)*		
Median total income at patient residential area, USD				1.0001 (0.02)				
Variance of Urea nitrogen-Creatinine ratio				2.1077 (0.18)			1.0001 (<0.001)*	1.0 (<0.001)*
Min hemoglobin within 8-365 days prior to surgery, g/dl					1.6605 (0.10)	2.4968 (<0.001)*		

© 2022 Ren Y et al. *JAMA Network Open*.

Feature	Cardiovascular complication	Prolonged ICU stay	Neurological complication and delirium	Wound complication	Sepsis	Venous thromboembolism	Prolonged mechanical ventilation	Acute kidney injury
Max of platelet in blood, thou/uL					1.0 (<0.001)*			
Variance of platelet in blood, thou/uL					1.0 (0.51)	1.0002 (0.04)		
Max of White Blood Cell in blood, thou/uL					3.3929 (<0.001)*			
Variance of hemoglobin within 7 days prior to surgery, g/dl						1.7008 (0.01)	2.1818 (<0.001)*	
Average of The amount of hemoglobin relative to the size of the cell in blood, g/dL						1.3203 (0.53)	1.0001 (0.26)	
Rural or city at patient residential area						1.0003 (0.03)		1.0 (0.02)
Marital Status							1.0001 (0.18)	
Min of Serum Calcium, mmol/L							1.0002 (0.14)	1.0 (0.02)
Variance of Serum Calcium, mmol/L								2.0348 (0.02)

*Statistically significant feature for the model.
Abbreviations: ICU, intensive care unit; ACE, Angiotensin-converting enzyme; CKD, chronic kidney disease.

© 2022 Ren Y et al. *JAMA Network Open*.

eTable 6. Summary of the Contributions of Each Input Feature to Each Outcome for a Generalized Additive Model With 135 Input Features

Feature	Cardiovascular complication	Prolonged ICU stay	Neurological complication and delirium	Wound complication	Sepsis	Venous thromboembolism	Prolonged mechanical ventilation	Acute kidney injury
	**Odds ratio (99% confidence interval)**							
Indicator of receiving ACE Inhibitors within one year before admission date	0.97 (0.82, 1.14)	1.12 (0.95, 1.32)	0.97 (0.81, 1.15)	0.92 (0.78, 1.09)	0.97 (0.81, 1.16)	0.99 (0.79, 1.23)		1.0 (0.85, 1.18)
Admission Source (Transfer vs other)	1.25 (1.11, 1.41)*	1.46 (1.3, 1.64)*	1.33 (1.17, 1.51)*	1.07 (0.95, 1.2)	1.31 (1.15, 1.5)*	1.29 (1.1, 1.51)*	1.41 (1.2, 1.65)*	1.36 (1.21, 1.52)*
Alcohol abuse or drug	0.94 (0.84, 1.06)	1.05 (0.94, 1.17)	1.15 (1.01, 1.31)*	1.07 (0.96, 1.19)	0.98 (0.86, 1.12)	0.94 (0.8, 1.1)	1.02 (0.86, 1.21)	1.1 (0.98, 1.23)
Indicator of receiving Aminoglycosides within one year before admission date	1.03 (0.81, 1.31)			1.1 (0.89, 1.36)	1.1 (0.86, 1.42)	1.17 (0.87, 1.57)		1.1 (0.87, 1.38)
Chronic anemia	1.1 (0.99, 1.23)	1.01 (0.9, 1.12)		1.27 (1.15, 1.4)*	1.16 (1.01, 1.32)*	1.26 (1.08, 1.48)*	0.97 (0.81, 1.16)	1.13 (1.02, 1.26)*
Anesthesia Type (Local vs general)	0.99 (0.85, 1.16)	0.81 (0.69, 0.96)*	0.83 (0.68, 1.0)	0.63 (0.54, 0.74)*	0.74 (0.59, 0.93)*		0.65 (0.45, 0.93)*	0.94 (0.8, 1.09)
Indicator of receiving Aspirin within one year before admission date	0.91 (0.8, 1.04)	0.78 (0.68, 0.89)*		1.03 (0.91, 1.16)		0.88 (0.71, 1.09)		0.89 (0.77, 1.01)
Indicator of receiving Bicarbonate within one year before admission date	0.9 (0.74, 1.09)				0.8 (0.63, 1.01)	1.04 (0.8, 1.36)		0.84 (0.69, 1.04)
Coagulopathy	1.09 (0.98, 1.22)	1.07 (0.95, 1.2)	1.28 (1.12, 1.46)*	1.14 (1.03, 1.27)*	1.19 (1.04, 1.37)*	1.52 (1.3, 1.77)*	1.18 (0.98, 1.41)	1.08 (0.96, 1.21)
Depression	1.12 (1.02, 1.23)*		1.17 (1.05, 1.3)*	1.06 (0.97, 1.15)				
Diabetes	0.83 (0.74, 0.92)*	0.88 (0.79, 0.98)*	0.79 (0.7, 0.9)*			0.86 (0.74, 1.01)	0.71 (0.59, 0.84)*	
Emergent or elective admission	1.13 (1.01, 1.27)*	1.17 (1.04, 1.31)*	0.87 (0.77, 0.99)*	0.78 (0.7, 0.86)*	0.74 (0.64, 0.86)*	0.75 (0.64, 0.89)*		

© 2022 Ren Y et al. *JAMA Network Open*.

Feature	Cardiovascular complication	Prolonged ICU stay	Neurological complication and delirium	Wound complication	Sepsis	Venous thromboembolism	Prolonged mechanical ventilation	Acute kidney injury
Hypertension	1.03 (0.92, 1.14)	1.27 (1.15, 1.4)*	1.3 (1.15, 1.46)*	1.05 (0.95, 1.15)		1.17 (1.01, 1.36)*	1.3 (1.09, 1.54)*	1.27 (1.15, 1.41)*
Cancer	1.06 (0.94, 1.2)		0.87 (0.78, 0.97)*	0.98 (0.88, 1.1)				
Congestive Heart Failure	1.41 (1.27, 1.57)*	1.27 (1.14, 1.43)*	1.22 (1.07, 1.39)*				1.39 (1.17, 1.65)*	1.27 (1.14, 1.42)*
Chronic Pulmonary Disease	1.02 (0.93, 1.12)	1.02 (0.93, 1.12)		1.01 (0.92, 1.1)			1.13 (0.98, 1.3)	
Peripheral Vascular Disease	1.07 (0.96, 1.18)	1.0 (0.91, 1.11)	0.9 (0.8, 1.01)	0.99 (0.9, 1.1)	0.98 (0.86, 1.11)	0.89 (0.76, 1.04)		0.97 (0.88, 1.08)
Liver Disease	1.03 (0.91, 1.15)	0.92 (0.82, 1.04)	0.91 (0.79, 1.05)	0.88 (0.78, 0.98)*				
Fluid and electrolyte disorders	1.43 (1.3, 1.57)*	1.39 (1.26, 1.53)*		1.12 (1.02, 1.23)*	1.4 (1.24, 1.58)*	1.12 (0.97, 1.3)	1.29 (1.11, 1.5)*	1.21 (1.1, 1.33)*
Yes if the admission we happened at night	1.07 (0.99, 1.16)	1.3 (1.2, 1.4)*		0.95 (0.89, 1.03)				
Scheduled post operation location	0.64 (0.58, 0.71)*	0.26 (0.24, 0.28)*	0.65 (0.59, 0.73)*	0.89 (0.81, 0.98)*	0.65 (0.57, 0.73)*	0.81 (0.7, 0.94)*	0.48 (0.41, 0.56)*	0.8 (0.72, 0.89)*
Scheduled room is trauma room or not	1.78 (1.19, 2.68)*		1.24 (0.83, 1.85)		0.87 (0.55, 1.37)	1.38 (0.86, 2.22)	1.25 (0.82, 1.92)	1.27 (0.87, 1.85)
Sex (Male vs female)	0.9 (0.83, 0.98)*	1.03 (0.94, 1.12)						0.87 (0.79, 0.95)*
Weight Loss	1.6 (1.44, 1.77)*	1.98 (1.78, 2.2)*	1.59 (1.41, 1.8)*	1.36 (1.24, 1.51)*	1.54 (1.36, 1.75)*	1.32 (1.14, 1.54)*	2.16 (1.85, 2.53)*	1.2 (1.08, 1.35)*
Indicator of receiving Antiemetic within one year before admission date		1.02 (0.9, 1.15)	0.82 (0.72, 0.94)*	1.27 (1.13, 1.43)*	0.97 (0.84, 1.12)		0.87 (0.71, 1.08)	
Indicator of receiving Beta-blockers within one year before admission date		1.02 (0.89, 1.17)	0.82 (0.7, 0.96)*	1.14 (1.01, 1.28)*	0.88 (0.75, 1.04)	1.03 (0.85, 1.26)		1.1 (0.96, 1.27)

© 2022 Ren Y et al. *JAMA Network Open*.

Feature	Cardiovascular complication	Prolonged ICU stay	Neurological complication and delirium	Wound complication	Sepsis	Venous thromboembolism	Prolonged mechanical ventilation	Acute kidney injury
Indicator of receiving Diuretics within one year before admission date		1.16 (0.98, 1.38)						1.11 (0.94, 1.32)
Cerebrovascular Disease		1.18 (1.07, 1.31)*	3.01 (2.71, 3.35)*	0.92 (0.83, 1.02)	0.93 (0.81, 1.07)	1.19 (1.03, 1.38)*	1.54 (1.33, 1.78)*	
Metastatic Carcinoma		1.4 (1.1, 1.79)*						1.12 (0.9, 1.4)
Myocardial Infarction			1.0 (0.85, 1.17)					
Valvular Disease			0.91 (0.79, 1.04)				0.91 (0.76, 1.09)	
Obesity				1.23 (1.13, 1.34)*				
Indicator of receiving pressors or inotropes within one year before admission date				1.34 (1.15, 1.58)*				
Indicator of receiving Vancomycin within one year before admission date				1.09 (0.93, 1.29)	1.25 (1.05, 1.48)*			
Indicator of receiving statin within one year before admission date						0.83 (0.65, 1.05)	0.74 (0.58, 0.96)*	
Age							1.77 (1.13, 2.78)*	
CKD status at admission								0.86 (0.74, 0.99)*
	**Degrees of freedom (p value)**							
Admission Day	1.0001 (0.95)	1.0229 (0.006)*	1.0 (0.10)		1.0 (0.58)	2.5342 (0.08)	1.0001 (0.32)	1.959 (0.07)
Admission Month	0.9999 (<0.001)*	1.0 (0.001)*	1.0002 (0.003)*	1.0001 (<0.001)*	1.0 (<0.001)*		1.0 (0.05)	1.0002 (0.005)*
Medicine or surgery admitting	1.4037 (<0.001)*	1.4696 (0.02)	1.5476 (0.51)	1.6998 (0.08)	1.9494 (<0.001)*	1.0003 (0.16)	1.7868 (0.005)*	1.0003 (<0.001)*

© 2022 Ren Y et al. *JAMA Network Open*.

Feature	Cardiovascular complication	Prolonged ICU stay	Neurological complication and delirium	Wound complication	Sepsis	Venous thromboembolism	Prolonged mechanical ventilation	Acute kidney injury
Age	3.1804 (<0.001)*	2.7728 (<0.001)*	1.0 (<0.001)*	2.3179 (<0.001)*	2.5619 (0.002)*		2.8592 (0.005)*	2.8137 (<0.001)*
Body Mass Index	2.592 (0.006)*	2.2898 (<0.001)*	1.5529 (0.70)				1.6114 (<0.001)*	2.6882 (<0.001)*
Average of Urea nitrogen in blood, mg/dL	1.0016 (0.30)	3.5295 (0.02)	1.9668 (0.15)	1.3352 (0.86)	1.0 (0.44)	1.0002 (0.83)	1.0 (0.58)	1.0 (0.22)
Max of Urea nitrogen in blood, mg/dL	0.9999 (0.02)	2.9952 (0.39)	1.0 (0.72)	2.4666 (0.42)	1.8044 (0.007)*	1.0002 (0.17)	1.176 (0.11)	3.0335 (0.05)
Min of Urea nitrogen in blood, mg/dL	1.0003 (0.25)		1.0052 (0.07)		2.5204 (0.28)	1.0003 (0.19)	1.0 (0.10)	1.0002 (0.01)
Variance of Urea nitrogen in blood, mg/dL	2.9475 (0.02)	1.56 (0.15)	1.3002 (0.84)		2.3016 (0.04)		1.904 (0.05)	1.0001 (0.65)
Charlson comorbidity index	2.5534 (0.01)	3.2181 (<0.001)*		2.024 (0.004)*	2.4515 (<0.001)*	1.7816 (0.43)		3.1768 (<0.001)*
Count of Urea nitrogen in blood test	1.0041 (0.02)	1.0 (0.11)	1.0003 (0.39)	3.3608 (<0.001)*	1.0003 (0.06)	1.0031 (0.10)	1.0 (0.07)	3.5806 (<0.001)*
Count of Hematocrit in blood test	1.9502 (0.02)	2.8642 (<0.001)*	1.8061 (0.15)	1.0001 (0.91)	2.7557 (0.04)	3.6088 (<0.001)*	2.3802 (0.09)	1.0028 (0.28)
Number of hemoglobin tests within 7 days prior to surgery	3.7266 (<0.001)*	3.2706 (<0.001)*	3.2141 (<0.001)*		3.5983 (<0.001)*	3.0927 (0.008)*	3.3118 (<0.001)*	1.0002 (0.06)
Number of hemoglobin tests within 8-365 days prior to surgery	1.0004 (0.94)	1.0 (0.39)			1.0 (0.33)	1.0004 (0.14)		1.0005 (0.47)
Number of urine hemoglobin tests within 8-365 days prior to surgery	2.8695 (0.04)	2.3011 (0.12)		2.6853 (<0.001)*		1.4275 (0.29)		
Count of anion gap in blood test	1.0004 (0.35)	1.0004 (0.84)	1.0 (>0.99)	1.0002 (0.05)	1.0 (0.17)		1.9199 (0.38)	
Count of Serum Calcium test	3.453 (<0.001)*	3.5665 (<0.001)*	3.3993 (<0.001)*		1.0 (<0.001)*	3.5802 (<0.001)*	3.4157 (<0.001)*	1.0008 (0.41)
Count of chloride in Serum test	0.9982 (0.31)	0.9999 (0.02)	1.0 (0.03)		1.0 (0.010)*		1.0 (0.06)	1.0002 (0.08)

© 2022 Ren Y et al. *JAMA Network Open*.

Feature	Cardiovascular complication	Prolonged ICU stay	Neurological complication and delirium	Wound complication	Sepsis	Venous thromboembolism	Prolonged mechanical ventilation	Acute kidney injury
Count of Serum CO2 test	0.998 (0.09)	2.4069 (<0.001)*	1.0 (0.02)		1.0 (<0.001)*		1.0 (0.40)	1.0002 (0.86)
Count of Serum creatinine test	0.9948 (0.21)	1.0 (0.49)	1.0001 (0.56)		1.0 (0.43)		1.0 (0.11)	1.0002 (0.09)
Count of Glucose in blood test	1.1331 (0.06)	1.4368 (<0.001)*	2.3539 (0.07)	1.0001 (0.02)			2.395 (0.05)	1.4104 (0.26)
Count of Urea nitrogen-Creatinine ratio	2.7667 (<0.001)*	1.0071 (0.09)	2.7053 (<0.001)*	2.9709 (<0.001)*	2.7317 (0.001)*	1.5574 (0.08)	3.2439 (0.005)*	
Count of White Blood Cell in blood test	1.0011 (0.004)*			2.4698 (0.005)*			1.0 (0.04)	1.0003 (0.64)
County of home address	1.0003 (0.24)	2.1476 (0.06)	1.0 (0.15)	1.0001 (0.92)	3.2144 (0.01)	1.8668 (0.48)	3.0422 (0.15)	1.0004 (0.30)
Average of Hematocrit in blood, %	0.9991 (0.004)*	1.0 (0.31)	1.0 (0.54)	1.6382 (0.39)	1.2501 (0.71)	1.8565 (0.42)	1.0001 (0.08)	1.0003 (0.16)
Min of Hematocrit in blood, %	1.0006 (0.40)	1.0 (0.16)	1.0 (0.56)		2.0952 (0.22)		1.0 (0.46)	1.0015 (0.16)
Average of hemoglobin within 7 days prior to surgery, g/dl	1.0004 (0.82)	1.0 (0.79)		1.0002 (0.01)	1.0003 (0.40)	1.0047 (0.43)	1.0 (0.20)	3.3344 (<0.001)*
Max hemoglobin within 8-365 days prior to surgery, g/dl	1.7089 (0.47)		1.0 (0.88)		2.2152 (0.22)	1.0002 (0.20)	1.0 (0.93)	
Min hemoglobin within 7 days prior to surgery, g/dl	0.9996 (0.04)	1.0 (0.53)			1.0 (0.13)		1.0 (0.67)	
Automated urinalysis, urine hemoglobin within 7 days prior to surgery, mg/dL	2.3868 (0.02)	1.0001 (0.003)*		1.5007 (<0.001)*	2.0963 (0.006)*		1.0 (0.12)	1.1077 (0.003)*
No of nephrotoxic drugs received within one year before admission date	1.0003 (0.34)	1.0002 (<0.001)*		2.4355 (0.04)			1.0 (0.01)	2.3922 (0.02)
Insurance paying the bills	1.8709 (<0.001)*			1.8447 (<0.001)*	1.0 (<0.001)*		1.4688 (<0.001)*	
Average of platelet in blood, thou/uL	1.0002 (<0.001)*			1.0001 (0.003)*	1.0003 (<0.001)*		1.3803 (0.03)	2.7968 (<0.001)*

© 2022 Ren Y et al. *JAMA Network Open*.

Feature	Cardiovascular complication	Prolonged ICU stay	Neurological complication and delirium	Wound complication	Sepsis	Venous thromboembolism	Prolonged mechanical ventilation	Acute kidney injury
Current Procedural Terminology code of the primary procedure	2.1895 (<0.001)*	3.6941 (<0.001)*	1.0001 (<0.001)*	3.6746 (<0.001)*	2.9481 (<0.001)*	3.424 (<0.001)*	2.9367 (<0.001)*	3.78 (<0.001)*
Prevalence of Hispanic residents living below poverty at patient residential area, %	3.0012 (0.01)	1.9781 (0.09)				2.4555 (0.13)	2.4953 (0.23)	3.3155 (0.01)
Average of Serum Red Blood Cell, Million/uL	1.0001 (<0.001)*				2.3176 (0.004)*			
Average of Red cell distribution width in Blood, %	1.0003 (0.08)	1.4606 (0.02)		2.8154 (0.002)*	2.8973 (<0.001)*	3.2733 (<0.001)*	2.1145 (0.009)*	1.0006 (0.03)
Scheduled surgery room	3.1381 (<0.001)*	3.7737 (<0.001)*	3.4651 (<0.001)*	2.5576 (<0.001)*	1.8007 (<0.001)*	1.6766 (<0.001)*	1.0001 (<0.001)*	3.6539 (<0.001)*
Average of anion gap in blood, mmol/L	2.6472 (0.02)	2.1831 (0.04)	2.6037 (0.07)		1.8282 (0.007)*		1.0 (0.005)*	1.0003 (0.54)
Average of Serum Calcium, mmol/L	2.9213 (<0.001)*	3.6645 (<0.001)*	1.9944 (0.004)*		2.3533 (<0.001)*	2.3327 (0.002)*	2.3014 (<0.001)*	1.4623 (0.001)*
Average of chloride in Serum, mmol/L	3.1405 (0.24)		2.2056 (0.03)		1.0 (0.04)		2.3449 (0.11)	1.6834 (0.002)*
Max of chloride in Serum, mmol/L	0.9999 (0.03)							
Average of Serum CO2, mmol/L	2.7689 (<0.001)*		2.1442 (0.007)*		1.928 (0.37)			2.0814 (0.24)
Variance of Serum CO2, mmol/L	2.696 (0.11)							
Min of Serum creatinine, mg/dL	2.7931 (0.03)				1.0003 (0.21)	1.0002 (0.48)		
Variance of Serum creatinine, mg/dL	3.5031 (<0.001)*			1.6443 (0.05)		1.0001 (0.09)		
Min of Glucose in blood, mg/dL	1.3524 (0.01)	1.584 (<0.001)*	1.0 (0.006)*		1.6914 (0.11)		1.0 (0.01)	
Average of Urea nitrogen-Creatinine ratio	0.9999 (0.22)	1.0 (0.07)	1.0 (0.63)			2.5 (0.12)		

© 2022 Ren Y et al. *JAMA Network Open*.

Feature	Cardiovascular complication	Prolonged ICU stay	Neurological complication and delirium	Wound complication	Sepsis	Venous thromboembolism	Prolonged mechanical ventilation	Acute kidney injury
Min of Urea nitrogen-Creatinine ratio	1.0003 (0.47)							
Surgery Type	1.5014 (<0.001)*		3.422 (<0.001)*			1.8883 (0.22)		
Time from Admission to Surgery, days	2.8035 (<0.001)*	3.5861 (<0.001)*	1.535 (0.002)*	1.0843 (0.009)*	2.8486 (<0.001)*	1.0005 (0.010)*	1.0 (0.003)*	1.6912 (0.07)
Average of White Blood Cell in blood, thou/uL	1.7238 (0.003)*	1.0 (<0.001)*	1.0 (0.003)*		1.0002 (0.009)*	1.0005 (<0.001)*	1.0002 (<0.001)*	1.2818 (<0.001)*
ZIP Code of home address	1.0003 (<0.001)*	2.8352 (<0.001)*	2.1945 (<0.001)*	1.8761 (<0.001)*	3.0425 (<0.001)*	2.1701 (<0.001)*	3.2853 (<0.001)*	1.4936 (<0.001)*
CKD status at admission		1.8763 (0.03)	1.2343 (0.03)	1.3923 (0.03)			1.0 (<0.001)*	
Count of Potassium in serum test		3.2974 (<0.001)*	3.3579 (0.009)*		2.9791 (<0.001)*	1.0003 (0.77)	2.6344 (0.10)	
Reference estimated glomerular filtration rate		3.8031 (<0.001)*	3.2171 (<0.001)*	1.0049 (0.004)*	2.9283 (0.001)*		2.9152 (<0.001)*	3.7619 (<0.001)*
Variance of Hematocrit in blood, %		1.0 (0.63)	3.2207 (0.24)					
Average of hemoglobin within 8-365 days prior to surgery, g/dl		1.5662 (0.02)						
Max hemoglobin within 7 days prior to surgery, g/dl		3.0535 (0.02)			2.6628 (0.09)		2.4919 (0.16)	
Min hemoglobin within 8-365 days prior to surgery, g/dl		1.0001 (0.001)*		1.0006 (0.01)				
Average of Serum creatinine, mg/dL		2.2507 (0.37)			3.3226 (0.04)		2.9116 (0.005)*	3.7715 (<0.001)*
Average of Glucose in blood, mg/dL		3.1911 (0.05)	1.0002 (0.65)	1.0001 (0.09)	1.0007 (0.78)		2.7123 (0.21)	2.1228 (<0.001)*
Max of Glucose in blood, mg/dL		1.0001 (0.14)	2.8146 (0.18)		1.0 (0.33)		1.4859 (0.38)	
Smoking Status		1.0 (0.05)	1.6674 (0.01)				1.0005 (<0.001)*	

© 2022 Ren Y et al. *JAMA Network Open*.

Feature	Cardiovascular complication	Prolonged ICU stay	Neurological complication and delirium	Wound complication	Sepsis	Venous thromboembolism	Prolonged mechanical ventilation	Acute kidney injury
Automated urinalysis, urine protein presence within 365 days prior to surgery, mg/dL		1.7046 (0.44)	1.0 (0.002)*				2.3406 (<0.001)*	2.5797 (<0.001)*
Count of Serum Sodium test			1.0005 (0.006)*		1.0 (<0.001)*	1.0004 (0.57)	2.8842 (0.05)	2.9619 (0.05)
Variance of Red cell distribution width in Blood, %			2.8065 (<0.001)*		1.0 (0.006)*			
Median total income at patient residential area, USD				1.0001 (0.07)				
Max of Red cell distribution width in Blood, %				1.0002 (0.02)				
Variance of chloride in Serum, mmol/L				1.0001 (0.02)	2.08 (0.17)		1.0 (0.37)	1.0002 (0.11)
Max of platelet in blood, thou/uL					1.0013 (<0.001)*			
Variance of platelet in blood, thou/uL					1.0 (0.55)	1.0001 (0.01)		
Average of Potassium in serum, mmol/L					1.0 (<0.001)*			2.0014 (0.02)
Average of Serum Sodium, mmol/L					3.4984 (<0.001)*		2.2246 (0.16)	
Variance of Urea nitrogen-Creatinine ratio					1.0 (0.10)			1.0001 (<0.001)*
Max of White Blood Cell in blood, thou/uL					3.4491 (<0.001)*			
Min of White Blood Cell in blood, thou/uL					3.0827 (0.03)			
Variance of White Blood Cell in blood, thou/uL					1.0 (0.97)	1.0004 (0.005)*		
Max of Serum Sodium, mmol/L						1.0004 (0.15)		

© 2022 Ren Y et al. *JAMA Network Open*.

Feature	Cardiovascular complication	Prolonged ICU stay	Neurological complication and delirium	Wound complication	Sepsis	Venous thromboembolism	Prolonged mechanical ventilation	Acute kidney injury
Ethnicity							1.0 (<0.001)*	
Min of platelet in blood, thou/uL							1.0003 (0.09)	
Max of Urea nitrogen-Creatinine ratio							1.0001 (0.003)*	
Max of Serum Calcium, mmol/L								2.7604 (0.003)*
Variance of Serum Calcium, mmol/L								2.1812 (0.07)
Max of Serum creatinine, mg/dL								3.7438 (<0.001)*

*Statistically significant feature for the model.
Abbreviations: ICU, intensive care unit; ACE, Angiotensin-converting enzyme; CKD, chronic kidney disease.

© 2022 Ren Y et al. *JAMA Network Open*.

eTable 7. Random Forest Model Performance Measurements for Complications With 95% CIs Calculated by 1000 Bootstrap Samples on the Validation Cohort

Complication	Input features (n)	Sensitivity	Specificity	PPV	NPV	Accuracy	AUROC	AUPRC	Threshold
Cardiovascular complication	55	0.71 (0.71, 0.72)	0.74 (0.73, 0.76)	0.34 (0.33, 0.35)	0.93 (0.93, 0.94)	0.72 (0.71, 0.72)	0.8 (0.79, 0.81)	0.46 (0.44, 0.48)	0.36
	101	0.71 (0.7, 0.71)	0.73 (0.71, 0.74)	0.33 (0.32, 0.34)	0.93 (0.93, 0.93)	0.71 (0.71, 0.72)	0.79 (0.78, 0.8)	0.44 (0.43, 0.46)	0.31
	135	0.73 (0.72, 0.74)	0.74 (0.73, 0.75)	0.35 (0.34, 0.36)	0.93 (0.93, 0.94)	0.73 (0.72, 0.74)	0.81 (0.81, 0.82)	0.48 (0.46, 0.5)	0.31
Prolonged ICU stay	55	0.78 (0.78, 0.79)	0.79 (0.78, 0.8)	0.59 (0.58, 0.6)	0.9 (0.9, 0.91)	0.78 (0.78, 0.79)	0.87 (0.87, 0.88)	0.76 (0.75, 0.77)	0.43
	101	0.72 (0.71, 0.73)	0.83 (0.82, 0.84)	0.54 (0.53, 0.55)	0.91 (0.91, 0.92)	0.75 (0.75, 0.76)	0.87 (0.86, 0.87)	0.74 (0.73, 0.75)	0.3
	135	0.77 (0.76, 0.77)	0.83 (0.82, 0.84)	0.59 (0.58, 0.6)	0.92 (0.91, 0.92)	0.78 (0.78, 0.79)	0.89 (0.88, 0.89)	0.78 (0.77, 0.79)	0.32
Neurological complication and delirium	55	0.79 (0.79, 0.8)	0.79 (0.77, 0.8)	0.41 (0.39, 0.42)	0.95 (0.95, 0.96)	0.79 (0.79, 0.8)	0.87 (0.86, 0.87)	0.6 (0.59, 0.62)	0.35
	101	0.77 (0.76, 0.77)	0.77 (0.76, 0.79)	0.37 (0.36, 0.38)	0.95 (0.95, 0.95)	0.77 (0.76, 0.77)	0.85 (0.84, 0.86)	0.56 (0.54, 0.57)	0.33
	135	0.78 (0.77, 0.78)	0.81 (0.8, 0.83)	0.39 (0.38, 0.41)	0.96 (0.96, 0.96)	0.78 (0.78, 0.79)	0.87 (0.87, 0.88)	0.61 (0.6, 0.63)	0.28
Wound complication	55	0.77 (0.76, 0.77)	0.65 (0.64, 0.66)	0.43 (0.42, 0.44)	0.89 (0.88, 0.89)	0.74 (0.73, 0.75)	0.78 (0.77, 0.79)	0.54 (0.53, 0.56)	0.42
	101	0.64 (0.63, 0.65)	0.67 (0.66, 0.68)	0.34 (0.33, 0.35)	0.88 (0.87, 0.88)	0.65 (0.64, 0.65)	0.71 (0.7, 0.72)	0.41 (0.4, 0.43)	0.34
	135	0.73 (0.73, 0.74)	0.68 (0.67, 0.7)	0.42 (0.41, 0.43)	0.89 (0.89, 0.9)	0.72 (0.72, 0.73)	0.78 (0.78, 0.79)	0.54 (0.53, 0.56)	0.35
Sepsis	55	0.74 (0.73, 0.75)	0.79 (0.77, 0.81)	0.23 (0.22, 0.24)	0.97 (0.97, 0.98)	0.74 (0.74, 0.75)	0.84 (0.83, 0.85)	0.38 (0.35, 0.4)	0.28
	101	0.78 (0.77, 0.78)	0.76 (0.74, 0.78)	0.25 (0.24, 0.26)	0.97 (0.97, 0.97)	0.78 (0.77, 0.78)	0.84 (0.83, 0.85)	0.4 (0.37, 0.42)	0.24
	135	0.76 (0.75, 0.76)	0.8 (0.79, 0.82)	0.24 (0.23, 0.25)	0.98 (0.97, 0.98)	0.76 (0.75, 0.77)	0.86 (0.85, 0.87)	0.43 (0.4, 0.45)	0.22
Venous thromboembolism	55	0.73 (0.72, 0.73)	0.77 (0.75, 0.79)	0.14 (0.14, 0.15)	0.98 (0.98, 0.98)	0.73 (0.73, 0.74)	0.82 (0.81, 0.83)	0.24 (0.22, 0.27)	0.22

© 2022 Ren Y et al. *JAMA Network Open*.

Complication	Input features (n)	Sensitivity	Specificity	PPV	NPV	Accuracy	AUROC	AUPRC	Threshold
	101	0.69 (0.68, 0.69)	0.79 (0.76, 0.81)	0.13 (0.12, 0.14)	0.98 (0.98, 0.98)	0.69 (0.69, 0.7)	0.81 (0.79, 0.82)	0.21 (0.19, 0.23)	0.17
	135	0.7 (0.69, 0.7)	0.8 (0.78, 0.83)	0.14 (0.13, 0.14)	0.98 (0.98, 0.99)	0.7 (0.7, 0.71)	0.82 (0.81, 0.83)	0.23 (0.21, 0.25)	0.16
Prolonged mechanical ventilation	55	0.82 (0.81, 0.82)	0.83 (0.81, 0.85)	0.21 (0.2, 0.22)	0.99 (0.99, 0.99)	0.82 (0.81, 0.82)	0.9 (0.89, 0.91)	0.44 (0.41, 0.46)	0.24
	101	0.8 (0.8, 0.81)	0.82 (0.8, 0.84)	0.2 (0.19, 0.21)	0.99 (0.98, 0.99)	0.8 (0.8, 0.81)	0.89 (0.88, 0.9)	0.4 (0.37, 0.43)	0.19
	135	0.84 (0.84, 0.85)	0.83 (0.8, 0.85)	0.23 (0.22, 0.25)	0.99 (0.99, 0.99)	0.84 (0.83, 0.84)	0.91 (0.9, 0.91)	0.44 (0.41, 0.47)	0.19
Acute kidney injury	55	0.73 (0.73, 0.74)	0.73 (0.71, 0.74)	0.35 (0.34, 0.36)	0.93 (0.93, 0.94)	0.73 (0.73, 0.74)	0.81 (0.8, 0.81)	0.47 (0.45, 0.49)	0.38
	101	0.69 (0.68, 0.69)	0.77 (0.75, 0.78)	0.32 (0.31, 0.33)	0.94 (0.93, 0.94)	0.7 (0.69, 0.71)	0.8 (0.79, 0.81)	0.46 (0.44, 0.48)	0.31
	135	0.75 (0.74, 0.76)	0.74 (0.72, 0.75)	0.37 (0.36, 0.38)	0.94 (0.93, 0.94)	0.75 (0.74, 0.75)	0.82 (0.82, 0.83)	0.52 (0.5, 0.53)	0.34
30-day mortality	55	0.68 (0.67, 0.68)	0.86 (0.82, 0.89)	0.05 (0.04, 0.05)	1.0 (1.0, 1.0)	0.68 (0.67, 0.69)	0.84 (0.82, 0.86)	0.1 (0.08, 0.13)	0.05
	101	0.81 (0.81, 0.82)	0.71 (0.66, 0.75)	0.07 (0.06, 0.07)	0.99 (0.99, 0.99)	0.81 (0.81, 0.82)	0.84 (0.82, 0.86)	0.09 (0.08, 0.11)	0.09
	135	0.76 (0.76, 0.77)	0.8 (0.76, 0.84)	0.06 (0.05, 0.07)	1.0 (0.99, 1.0)	0.76 (0.76, 0.77)	0.84 (0.82, 0.86)	0.1 (0.08, 0.12)	0.07
90-day mortality	55	0.7 (0.7, 0.71)	0.79 (0.76, 0.82)	0.07 (0.07, 0.08)	0.99 (0.99, 0.99)	0.71 (0.7, 0.71)	0.82 (0.81, 0.84)	0.13 (0.11, 0.16)	0.11
	101	0.69 (0.68, 0.7)	0.82 (0.79, 0.85)	0.07 (0.06, 0.08)	0.99 (0.99, 0.99)	0.69 (0.69, 0.7)	0.83 (0.81, 0.84)	0.13 (0.11, 0.15)	0.11
	135	0.73 (0.72, 0.74)	0.81 (0.77, 0.84)	0.08 (0.07, 0.09)	0.99 (0.99, 0.99)	0.73 (0.73, 0.74)	0.84 (0.82, 0.85)	0.13 (0.11, 0.15)	0.12

Abbreviations: PPV, positive predictive value; NPV, negative predictive value; AUROC, area under the receiver operating characteristic curve; AUPRC, area under the precision-recall curve; ICU, intensive care unit.

.

© 2022 Ren Y et al. *JAMA Network Open*.

eTable 8. Summary of the Contributions of Each Input Feature to Each Outcome for a Random Forest Model With 55 Input Features

**Cardiovascular complication**	**Prolonged ICU stay**	**Neurological complication and delirium**	**Wound complication**	**Sepsis**	**Venous thromboembolism**	**Prolonged mechanical ventilation**	**Acute kidney injury**
**Feature (importance weight)**							
Current Procedural Terminology code of the primary procedure (0.2176)	Current Procedural Terminology code of the primary procedure (0.3318)	Current Procedural Terminology code of the primary procedure (0.2566)	Current Procedural Terminology code of the primary procedure (0.3114)	Current Procedural Terminology code of the primary procedure (0.1849)	Current Procedural Terminology code of the primary procedure (0.1957)	Current Procedural Terminology code of the primary procedure (0.2792)	Current Procedural Terminology code of the primary procedure (0.1949)
ID of Attending Surgeon (0.1078)	ID of Attending Surgeon (0.1681)	ID of Attending Surgeon (0.1236)	ID of Attending Surgeon (0.1024)	Min hemoglobin within 7 days prior to surgery, g/dl (0.134)	ID of Attending Surgeon (0.0996)	ID of Attending Surgeon (0.1178)	ID of Attending Surgeon (0.1122)
Min hemoglobin within 7 days prior to surgery, g/dl (0.08)	Surgery Type (0.0893)	Cerebrovascular Disease (0.083)	ZIP Code of home address (0.0674)	Number of hemoglobin tests within 7 days prior to surgery (0.1245)	Number of hemoglobin tests within 7 days prior to surgery (0.0904)	Number of hemoglobin tests within 7 days prior to surgery (0.0963)	Min hemoglobin within 7 days prior to surgery, g/dl (0.0999)
Time from Admission to Surgery, days (0.0651)	Number of hemoglobin tests within 7 days prior to surgery (0.0499)	Surgery Type (0.071)	Time from Admission to Surgery, days (0.0661)	Time from Admission to Surgery, days (0.0826)	Min hemoglobin within 7 days prior to surgery, g/dl (0.09)	Min hemoglobin within 7 days prior to surgery, g/dl (0.0668)	Time from Admission to Surgery, days (0.0617)
Age (0.0599)	Min hemoglobin within 7 days prior to surgery, g/dl (0.0406)	Number of hemoglobin tests within 7 days prior to surgery (0.0568)	Min hemoglobin within 7 days prior to surgery, g/dl (0.0612)	ID of Attending Surgeon (0.08)	Time from Admission to Surgery, days (0.0877)	Time from Admission to Surgery, days (0.0613)	Reference estimated glomerular filtration rate (0.0608)
Reference estimated glomerular	Time from Admission to	Time from Admission to	Age (0.0542)	Reference estimated glomerular	ZIP Code of home address (0.0619)	Surgery Type (0.0592)	ZIP Code of home address (0.0576)

© 2022 Ren Y et al. *JAMA Network Open*.

**Cardiovascular complication**	**Prolonged ICU stay**	**Neurological complication and delirium**	**Wound complication**	**Sepsis**	**Venous thromboembolism**	**Prolonged mechanical ventilation**	**Acute kidney injury**
**Feature (importance weight)**							
filtration rate (0.0553)	Surgery, days (0.039)	Surgery, days (0.0522)		filtration rate (0.0464)			
ZIP Code of home address (0.0549)	Medicine or surgery admitting (0.0287)	Min hemoglobin within 7 days prior to surgery, g/dl (0.0426)	Reference estimated glomerular filtration rate (0.0486)	ZIP Code of home address (0.0436)	County of home address (0.0471)	Distance of residence to hospital, km (0.0328)	Age (0.0566)
Number of hemoglobin tests within 7 days prior to surgery (0.0536)	Age (0.0276)	Age (0.0389)	County of home address (0.0409)	Emergent or elective admission (0.041)	Surgery Type (0.04)	ZIP Code of home address (0.0327)	Surgery Type (0.0557)
Surgery Type (0.0477)	ZIP Code of home address (0.0266)	Distance of residence to hospital, km (0.0338)	Surgery Type (0.0372)	Surgery Type (0.0328)	Charlson comorbidity index (0.0399)	Admission Source (0.0278)	Number of hemoglobin tests within 7 days prior to surgery (0.0497)
County of home address (0.0346)	Distance of residence to hospital, km (0.0259)	ZIP Code of home address (0.0334)	Charlson comorbidity index (0.0296)	Charlson comorbidity index (0.0326)	Admission Day (0.0278)	Charlson comorbidity index (0.0254)	Charlson comorbidity index (0.0414)
Charlson comorbidity index (0.0314)	Admission Source (0.0246)	County of home address (0.0225)	Indicator of receiving pressors or inotropes within one year before admission date (0.0213)	Weight Loss (0.0307)	Emergent or elective admission (0.0232)	Weight Loss (0.0236)	County of home address (0.0347)
Weight Loss (0.0257)	Charlson comorbidity index (0.0211)	Medicine or surgery admitting (0.0225)	Number of hemoglobin tests within 7 days prior to	Admission Day (0.0206)	Smoking Status (0.0161)	County of home address (0.0228)	Hypertension (0.0225)

© 2022 Ren Y et al. *JAMA Network Open*.

Cardiovascular complication	Prolonged ICU stay	Neurological complication and delirium	Wound complication	Sepsis	Venous thromboembolism	Prolonged mechanical ventilation	Acute kidney injury
Feature (importance weight)							
			surgery (0.0199)				
Admission Day (0.0202)	County of home address (0.017)	Charlson comorbidity index (0.022)	Insurance paying the bills (0.0159)	Automated urinalysis, urine protein presence within 365 days prior to surgery, mg/dL (0.0165)	Automated urinalysis, urine protein presence within 365 days prior to surgery, mg/dL (0.0159)	Medicine or surgery admitting (0.0164)	Automated urinalysis, urine protein presence within 365 days prior to surgery, mg/dL (0.0165)
Congestive Heart Failure (0.0174)	Weight Loss (0.0168)	Admission Source (0.0192)	Medicine or surgery admitting (0.0127)	Admission Source (0.0151)	Insurance paying the bills (0.0154)	Admission Day (0.0156)	Insurance paying the bills (0.0146)
Insurance paying the bills (0.0166)	Admission Day (0.0116)	Admission Day (0.0142)	Indicator of receiving Vancomycin within one year before admission date (0.0125)	Insurance paying the bills (0.0141)	Admission Source (0.013)	Smoking Status (0.0134)	Smoking Status (0.0139)
Smoking Status (0.0137)	Smoking Status (0.0082)	Weight Loss (0.0134)	Weight Loss (0.0123)	Smoking Status (0.0123)	Medicine or surgery admitting (0.0122)	Automated urinalysis, urine protein presence within 365 days prior to surgery, mg/dL (0.0102)	Congestive Heart Failure (0.013)
Medicine or surgery admitting (0.0112)	Automated urinalysis, urine glucose within 7 days prior to surgery, mg/dL (0.0075)	Insurance paying the bills (0.0101)	Emergent or elective admission (0.0107)	Medicine or surgery admitting (0.012)	Chronic anemia (0.0111)	Insurance paying the bills (0.0096)	Peripheral Vascular Disease (0.0105)

© 2022 Ren Y et al. *JAMA Network Open*.

Cardiovascular complication	Prolonged ICU stay	Neurological complication and delirium	Wound complication	Sepsis	Venous thromboembolism	Prolonged mechanical ventilation	Acute kidney injury
**Feature (importance weight)**							
Automated urinalysis, urine protein presence within 365 days prior to surgery, mg/dL (0.0112)	Peripheral Vascular Disease (0.0072)	Emergent or elective admission (0.0096)	Chronic anemia (0.0084)	Automated urinalysis, urine glucose within 7 days prior to surgery, mg/dL (0.009)	Coagulopathy (0.011)	Cerebrovascular Disease (0.0088)	Weight Loss (0.008)
Peripheral Vascular Disease (0.0079)	Automated urinalysis, urine protein presence within 365 days prior to surgery, mg/dL (0.0068)	Smoking Status (0.0092)	Indicator of receiving Antiemetic within one year before admission date (0.008)	Hypertension (0.0073)	Weight Loss (0.0109)	Automated urinalysis, urine glucose within 7 days prior to surgery, mg/dL (0.008)	Medicine or surgery admitting (0.0075)
Admission Source (0.007)	Insurance paying the bills (0.0065)	Automated urinalysis, urine protein presence within 365 days prior to surgery, mg/dL (0.008)	Indicator of receiving Beta-blockers within one year before admission date (0.007)	Chronic anemia (0.0063)	Hypertension (0.0094)	Emergent or elective admission (0.0077)	Admission Source (0.0069)
Chronic anemia (0.0064)	Congestive Heart Failure (0.0056)	Indicator of receiving Antiemetic within one year before admission date (0.0058)	Hypertension (0.0069)	Peripheral Vascular Disease (0.0056)	Peripheral Vascular Disease (0.0093)	Indicator of receiving Antiemetic within one year before admission date (0.0066)	Chronic anemia (0.0062)
Chronic Pulmonary Disease (0.0062)	Hypertension (0.0054)	Sex (0.0057)	Chronic Pulmonary Disease (0.0068)	Diabetes (0.005)	Chronic Pulmonary Disease (0.0082)	Hypertension (0.0066)	Emergent or elective admission (0.0062)
Hypertension (0.0058)	Sex (0.0047)	Hypertension (0.0057)	Peripheral Vascular Disease (0.0064)	Congestive Heart Failure (0.0049)	Automated urinalysis, urine glucose within 7 days prior to	Congestive Heart Failure (0.0063)	Chronic Pulmonary Disease (0.006)

© 2022 Ren Y et al. *JAMA Network Open*.

Cardiovascular complication	Prolonged ICU stay	Neurological complication and delirium	Wound complication	Sepsis	Venous thromboembolism	Prolonged mechanical ventilation	Acute kidney injury
Feature (importance weight)							
					surgery, mg/dL (0.0074)		
Emergent or elective admission (0.0054)	Emergent or elective admission (0.0045)	Automated urinalysis, urine glucose within 7 days prior to surgery, mg/dL (0.0052)	Coagulopathy (0.0054)	Cerebrovascular Disease (0.0044)	Cerebrovascular Disease (0.0073)	Sex (0.0056)	Diabetes (0.0059)
Cerebrovascular Disease (0.0053)	Indicator of receiving Antiemetic within one year before admission date (0.0039)	Chronic Pulmonary Disease (0.0045)	Admission Source (0.0044)	Automated urinalysis, urine hemoglobin within 7 days prior to surgery, mg/dL (0.0044)	Race (0.0072)	Chronic Pulmonary Disease (0.0053)	Automated urinalysis, urine glucose within 7 days prior to surgery, mg/dL (0.005)
Automated urinalysis, urine glucose within 7 days prior to surgery, mg/dL (0.0051)	Cerebrovascular Disease (0.0036)	Congestive Heart Failure (0.0041)	Congestive Heart Failure (0.0037)	Indicator of receiving Vancomycin within one year before admission date (0.0042)	Automated urinalysis, urine hemoglobin within 7 days prior to surgery, mg/dL (0.0062)	Automated urinalysis, urine hemoglobin within 7 days prior to surgery, mg/dL (0.0049)	Indicator of receiving Vancomycin within one year before admission date (0.0045)
Valvular Disease (0.0049)	Chronic Pulmonary Disease (0.0033)	Peripheral Vascular Disease (0.0041)	Nonsteroidal anti-inflammatory drugs (0.0035)	Alcohol abuse or drug (0.004)	Alcohol abuse or drug (0.0053)	Cancer (0.0042)	Cerebrovascular Disease (0.0045)
Automated urinalysis, urine hemoglobin within 7 days prior to surgery, mg/dL (0.004)	Automated urinalysis, urine hemoglobin within 7 days prior to surgery, mg/dL (0.003)	Cancer (0.0034)	Valvular Disease (0.0031)	Coagulopathy (0.0038)	Indicator of receiving Beta-blockers within one year before admission date (0.0051)	Peripheral Vascular Disease (0.0042)	Coagulopathy (0.0043)
Coagulopathy (0.0038)	Valvular Disease (0.0021)	Automated urinalysis, urine hemoglobin	Indicator of receiving Aspirin within	Liver Disease (0.0034)	Congestive Heart Failure (0.005)	Race (0.0036)	Indicator of receiving Beta-blockers within

© 2022 Ren Y et al. *JAMA Network Open*.

Cardiovascular complication	Prolonged ICU stay	Neurological complication and delirium	Wound complication	Sepsis	Venous thromboembolism	Prolonged mechanical ventilation	Acute kidney injury
**Feature (importance weight)**							
		within 7 days prior to surgery, mg/dL (0.0034)	one year before admission date (0.0028)				one year before admission date (0.0041)
Indicator of receiving Beta-blockers within one year before admission date (0.0035)	Indicator of receiving Beta-blockers within one year before admission date (0.0021)	Coagulopathy (0.0032)	Indicator of receiving Diuretics within one year before admission date (0.0023)	Indicator of receiving Beta-blockers within one year before admission date (0.0032)	Valvular Disease (0.0045)	Chronic anemia (0.0036)	Automated urinalysis, urine hemoglobin within 7 days prior to surgery, mg/dL (0.0036)
Liver Disease (0.0032)	Alcohol abuse or drug (0.0017)	Race (0.0028)	Indicator of receiving ACE Inhibitors within one year before admission date (0.002)	Valvular Disease (0.003)	Indicator of receiving Vancomycin within one year before admission date (0.004)	Coagulopathy (0.0035)	Indicator of receiving Diuretics within one year before admission date (0.0033)
Indicator of receiving Aspirin within one year before admission date (0.0029)	Coagulopathy (0.0017)	Valvular Disease (0.0027)	Myocardial Infarction (0.0016)	Indicator of receiving Aspirin within one year before admission date (0.0029)	Indicator of receiving Aspirin within one year before admission date (0.0039)	Alcohol abuse or drug (0.0034)	Valvular Disease (0.0029)
Indicator of receiving Diuretics within one year before admission date (0.0021)	Nonsteroidal anti-inflammatory drugs (0.0017)	Alcohol abuse or drug (0.0027)	Indicator of receiving statin within one year before admission date (0.0016)	Indicator of receiving Diuretics within one year before admission date (0.0024)	Indicator of receiving Diuretics within one year before admission date (0.0037)	Valvular Disease (0.0027)	Indicator of receiving Aspirin within one year before admission date (0.0027)
Myocardial Infarction (0.0018)	Indicator of receiving Diuretics within one year before admission date (0.0012)	Nonsteroidal anti-inflammatory drugs (0.0021)	Indicator of receiving Aminoglycosides within one year before admission date (0.001)	Myocardial Infarction (0.0018)	Myocardial Infarction (0.0034)	Nonsteroidal anti-inflammatory drugs (0.0022)	Myocardial Infarction (0.0016)

© 2022 Ren Y et al. *JAMA Network Open*.

Cardiovascular complication	Prolonged ICU stay	Neurological complication and delirium	Wound complication	Sepsis	Venous thromboembolism	Prolonged mechanical ventilation	Acute kidney injury
**Feature (importance weight)**							
Indicator of receiving Bicarbonate within one year before admission date (0.0011)	Myocardial Infarction (0.0008)	Myocardial Infarction (0.0017)	Indicator of receiving Bicarbonate within one year before admission date (0.0009)	Indicator of receiving Bicarbonate within one year before admission date (0.0008)	Indicator of receiving Bicarbonate within one year before admission date (0.0015)	Myocardial Infarction (0.0019)	Indicator of receiving Bicarbonate within one year before admission date (0.0005)

Abbreviations: ICU, intensive care unit; ACE, Angiotensin-converting enzyme.

© 2022 Ren Y et al. *JAMA Network Open*.

eTable 9. Summary of the Contributions of Each Input Feature to Each Outcome for a Random Forest Model With 105 Input Features

**Cardiovascular complication**	**Prolonged ICU stay**	**Neurological complication and delirium**	**Wound complication**	**Sepsis**	**Venous thromboembolism**	**Prolonged mechanical ventilation**	**Acute kidney injury**
**Feature (importance weight)**							
ID of Attending Surgeon (0.0906)	Scheduled post operation location (0.2534)	ID of Attending Surgeon (0.1372)	ID of Attending Surgeon (0.0951)	Count of Serum CO2 test (0.0627)	ID of Attending Surgeon (0.0648)	Scheduled post operation location (0.1165)	ID of Attending Surgeon (0.1073)
Scheduled post operation location (0.0731)	ID of Attending Surgeon (0.1375)	Surgery Type (0.082)	ZIP Code of home address (0.0462)	ID of Attending Surgeon (0.0571)	Scheduled surgery room (0.039)	ID of Attending Surgeon (0.072)	Surgery Type (0.0547)
Scheduled surgery room (0.042)	Surgery Type (0.0816)	Scheduled surgery room (0.079)	Scheduled surgery room (0.0404)	Count of Urea nitrogen-Creatinine ratio (0.0489)	Variance of Hematocrit in blood, % (0.0367)	Surgery Type (0.0454)	Scheduled surgery room (0.048)
Surgery Type (0.0373)	Scheduled surgery room (0.0647)	Scheduled post operation location (0.0676)	Time from Admission to Surgery, days (0.0291)	Min of Serum Calcium, mmol/L (0.0462)	Count of Hematocrit in blood test (0.0339)	Min of Serum Calcium, mmol/L (0.0358)	Min of Hematocrit in blood, % (0.0368)
Age (0.0316)	Medicine or surgery admitting (0.0233)	Age (0.0278)	Body Mass Index (0.0283)	Min of Hematocrit in blood, % (0.0457)	ZIP Code of home address (0.026)	Scheduled surgery room (0.0352)	ZIP Code of home address (0.032)
Min of Hematocrit in blood, % (0.0275)	Charlson comorbidity index (0.0165)	Count of Urea nitrogen-Creatinine ratio (0.0239)	Distance of residence to hospital, km (0.0278)	Max of White Blood Cell in blood, thou/uL (0.0275)	Surgery Type (0.0252)	Average of Serum Calcium, mmol/L (0.0265)	Max of Urea nitrogen in blood, mg/dL (0.0319)
ZIP Code of home address (0.0271)	ZIP Code of home address (0.0163)	Time from Admission to Surgery, days (0.0222)	Age (0.0261)	Time from Admission to Surgery, days (0.022)	Time from Admission to Surgery, days (0.0245)	Count of Urea nitrogen-Creatinine ratio (0.0247)	Age (0.0297)
Body Mass Index (0.0238)	Time from Admission to	Medicine or surgery	Surgery Type (0.0259)	Scheduled surgery room (0.0214)	Age (0.0229)	Max of Glucose in blood, mg/dL (0.0244)	Charlson comorbidity index (0.0256)

© 2022 Ren Y et al. *JAMA Network Open*.

Cardiovascular complication	Prolonged ICU stay	Neurological complication and delirium	Wound complication	Sepsis	Venous thromboembolism	Prolonged mechanical ventilation	Acute kidney injury
Feature (importance weight)							
	Surgery, days (0.0161)	admitting (0.0212)					
Time from Admission to Surgery, days (0.0238)	Age (0.0153)	Body Mass Index (0.0209)	Min hemoglobin within 8-365 days prior to surgery, g/dl (0.0246)	Surgery Type (0.0211)	Count of White Blood Cell in blood test (0.0223)	Time from Admission to Surgery, days (0.0242)	Body Mass Index (0.0244)
Charlson comorbidity index (0.0234)	Admission Source (0.0141)	ZIP Code of home address (0.0201)	Median total income at patient residential area, USD (0.0225)	Scheduled post operation location (0.0199)	Body Mass Index (0.0212)	Count of Serum CO2 test (0.0192)	Time from Admission to Surgery, days (0.0212)
Distance of residence to hospital, km (0.0204)	Body Mass Index (0.0134)	Charlson comorbidity index (0.0183)	County of home address (0.0209)	Average of White Blood Cell in blood, thou/uL (0.0184)	Distance of residence to hospital, km (0.0207)	Count of Serum Sodium test (0.0186)	Average of hemoglobin within 7 days prior to surgery, g/dl (0.0202)
Average of Hematocrit in blood, % (0.0182)	Count of Urea nitrogen-Creatinine ratio (0.0131)	Distance of residence to hospital, km (0.0174)	Indicator of receiving pressors or inotropes within one year before admission date (0.0206)	Age (0.0183)	Scheduled post operation location (0.0191)	Count of Potassium in serum test (0.0178)	Average of Urea nitrogen in blood, mg/dL (0.0202)
Median total income at patient residential area, USD (0.018)	Distance of residence to hospital, km (0.0129)	Count of Potassium in serum test (0.0173)	Average of hemoglobin within 8-365 days prior to surgery, g/dl (0.0204)	Body Mass Index (0.0179)	Median total income at patient residential area, USD (0.0184)	Admission Source (0.0157)	Average of hemoglobin within 8-365 days prior to surgery, g/dl (0.0188)
Min of Serum Calcium,	Min of Serum Calcium,	Count of Serum CO2 test (0.0144)	Average of hemoglobin within 7 days	ZIP Code of home address (0.0167)	Min of Hematocrit in	ZIP Code of home address (0.0155)	Distance of residence to

© 2022 Ren Y et al. *JAMA Network Open*.

Cardiovascular complication	Prolonged ICU stay	Neurological complication and delirium	Wound complication	Sepsis	Venous thromboembolism	Prolonged mechanical ventilation	Acute kidney injury
Feature (importance weight)							
mmol/L (0.0179)	mmol/L (0.0123)		prior to surgery, g/dl (0.0169)		blood, % (0.017)		hospital, km (0.0185)
Max of Urea nitrogen in blood, mg/dL (0.0166)	Max of Glucose in blood, mg/dL (0.011)	Max of Glucose in blood, mg/dL (0.0134)	Average of Hematocrit in blood, % (0.0165)	Emergent or elective admission (0.0163)	Charlson comorbidity index (0.0156)	Age (0.0151)	Average of Hematocrit in blood, % (0.0184)
Average of hemoglobin within 7 days prior to surgery, g/dl (0.0151)	Count of Glucose in blood test (0.011)	Count of Serum Sodium test (0.0132)	Min of Hematocrit in blood, % (0.0163)	Average of hemoglobin within 7 days prior to surgery, g/dl (0.0158)	Count of Serum Sodium test (0.0151)	Medicine or surgery admitting (0.015)	Median total income at patient residential area, USD (0.0174)
Average of hemoglobin within 8-365 days prior to surgery, g/dl (0.0143)	Average of Serum Calcium, mmol/L (0.0109)	Variance of Urea nitrogen-Creatinine ratio (0.0132)	Admission Month (0.0158)	Count of Serum Sodium test (0.0152)	County of home address (0.0148)	Average of Glucose in blood, mg/dL (0.0143)	Min hemoglobin within 8-365 days prior to surgery, g/dl (0.0161)
County of home address (0.0136)	Min of Hematocrit in blood, % (0.0096)	Average of Serum Calcium, mmol/L (0.0126)	Variance of hemoglobin within 8-365 days prior to surgery, g/dl (0.0146)	Variance of Hematocrit in blood, % (0.015)	Average of Red cell distribution width in Blood, % (0.0146)	Charlson comorbidity index (0.0141)	Scheduled post operation location (0.0143)
Average of Urea nitrogen in blood, mg/dL (0.0128)	Count of Potassium in serum test (0.0086)	Count of Glucose in blood test (0.0121)	Max hemoglobin within 8-365 days prior to surgery, g/dl (0.0146)	Distance of residence to hospital, km (0.0145)	Number of hemoglobin tests within 8-365 days prior to surgery (0.0136)	Distance of residence to hospital, km (0.0138)	Count of Urea nitrogen-Creatinine ratio (0.0132)
Average of Serum Calcium, mmol/L (0.0127)	Average of Glucose in blood, mg/dL (0.0084)	County of home address (0.0121)	Charlson comorbidity index (0.0145)	Charlson comorbidity index (0.0144)	Average of hemoglobin within 8-365 days prior to	Max of White Blood Cell in blood, thou/uL (0.0126)	County of home address (0.0123)

© 2022 Ren Y et al. *JAMA Network Open*.

Cardiovascular complication	Prolonged ICU stay	Neurological complication and delirium	Wound complication	Sepsis	Venous thromboembolism	Prolonged mechanical ventilation	Acute kidney injury
Feature (importance weight)							
					surgery, g/dl (0.0132)		
Count of Urea nitrogen-Creatinine ratio (0.0126)	County of home address (0.0084)	Average of hemoglobin within 8-365 days prior to surgery, g/dl (0.0121)	Number of hemoglobin tests within 8-365 days prior to surgery (0.0136)	Median total income at patient residential area, USD (0.0143)	Variance of Urea nitrogen-Creatinine ratio (0.0124)	Min of Hematocrit in blood, % (0.0126)	Max of Glucose in blood, mg/dL (0.0121)
Max hemoglobin within 8-365 days prior to surgery, g/dl (0.0125)	Average of hemoglobin within 8-365 days prior to surgery, g/dl (0.008)	Max of Urea nitrogen-Creatinine ratio (0.0118)	Average of Glucose in blood, mg/dL (0.0122)	Variance of Urea nitrogen-Creatinine ratio (0.0131)	Max hemoglobin within 8-365 days prior to surgery, g/dl (0.0124)	Variance of Hematocrit in blood, % (0.0118)	Count of Glucose in blood test (0.012)
Insurance paying the bills (0.0125)	Max of White Blood Cell in blood, thou/uL (0.0076)	Min of Serum Calcium, mmol/L (0.0114)	Average of Mean Corpuscular Volume in blood, fL (0.0122)	Average of Hematocrit in blood, % (0.0127)	Max of Red cell distribution width in Blood, % (0.0124)	Count of Hematocrit in blood test (0.0118)	Max hemoglobin within 8-365 days prior to surgery, g/dl (0.0119)
Min hemoglobin within 8-365 days prior to surgery, g/dl (0.0123)	Average of White Blood Cell in blood, thou/uL (0.0074)	Admission Source (0.0113)	Max of Hematocrit in blood, % (0.0119)	Count of Hematocrit in blood test (0.0116)	Count of Urea nitrogen-Creatinine ratio (0.0124)	Min of Glucose in blood, mg/dL (0.0117)	Number of hemoglobin tests within 8-365 days prior to surgery (0.0112)
Admission Month (0.0118)	Max of Urea nitrogen-Creatinine ratio (0.0074)	Average of Urea nitrogen-Creatinine ratio (0.011)	Min of Glucose in blood, mg/dL (0.0117)	Number of hemoglobin tests within 8-365 days prior to surgery (0.0114)	Min hemoglobin within 8-365 days prior to surgery, g/dl (0.0124)	County of home address (0.011)	Admission Month (0.0109)
Average of Mean Corpuscular	Min of Glucose in blood, mg/dL (0.0071)	Average of Glucose in	Max of Glucose in blood, mg/dL (0.0117)	Count of Urea nitrogen in	Min of Red cell distribution	Average of White Blood	Average of Glucose in

© 2022 Ren Y et al. *JAMA Network Open*.

Cardiovascular complication	Prolonged ICU stay	Neurological complication and delirium	Wound complication	Sepsis	Venous thromboembolism	Prolonged mechanical ventilation	Acute kidney injury
**Feature (importance weight)**							
Volume in blood, fL (0.0116)		blood, mg/dL (0.0109)		blood test (0.0114)	width in Blood, % (0.0122)	Cell in blood, thou/uL (0.011)	blood, mg/dL (0.0108)
Max of Glucose in blood, mg/dL (0.0106)	Min hemoglobin within 8-365 days prior to surgery, g/dl (0.007)	Min hemoglobin within 8-365 days prior to surgery, g/dl (0.0109)	Average of The amount of hemoglobin relative to the size of the cell in blood, g/dL (0.0116)	Average of Serum Sodium, mmol/L (0.0112)	Average of Hematocrit in blood, % (0.0121)	Variance of Urea nitrogen-Creatinine ratio (0.0108)	Min of Glucose in blood, mg/dL (0.0103)
Average of Glucose in blood, mg/dL (0.0101)	Average of Urea nitrogen-Creatinine ratio (0.0067)	Average of platelet in blood, thou/uL (0.0108)	Average of Mean platelet volume in blood, fL (0.0112)	Average of Serum Calcium, mmol/L (0.0112)	Count of Potassium in serum test (0.0121)	Average of hemoglobin within 8-365 days prior to surgery, g/dl (0.0104)	Min of Urea nitrogen in blood, mg/dL (0.0099)
Min of Glucose in blood, mg/dL (0.0099)	Min of Urea nitrogen-Creatinine ratio (0.0062)	Min of Glucose in blood, mg/dL (0.0105)	Insurance paying the bills (0.011)	Average of hemoglobin within 8-365 days prior to surgery, g/dl (0.0112)	Max of Glucose in blood, mg/dL (0.012)	Min of Serum CO2 , mmol/L (0.0102)	Average of Serum Calcium, mmol/L (0.0095)
Average of platelet in blood, thou/uL (0.0099)	Number of hemoglobin tests within 8-365 days prior to surgery (0.006)	Average of Mean platelet volume in blood, fL (0.0104)	Average of Red cell distribution width in Blood, % (0.0109)	County of home address (0.0101)	Variance of hemoglobin within 8-365 days prior to surgery, g/dl (0.012)	Max of Urea nitrogen-Creatinine ratio (0.0098)	Average of The amount of hemoglobin relative to the size of the cell in blood, g/dL (0.0095)
Max of Urea nitrogen-Creatinine ratio (0.0096)	Average of platelet in blood, thou/uL (0.0058)	Min of Urea nitrogen-Creatinine ratio (0.0101)	Average of platelet in blood, thou/uL (0.0107)	Min hemoglobin within 8-365 days prior to surgery, g/dl (0.0097)	Average of Glucose in blood, mg/dL (0.0119)	Min hemoglobin within 8-365 days prior to surgery, g/dl (0.0097)	Average of Mean Corpuscular Volume in blood, fL (0.0093)

© 2022 Ren Y et al. *JAMA Network Open*.

Cardiovascular complication	Prolonged ICU stay	Neurological complication and delirium	Wound complication	Sepsis	Venous thromboembolism	Prolonged mechanical ventilation	Acute kidney injury
**Feature (importance weight)**							
Average of Mean platelet volume in blood, fL (0.0095)	Average of Mean Corpuscular Volume in blood, fL (0.0057)	Max of White Blood Cell in blood, thou/uL (0.0095)	Max of platelet in blood, thou/uL (0.0106)	Average of The amount of hemoglobin relative to the size of the cell in blood, g/dL (0.0096)	Average of The amount of hemoglobin relative to the size of the cell in blood, g/dL (0.0116)	Average of Urea nitrogen-Creatinine ratio (0.0091)	Variance of hemoglobin within 8-365 days prior to surgery, g/dl (0.0093)
Average of The amount of hemoglobin relative to the size of the cell in blood, g/dL (0.0093)	Variance of Urea nitrogen-Creatinine ratio (0.0056)	Average of White Blood Cell in blood, thou/uL (0.0095)	Min of Mean platelet volume in blood, fL (0.0102)	Max hemoglobin within 8-365 days prior to surgery, g/dl (0.0096)	Admission Month (0.0115)	Average of Serum CO2, mmol/L (0.009)	Min of Serum Calcium, mmol/L (0.0092)
Average of Urea nitrogen-Creatinine ratio (0.0092)	Average of Hematocrit in blood, % (0.0055)	Number of hemoglobin tests within 8-365 days prior to surgery (0.0092)	Max hemoglobin within 7 days prior to surgery, g/dl (0.01)	Min of Glucose in blood, mg/dL (0.0095)	Count of Serum CO2 test (0.0114)	Count of Urea nitrogen in blood test (0.0088)	Variance of Urea nitrogen-Creatinine ratio (0.0091)
Average of Red cell distribution width in Blood, % (0.009)	Count of Serum Sodium test (0.0055)	Emergent or elective admission (0.0092)	Min of Red cell distribution width in Blood, % (0.0099)	Average of Glucose in blood, mg/dL (0.0094)	Average of hemoglobin within 7 days prior to surgery, g/dl (0.0112)	Max of Serum Sodium, mmol/L (0.0087)	Min of Urea nitrogen-Creatinine ratio (0.009)
Max of platelet in blood, thou/uL (0.009)	Average of Mean platelet volume in blood, fL (0.0055)	Min of Hematocrit in blood, % (0.009)	Average of White Blood Cell in blood, thou/uL (0.0099)	Average of Red cell distribution width in Blood, % (0.0094)	Average of Mean Corpuscular Volume in blood, fL (0.0111)	Average of hemoglobin within 7 days prior to surgery, g/dl (0.0086)	Average of White Blood Cell in blood, thou/uL (0.0089)
Number of hemoglobin tests within 8-365 days prior	Count of Serum CO2 test (0.0055)	Average of Serum CO2, mmol/L (0.0087)	Max of White Blood Cell in blood, thou/uL (0.0098)	Average of Mean Corpuscular Volume in	Min of Glucose in blood, mg/dL (0.0111)	Count of Glucose in blood test (0.0085)	Average of Urea nitrogen-Creatinine ratio (0.0088)

© 2022 Ren Y et al. *JAMA Network Open*.

Cardiovascular complication	Prolonged ICU stay	Neurological complication and delirium	Wound complication	Sepsis	Venous thromboembolism	Prolonged mechanical ventilation	Acute kidney injury
**Feature (importance weight)**							
to surgery (0.009)				blood, fL (0.0093)			
Variance of hemoglobin within 8-365 days prior to surgery, g/dl (0.0089)	Average of hemoglobin within 7 days prior to surgery, g/dl (0.0054)	Variance of Hematocrit in blood, % (0.0085)	Max of Red cell distribution width in Blood, % (0.0097)	Max of Glucose in blood, mg/dL (0.0093)	Emergent or elective admission (0.01)	Admission Month (0.0085)	Max of White Blood Cell in blood, thou/uL (0.0087)
Min of Urea nitrogen-Creatinine ratio (0.0087)	Average of Urea nitrogen in blood, mg/dL (0.0053)	Average of anion gap in blood, mmol/L (0.0081)	Max of Urea nitrogen-Creatinine ratio (0.0096)	Admission Month (0.0089)	Average of White Blood Cell in blood, thou/uL (0.0099)	Min of Urea nitrogen-Creatinine ratio (0.0084)	Max of Urea nitrogen-Creatinine ratio (0.0085)
Min of Mean platelet volume in blood, fL (0.0081)	Average of anion gap in blood, mmol/L (0.0053)	Average of Red cell distribution width in Blood, % (0.0078)	Average of Potassium in serum, mmol/L (0.0095)	Admission Source (0.0088)	Average of Serum Calcium, mmol/L (0.0099)	Average of anion gap in blood, mmol/L (0.0084)	Insurance paying the bills (0.0082)
Average of White Blood Cell in blood, thou/uL (0.0081)	Average of The amount of hemoglobin relative to the size of the cell in blood, g/dL (0.0051)	Average of hemoglobin within 7 days prior to surgery, g/dl (0.0078)	Min of Urea nitrogen-Creatinine ratio (0.0094)	Variance of hemoglobin within 8-365 days prior to surgery, g/dl (0.0085)	Max of White Blood Cell in blood, thou/uL (0.0098)	Average of platelet in blood, thou/uL (0.0083)	Average of Red cell distribution width in Blood, % (0.0082)
Max of White Blood Cell in blood, thou/uL (0.0081)	Average of Red cell distribution width in Blood, % (0.0051)	Max of Hematocrit in blood, % (0.0077)	Average of Urea nitrogen-Creatinine ratio (0.0094)	Min of Serum Sodium, mmol/L (0.0085)	Max of Hematocrit in blood, % (0.0094)	Average of Hematocrit in blood, % (0.0083)	Average of platelet in blood, thou/uL (0.008)
Max of Red cell distribution width in Blood, % (0.008)	Variance of Glucose in blood, mg/dL (0.0051)	Min of Serum CO2 , mmol/L (0.0077)	Average of Serum Calcium, mmol/L (0.0094)	Max of Red cell distribution width in Blood, % (0.0082)	Average of Urea nitrogen-Creatinine ratio (0.0093)	Average of Red cell distribution width in Blood, % (0.0083)	Average of Potassium in serum, mmol/L (0.0077)

© 2022 Ren Y et al. *JAMA Network Open*.

Cardiovascular complication	Prolonged ICU stay	Neurological complication and delirium	Wound complication	Sepsis	Venous thromboembolism	Prolonged mechanical ventilation	Acute kidney injury
**Feature (importance weight)**							
Min of Red cell distribution width in Blood, % (0.008)	Min of Red cell distribution width in Blood, % (0.0048)	Average of Hematocrit in blood, % (0.0077)	Variance of Urea nitrogen-Creatinine ratio (0.0091)	Min of Red cell distribution width in Blood, % (0.0081)	Max of Urea nitrogen-Creatinine ratio (0.0093)	Min of Red cell distribution width in Blood, % (0.0081)	Max of platelet in blood, thou/uL (0.0076)
Min of Urea nitrogen in blood, mg/dL (0.0077)	Average of Potassium in serum, mmol/L (0.0046)	Insurance paying the bills (0.0076)	Average of Urea nitrogen in blood, mg/dL (0.0081)	Average of Urea nitrogen in blood, mg/dL (0.0079)	Average of platelet in blood, thou/uL (0.0091)	Average of The amount of hemoglobin relative to the size of the cell in blood, g/dL (0.0079)	Average of Mean platelet volume in blood, fL (0.0075)
Variance of Urea nitrogen-Creatinine ratio (0.0077)	Max of Hematocrit in blood, % (0.0046)	Min of Red cell distribution width in Blood, % (0.0073)	Average of anion gap in blood, mmol/L (0.0079)	Average of platelet in blood, thou/uL (0.0078)	Average of Mean platelet volume in blood, fL (0.009)	Average of Mean Corpuscular Volume in blood, fL (0.0076)	Min of Red cell distribution width in Blood, % (0.0073)
Max of Hematocrit in blood, % (0.0076)	Yes if the admission we happened at night (0.0045)	Average of Urea nitrogen in blood, mg/dL (0.0069)	Min of Serum Calcium, mmol/L (0.0075)	Max of Urea nitrogen-Creatinine ratio (0.0077)	Average of Potassium in serum, mmol/L (0.009)	Max of Red cell distribution width in Blood, % (0.0075)	Max of Red cell distribution width in Blood, % (0.0071)
Count of Potassium in serum test (0.0076)	Max of Urea nitrogen in blood, mg/dL (0.0045)	Max of Red cell distribution width in Blood, % (0.0066)	Average of Serum CO2, mmol/L (0.0071)	Average of Mean platelet volume in blood, fL (0.0076)	Min of Urea nitrogen-Creatinine ratio (0.0089)	Average of Urea nitrogen in blood, mg/dL (0.0075)	Max of Hematocrit in blood, % (0.007)
Average of Potassium in serum, mmol/L (0.0076)	Average of Serum CO2, mmol/L (0.0044)	Max of Serum Sodium, mmol/L (0.0066)	Emergent or elective admission (0.007)	Min of Urea nitrogen-Creatinine ratio (0.0075)	Max of platelet in blood, thou/uL (0.0087)	Count of anion gap in blood test (0.0075)	Count of Serum Sodium test (0.0069)
Average of anion gap in blood, mmol/L (0.0073)	Max of Red cell distribution width in Blood, % (0.0044)	Variance of Glucose in blood, mg/dL (0.006)	Max of Urea nitrogen in blood, mg/dL (0.0069)	Max of platelet in blood, thou/uL (0.0075)	Variance of Glucose in blood, mg/dL (0.0083)	Variance of hemoglobin within 8-365 days prior to	Variance of Glucose in blood, mg/dL (0.0068)

© 2022 Ren Y et al. *JAMA Network Open*.

Cardiovascular complication	Prolonged ICU stay	Neurological complication and delirium	Wound complication	Sepsis	Venous thromboembolism	Prolonged mechanical ventilation	Acute kidney injury
**Feature (importance weight)**							
						surgery, g/dl (0.0072)	
Medicine or surgery admitting (0.0071)	Variance of Hematocrit in blood, % (0.0042)	Max of Urea nitrogen in blood, mg/dL (0.0059)	Smoking Status (0.0066)	Average of Urea nitrogen-Creatinine ratio (0.0075)	Average of Urea nitrogen in blood, mg/dL (0.0079)	Max of Hematocrit in blood, % (0.0072)	Average of Serum CO2, mmol/L (0.0065)
Average of Serum CO2, mmol/L (0.0068)	Average of Serum Sodium, mmol/L (0.0041)	Count of Urea nitrogen in blood test (0.0057)	Indicator of receiving Antiemetic within one year before admission date (0.0065)	Max of Urea nitrogen in blood, mg/dL (0.0073)	Min of Mean platelet volume in blood, fL (0.0078)	Number of hemoglobin tests within 8-365 days prior to surgery (0.0072)	Average of anion gap in blood, mmol/L (0.0064)
Max hemoglobin within 7 days prior to surgery, g/dl (0.0066)	Count of Hematocrit in blood test (0.0041)	Count of Hematocrit in blood test (0.0053)	Average of Serum Sodium, mmol/L (0.0064)	Max of Hematocrit in blood, % (0.007)	Min of Serum Calcium, mmol/L (0.0077)	Variance of Glucose in blood, mg/dL (0.0072)	Min of Mean platelet volume in blood, fL (0.0063)
Min of Serum CO2 , mmol/L (0.0064)	Min of Urea nitrogen in blood, mg/dL (0.004)	Min of Serum Sodium, mmol/L (0.0053)	Min of Urea nitrogen in blood, mg/dL (0.0063)	Average of Potassium in serum, mmol/L (0.0068)	Max hemoglobin within 7 days prior to surgery, g/dl (0.0072)	Average of Serum Sodium, mmol/L (0.0069)	Max hemoglobin within 7 days prior to surgery, g/dl (0.0063)
Average of Serum Sodium, mmol/L (0.0063)	Min of Serum CO2 , mmol/L (0.0039)	Min of Urea nitrogen in blood, mg/dL (0.0053)	Variance of Glucose in blood, mg/dL (0.006)	Count of anion gap in blood test (0.0067)	Average of anion gap in blood, mmol/L (0.0071)	Average of Mean platelet volume in blood, fL (0.0067)	CKD status at admission (0.0058)
Variance of Glucose in blood, mg/dL (0.0061)	Max hemoglobin within 7 days prior to surgery, g/dl (0.0038)	Smoking Status (0.0044)	Min of anion gap in blood, mmol/L (0.0054)	Average of Serum CO2, mmol/L (0.0067)	Average of Serum Sodium, mmol/L (0.007)	Min of Mean platelet volume in blood, fL (0.0066)	Average of Serum Sodium, mmol/L (0.0057)

© 2022 Ren Y et al. *JAMA Network Open*.

Cardiovascular complication	Prolonged ICU stay	Neurological complication and delirium	Wound complication	Sepsis	Venous thromboembolism	Prolonged mechanical ventilation	Acute kidney injury
**Feature (importance weight)**							
Variance of Hematocrit in blood, % (0.0057)	Insurance paying the bills (0.0033)	Variance of hemoglobin within 7 days prior to surgery, g/dl (0.0043)	Min of Serum CO2 , mmol/L (0.0054)	Max hemoglobin within 7 days prior to surgery, g/dl (0.0067)	Average of Serum CO2, mmol/L (0.0066)	Variance of hemoglobin within 7 days prior to surgery, g/dl (0.0065)	Count of Potassium in serum test (0.0055)
Count of Glucose in blood test (0.0054)	Max of Serum Sodium, mmol/L (0.0032)	Variance of Urea nitrogen in blood, mg/dL (0.0039)	Medicine or surgery admitting (0.0052)	Min of Mean platelet volume in blood, fL (0.0066)	Min of Urea nitrogen in blood, mg/dL (0.0064)	Max of Urea nitrogen in blood, mg/dL (0.0064)	Min of Serum CO2 , mmol/L (0.0052)
Count of Serum Sodium test (0.0053)	Smoking Status (0.003)	Variance of platelet in blood, thou/uL (0.0038)	Yes if the admission we happened at night (0.005)	Variance of Glucose in blood, mg/dL (0.0062)	Count of Glucose in blood test (0.0063)	Min of Urea nitrogen in blood, mg/dL (0.0064)	Smoking Status (0.0049)
Count of Serum CO2 test (0.0052)	Min of Serum Sodium, mmol/L (0.0029)	Variance of Serum Calcium, mmol/L (0.0036)	Number of urine glucose tests within 8-365 days prior to surgery (0.0049)	Average of anion gap in blood, mmol/L (0.0061)	Max of Urea nitrogen in blood, mg/dL (0.0062)	Max hemoglobin within 7 days prior to surgery, g/dl (0.0062)	Min of Serum Sodium, mmol/L (0.0046)
Max of Serum Sodium, mmol/L (0.0049)	Variance of hemoglobin within 7 days prior to surgery, g/dl (0.0027)	Variance of Serum CO2, mmol/L (0.0034)	Max of Serum CO2, mmol/L (0.0048)	Min of Serum CO2 , mmol/L (0.0056)	Medicine or surgery admitting (0.0061)	Smoking Status (0.006)	Count of Serum CO2 test (0.004)
Smoking Status (0.0048)	Number of urine glucose tests within 0-7 days prior to surgery (0.0026)	Variance of Serum Sodium, mmol/L (0.0034)	Count of Urea nitrogen-Creatinine ratio (0.0048)	Min of Urea nitrogen in blood, mg/dL (0.0056)	Count of Urea nitrogen in blood test (0.006)	Max of Serum CO2, mmol/L (0.0054)	Max of Serum Sodium, mmol/L (0.004)
Min of Serum Sodium, mmol/L (0.0047)	Variance of Urea nitrogen in blood, mg/dL (0.0024)	Marital Status (0.0034)	Min of Serum Sodium, mmol/L (0.0046)	Max of Serum Sodium, mmol/L (0.0055)	Variance of hemoglobin within 7 days prior to surgery, g/dl (0.0057)	Variance of Urea nitrogen in blood, mg/dL (0.0051)	Max of Serum CO2, mmol/L (0.0039)

© 2022 Ren Y et al. *JAMA Network Open*.

Cardiovascular complication	Prolonged ICU stay	Neurological complication and delirium	Wound complication	Sepsis	Venous thromboembolism	Prolonged mechanical ventilation	Acute kidney injury
**Feature (importance weight)**							
Variance of Urea nitrogen in blood, mg/dL (0.0046)	Count of Urea nitrogen in blood test (0.0023)	Count of anion gap in blood test (0.0033)	Max of Serum Sodium, mmol/L (0.0045)	Count of Potassium in serum test (0.0055)	Max of Serum Sodium, mmol/L (0.0055)	Insurance paying the bills (0.0044)	Min of anion gap in blood, mmol/L (0.0039)
Min of anion gap in blood, mmol/L (0.0045)	Variance of Serum CO2, mmol/L (0.0022)	Count of The amount of hemoglobin relative to the size of the cell in blood test (0.0032)	Count of Glucose in blood test (0.0044)	Insurance paying the bills (0.0052)	Variance of platelet in blood, thou/uL (0.0055)	Min of Serum Sodium, mmol/L (0.0042)	Count of Urea nitrogen in blood test (0.0038)
Count of Hematocrit in blood test (0.0045)	Variance of Serum Sodium, mmol/L (0.002)	Anesthesia Type (0.0029)	Marital Status (0.0043)	Count of Glucose in blood test (0.0048)	Admission Source (0.0053)	Variance of Serum Sodium, mmol/L (0.004)	Variance of Urea nitrogen in blood, mg/dL (0.0037)
Max of Serum CO2, mmol/L (0.0043)	CKD status at admission (0.002)	Count of White Blood Cell in blood test (0.0025)	Indicator of receiving Beta-blockers within one year before admission date (0.0043)	Medicine or surgery admitting (0.0046)	Variance of Urea nitrogen in blood, mg/dL (0.0052)	Variance of Serum CO2, mmol/L (0.0038)	Number of urine glucose tests within 8-365 days prior to surgery (0.0037)
Variance of Serum CO2, mmol/L (0.004)	Variance of Serum Calcium, mmol/L (0.002)	Sex (0.0023)	Variance of Serum CO2, mmol/L (0.0043)	Variance of platelet in blood, thou/uL (0.0045)	Count of The amount of hemoglobin relative to the size of the cell in blood test (0.0051)	Count of The amount of hemoglobin relative to the size of the cell in blood test (0.0035)	Variance of Hematocrit in blood, % (0.0036)
Variance of hemoglobin within 7 days prior to surgery, g/dl (0.004)	Count of The amount of hemoglobin relative to the size of the cell in blood test (0.0019)	CKD status at admission (0.0023)	Anesthesia Type (0.0043)	Variance of Urea nitrogen in blood, mg/dL (0.0042)	Min of anion gap in blood, mmol/L (0.005)	Emergent or elective admission (0.0034)	Variance of hemoglobin within 7 days prior to surgery, g/dl (0.0035)

© 2022 Ren Y et al. *JAMA Network Open*.

Cardiovascular complication	Prolonged ICU stay	Neurological complication and delirium	Wound complication	Sepsis	Venous thromboembolism	Prolonged mechanical ventilation	Acute kidney injury
**Feature (importance weight)**							
Marital Status (0.0035)	Variance of platelet in blood, thou/uL (0.0019)	Indicator of receiving Antiemetic within one year before admission date (0.0017)	Variance of Hematocrit in blood, % (0.0043)	Max of Serum CO2, mmol/L (0.0041)	Min of Serum Sodium, mmol/L (0.0049)	Variance of Serum Calcium, mmol/L (0.0033)	Variance of platelet in blood, thou/uL (0.0035)
Number of urine glucose tests within 8-365 days prior to surgery (0.0033)	Sex (0.0019)	Indicator of receiving pressors or inotropes within one year before admission date (0.0015)	Variance of hemoglobin within 7 days prior to surgery, g/dl (0.0042)	Variance of hemoglobin within 7 days prior to surgery, g/dl (0.0041)	Min of Serum CO2 , mmol/L (0.0048)	CKD status at admission (0.0033)	Variance of Serum Sodium, mmol/L (0.0035)
Variance of platelet in blood, thou/uL (0.0032)	Emergent or elective admission (0.0018)	Number of urine glucose tests within 0-7 days prior to surgery (0.0012)	Scheduled post operation location (0.0041)	CKD status at admission (0.004)	Variance of Serum Sodium, mmol/L (0.0047)	Count of White Blood Cell in blood test (0.0032)	Variance of Serum CO2, mmol/L (0.0035)
Variance of Serum Sodium, mmol/L (0.0032)	Marital Status (0.0018)	Race (0.0012)	Variance of platelet in blood, thou/uL (0.0037)	Variance of Serum CO2, mmol/L (0.0039)	Count of anion gap in blood test (0.0047)	Marital Status (0.0032)	Medicine or surgery admitting (0.0035)
Variance of Serum Calcium, mmol/L (0.0031)	Count of White Blood Cell in blood test (0.0015)	Count of Serum Calcium test (0.0012)	Sex (0.0035)	Min of anion gap in blood, mmol/L (0.0039)	Variance of Serum Calcium, mmol/L (0.0046)	Variance of platelet in blood, thou/uL (0.0031)	Variance of Serum Calcium, mmol/L (0.0034)
Yes if the admission we happened at night (0.0029)	Count of anion gap in blood test (0.0014)	Scheduled room is trauma room or not (0.0005)	Variance of Serum Sodium, mmol/L (0.0035)	Variance of Serum Sodium, mmol/L (0.0035)	Max of Serum CO2, mmol/L (0.0045)	Count of Serum Calcium test (0.0028)	Marital Status (0.0032)

© 2022 Ren Y et al. *JAMA Network Open*.

Cardiovascular complication	Prolonged ICU stay	Neurological complication and delirium	Wound complication	Sepsis	Venous thromboembolism	Prolonged mechanical ventilation	Acute kidney injury
**Feature (importance weight)**							
CKD status at admission (0.0028)	Rural or city at patient residential area (0.0012)		Count of The amount of hemoglobin relative to the size of the cell in blood test (0.0033)	Variance of Serum Calcium, mmol/L (0.0033)	Variance of Serum CO2, mmol/L (0.0043)	Yes if the admission we happened at night (0.0023)	Yes if the admission we happened at night (0.0026)
Count of Urea nitrogen in blood test (0.0028)	Race (0.001)		Variance of Serum Calcium, mmol/L (0.0031)	Smoking Status (0.0031)	Insurance paying the bills (0.0042)	Sex (0.002)	Emergent or elective admission (0.0024)
Sex (0.0027)	Indicator of receiving Antiemetic within one year before admission date (0.001)		Number of urine hemoglobin tests within 8-365 days prior to surgery (0.0031)	Number of urine glucose tests within 8-365 days prior to surgery (0.0028)	Number of urine glucose tests within 8-365 days prior to surgery (0.0042)	Number of urine hemoglobin tests within 8-365 days prior to surgery (0.0017)	Sex (0.0023)
Count of anion gap in blood test (0.0027)	Number of urine hemoglobin tests within 8-365 days prior to surgery (0.001)		Variance of Urea nitrogen in blood, mg/dL (0.003)	Marital Status (0.0026)	Smoking Status (0.0039)	Number of urine glucose tests within 0-7 days prior to surgery (0.0017)	Rural or city at patient residential area (0.0021)
Count of The amount of hemoglobin relative to the size of the cell in blood test (0.0026)	Indicator of receiving pressors or inotropes within one year before admission date (0.0008)		CKD status at admission (0.0026)	Sex (0.0022)	Marital Status (0.0036)	Indicator of receiving Antiemetic within one year before admission date (0.0017)	Count of The amount of hemoglobin relative to the size of the cell in blood test (0.0021)
Count of White Blood Cell in	Anesthesia Type (0.0008)		Rural or city at patient	Count of The amount of hemoglobin	CKD status at admission (0.0029)	Automated urinalysis, urine glucose within	Count of Serum Calcium test (0.0019)

© 2022 Ren Y et al. *JAMA Network Open*.

Cardiovascular complication	Prolonged ICU stay	Neurological complication and delirium	Wound complication	Sepsis	Venous thromboembolism	Prolonged mechanical ventilation	Acute kidney injury
**Feature (importance weight)**							
blood test (0.0025)			residential area (0.0026)	relative to the size of the cell in blood test (0.0022)		8-365 days prior to surgery, mg/dL (0.0016)	
Emergent or elective admission (0.0022)	Count of Serum Calcium test (0.0008)		Race (0.0022)	Count of White Blood Cell in blood test (0.002)	Number of urine hemoglobin tests within 8-365 days prior to surgery (0.0024)	Rural or city at patient residential area (0.0016)	Indicator of receiving Beta-blockers within one year before admission date (0.0019)
Rural or city at patient residential area (0.0021)	Automated urinalysis, urine hemoglobin within 8-365 days prior to surgery, mg/dL (0.0007)		Count of Hematocrit in blood test (0.0021)	Number of urine glucose tests within 0-7 days prior to surgery (0.002)	Rural or city at patient residential area (0.0024)	Scheduled room is trauma room or not (0.0016)	Admission Source (0.0017)
Number of urine hemoglobin tests within 8-365 days prior to surgery (0.0017)	Indicator of receiving Beta-blockers within one year before admission date (0.0006)		Automated urinalysis, urine hemoglobin within 8-365 days prior to surgery, mg/dL (0.0018)	Yes if the admission we happened at night (0.0018)	Yes if the admission we happened at night (0.0023)	Race (0.0014)	Automated urinalysis, urine glucose within 8-365 days prior to surgery, mg/dL (0.0017)
Admission Source (0.0017)	Indicator of receiving Diuretics within one year before admission date (0.0004)		Automated urinalysis, urine glucose within 8-365 days prior to surgery, mg/dL (0.0017)	Rural or city at patient residential area (0.0018)	Number of urine glucose tests within 0-7 days prior to surgery (0.0023)	Indicator of receiving pressors or inotropes within one year before admission date (0.0014)	Count of Hematocrit in blood test (0.0016)
Race (0.0016)	Indicator of receiving ACE Inhibitors within one year before		Count of White Blood Cell in blood test (0.0017)	Number of urine hemoglobin tests within 8-	Sex (0.0022)	Automated urinalysis, urine hemoglobin within 8-365	Race (0.0015)

© 2022 Ren Y et al. *JAMA Network Open*.

**Cardiovascular complication**	**Prolonged ICU stay**	**Neurological complication and delirium**	**Wound complication**	**Sepsis**	**Venous thromboembolism**	**Prolonged mechanical ventilation**	**Acute kidney injury**
**Feature (importance weight)**							
	admission date (0.0004)			365 days prior to surgery (0.0015)		days prior to surgery, mg/dL (0.0011)	
Indicator of receiving Antiemetic within one year before admission date (0.0014)	Indicator of receiving statin within one year before admission date (0.0003)		Count of Urea nitrogen in blood test (0.0015)	Indicator of receiving pressors or inotropes within one year before admission date (0.0014)	Count of Serum Calcium test (0.0021)	Anesthesia Type (0.001)	Indicator of receiving Antiemetic within one year before admission date (0.0015)
Automated urinalysis, urine glucose within 8-365 days prior to surgery, mg/dL (0.0013)	Ethnicity (0.0002)		Admission Source (0.0014)	Count of Serum Calcium test (0.0014)	Race (0.002)	Indicator of receiving Diuretics within one year before admission date (0.0007)	Automated urinalysis, urine hemoglobin within 8-365 days prior to surgery, mg/dL (0.0014)
Automated urinalysis, urine hemoglobin within 8-365 days prior to surgery, mg/dL (0.0013)	Indicator of receiving Bicarbonate within one year before admission date (0.0002)		Count of Potassium in serum test (0.0014)	Race (0.0013)	Automated urinalysis, urine hemoglobin within 8-365 days prior to surgery, mg/dL (0.0015)	Native Language Spoken (0.0005)	Number of urine hemoglobin tests within 8-365 days prior to surgery (0.0014)
Count of Serum Calcium test (0.0013)	Scheduled room is trauma room or not (0.0002)		Count of anion gap in blood test (0.0013)	Indicator of receiving Antiemetic within one year before admission date (0.0012)	Automated urinalysis, urine glucose within 8-365 days prior to surgery, mg/dL (0.0015)	Ethnicity (0.0004)	Count of anion gap in blood test (0.0014)
Number of urine glucose tests within 0-7 days prior to			Number of urine glucose tests within 0-7 days prior to	Automated urinalysis, urine glucose within 8-365 days	Indicator of receiving pressors or inotropes within one year before		Indicator of receiving Diuretics within one year before

© 2022 Ren Y et al. *JAMA Network Open*.

**Cardiovascular complication**	**Prolonged ICU stay**	**Neurological complication and delirium**	**Wound complication**	**Sepsis**	**Venous thromboembolism**	**Prolonged mechanical ventilation**	**Acute kidney injury**
**Feature (importance weight)**							
surgery (0.0012)			surgery (0.0013)	prior to surgery, mg/dL (0.0012)	admission date (0.0013)		admission date (0.0014)
Indicator of receiving pressors or inotropes within one year before admission date (0.0011)			Count of Serum CO2 test (0.0013)	Automated urinalysis, urine hemoglobin within 8-365 days prior to surgery, mg/dL (0.0011)	Indicator of receiving Antiemetic within one year before admission date (0.0012)		Count of White Blood Cell in blood test (0.0013)
Indicator of receiving Beta-blockers within one year before admission date (0.001)			Count of Serum Sodium test (0.0013)	Indicator of receiving Beta-blockers within one year before admission date (0.0008)	Indicator of receiving Beta-blockers within one year before admission date (0.0012)		Indicator of receiving pressors or inotropes within one year before admission date (0.0012)
Indicator of receiving ACE Inhibitors within one year before admission date (0.0008)			Count of Serum Calcium test (0.0011)	Indicator of receiving Diuretics within one year before admission date (0.0006)	Indicator of receiving statin within one year before admission date (0.0009)		Number of urine glucose tests within 0-7 days prior to surgery (0.0011)
Anesthesia Type (0.0008)			Indicator of receiving Diuretics within one year before admission date (0.0008)	Anesthesia Type (0.0006)	Anesthesia Type (0.0008)		Indicator of receiving ACE Inhibitors within one year before admission date (0.001)
Indicator of receiving Diuretics within one year before admission date (0.0006)			Ethnicity (0.0008)	Indicator of receiving ACE Inhibitors within one year before admission date (0.0006)	Indicator of receiving Diuretics within one year before admission date (0.0008)		Anesthesia Type (0.0008)

© 2022 Ren Y et al. *JAMA Network Open*.

Cardiovascular complication	Prolonged ICU stay	Neurological complication and delirium	Wound complication	Sepsis	Venous thromboembolism	Prolonged mechanical ventilation	Acute kidney injury
**Feature (importance weight)**							
Scheduled room is trauma room or not (0.0006)			Indicator of receiving ACE Inhibitors within one year before admission date (0.0008)	Indicator of receiving statin within one year before admission date (0.0005)	Indicator of receiving ACE Inhibitors within one year before admission date (0.0008)		Indicator of receiving statin within one year before admission date (0.0006)
Indicator of receiving statin within one year before admission date (0.0006)			Indicator of receiving statin within one year before admission date (0.0007)	Ethnicity (0.0003)	Ethnicity (0.0006)		Ethnicity (0.0004)
Indicator of receiving Bicarbonate within one year before admission date (0.0004)			Indicator of receiving Bicarbonate within one year before admission date (0.0003)	Indicator of receiving Bicarbonate within one year before admission date (0.0003)	Scheduled room is trauma room or not (0.0005)		Scheduled room is trauma room or not (0.0003)
Ethnicity (0.0003)			Scheduled room is trauma room or not (0.0003)	Native Language Spoken (0.0002)	Indicator of receiving Bicarbonate within one year before admission date (0.0004)		Indicator of receiving Bicarbonate within one year before admission date (0.0002)
Native Language Spoken (0.0001)			Native Language Spoken (0.0001)	Scheduled room is trauma room or not (0.0002)	Native Language Spoken (0.0004)		Native Language Spoken (0.0001)

Abbreviations: ICU, intensive care unit; ACE, Angiotensin-converting enzyme; CKD, chronic kidney disease.

© 2022 Ren Y et al. *JAMA Network Open*.

eTable 10. Summary of the Contributions of Each Input Feature to Each Outcome for a Random Forest Model With 135 Input Features

Cardiovascular complication	Prolonged ICU stay	Neurological complication and delirium	Wound complication	Sepsis	Venous thromboembolism	Prolonged mechanical ventilation	Acute kidney injury
**Feature (importance weight)**							
Current Procedural Terminology code of the primary procedure (0.1793)	Scheduled post operation location (0.2593)	Current Procedural Terminology code of the primary procedure (0.2093)	Current Procedural Terminology code of the primary procedure (0.2665)	Current Procedural Terminology code of the primary procedure (0.1254)	Current Procedural Terminology code of the primary procedure (0.1586)	Current Procedural Terminology code of the primary procedure (0.2055)	Current Procedural Terminology code of the primary procedure (0.1612)
Scheduled post operation location (0.0659)	Current Procedural Terminology code of the primary procedure (0.2155)	Cerebrovascular Disease (0.0711)	ZIP Code of home address (0.0373)	Number of hemoglobin tests within 7 days prior to surgery (0.0692)	Number of hemoglobin tests within 7 days prior to surgery (0.0536)	Scheduled post operation location (0.142)	Surgery Type (0.0528)
Scheduled surgery room (0.0327)	Surgery Type (0.0635)	Surgery Type (0.0678)	Scheduled surgery room (0.0335)	Min hemoglobin within 7 days prior to surgery, g/dl (0.0459)	Scheduled surgery room (0.0347)	Number of hemoglobin tests within 7 days prior to surgery (0.0454)	Scheduled surgery room (0.0435)
Surgery Type (0.0298)	Scheduled surgery room (0.0467)	Scheduled surgery room (0.061)	Surgery Type (0.0241)	Count of Serum CO2 test (0.0383)	Variance of Hematocrit in blood, % (0.0251)	Surgery Type (0.0374)	Max of Serum creatinine, mg/dL (0.042)
Age (0.0275)	Number of hemoglobin tests within 7 days prior to surgery (0.018)	Scheduled post operation location (0.0538)	Time from Admission to Surgery, days (0.0229)	Count of Urea nitrogen-Creatinine ratio (0.0311)	ZIP Code of home address (0.0244)	Min of Serum Calcium, mmol/L (0.0351)	Min hemoglobin within 7 days prior to surgery, g/dl (0.0319)

© 2022 Ren Y et al. *JAMA Network Open*.

Cardiovascular complication	Prolonged ICU stay	Neurological complication and delirium	Wound complication	Sepsis	Venous thromboembolism	Prolonged mechanical ventilation	Acute kidney injury
**Feature (importance weight)**							
ZIP Code of home address (0.0265)	Medicine or surgery admitting (0.0146)	Number of hemoglobin tests within 7 days prior to surgery (0.02)	Age (0.021)	Min of Hematocrit in blood, % (0.0251)	Count of Hematocrit in blood test (0.0241)	Scheduled surgery room (0.0189)	ZIP Code of home address (0.0287)
Min hemoglobin within 7 days prior to surgery, g/dl (0.0256)	Reference estimated glomerular filtration rate (0.0139)	Age (0.0145)	Distance of residence to hospital, km (0.0205)	Min of Serum Calcium, mmol/L (0.0236)	Body Mass Index (0.0208)	Time from Admission to Surgery, days (0.0172)	Min of Hematocrit in blood, % (0.027)
Body Mass Index (0.0218)	ZIP Code of home address (0.012)	ZIP Code of home address (0.0139)	Prevalence of residents living below poverty at patient residential area, % (0.0188)	Max of White Blood Cell in blood, thou/uL (0.0212)	Surgery Type (0.0207)	Count of Urea nitrogen-Creatinine ratio (0.017)	Age (0.0244)
Time from Admission to Surgery, days (0.0208)	Time from Admission to Surgery, days (0.0119)	Time from Admission to Surgery, days (0.0135)	Reference estimated glomerular filtration rate (0.0169)	Weight Loss (0.0192)	Time from Admission to Surgery, days (0.0202)	Average of Serum Calcium, mmol/L (0.0159)	Reference estimated glomerular filtration rate (0.021)
Reference estimated glomerular filtration rate (0.0208)	Body Mass Index (0.0099)	Count of Urea nitrogen-Creatinine ratio (0.0126)	Min hemoglobin within 8-365 days prior to surgery, g/dl (0.0152)	Fluid and electrolyte disorders (0.0173)	Prevalence of Hispanic residents living below poverty at patient residential area, % (0.0198)	Max of Glucose in blood, mg/dL (0.0158)	Average of Serum creatinine, mg/dL (0.0209)
Distance of residence to	Admission Source (0.0098)	Medicine or surgery	Indicator of receiving pressors or	Scheduled surgery room (0.0169)	Distance of residence to	Weight Loss (0.0127)	Time from Admission to

© 2022 Ren Y et al. *JAMA Network Open*.

Cardiovascular complication	Prolonged ICU stay	Neurological complication and delirium	Wound complication	Sepsis	Venous thromboembolism	Prolonged mechanical ventilation	Acute kidney injury
**Feature (importance weight)**							
hospital, km (0.0194)		admitting (0.0121)	inotropes within one year before admission date (0.0143)		hospital, km (0.0186)		Surgery, days (0.0191)
Fluid and electrolyte disorders (0.0193)	Age (0.0096)	Body Mass Index (0.0117)	Min hemoglobin within 7 days prior to surgery, g/dl (0.0141)	Surgery Type (0.0167)	Prevalence of African American residents living below poverty at patient residential area, % (0.0179)	ZIP Code of home address (0.0111)	Charlson comorbidity index (0.0179)
Min of Hematocrit in blood, % (0.0177)	Distance of residence to hospital, km (0.0094)	Distance of residence to hospital, km (0.0106)	County of home address (0.0136)	Time from Admission to Surgery, days (0.0155)	Average of Red cell distribution width in Blood, % (0.0159)	Body Mass Index (0.0106)	Distance of residence to hospital, km (0.0175)
Weight Loss (0.0141)	Min of Serum Calcium, mmol/L (0.0091)	Reference estimated glomerular filtration rate (0.0095)	Admission Month (0.0132)	Average of White Blood Cell in blood, thou/uL (0.0136)	Reference estimated glomerular filtration rate (0.0159)	Reference estimated glomerular filtration rate (0.0092)	Average of hemoglobin within 8-365 days prior to surgery, g/dl (0.0161)
Charlson comorbidity index (0.0137)	Weight Loss (0.0089)	Prevalence of Hispanic residents living below poverty at patient residential area, % (0.0094)	Average of hemoglobin within 8-365 days prior to surgery, g/dl (0.0127)	Age (0.0132)	Count of White Blood Cell in blood test (0.0147)	Age (0.0092)	Max of Urea nitrogen in blood, mg/dL (0.0154)

© 2022 Ren Y et al. *JAMA Network Open*.

**Cardiovascular complication**	**Prolonged ICU stay**	**Neurological complication and delirium**	**Wound complication**	**Sepsis**	**Venous thromboembolism**	**Prolonged mechanical ventilation**	**Acute kidney injury**
**Feature (importance weight)**							
County of home address (0.0121)	Max of Glucose in blood, mg/dL (0.0085)	Prevalence of African American residents living below poverty at patient residential area, % (0.0089)	Average of hemoglobin within 7 days prior to surgery, g/dl (0.0122)	Body Mass Index (0.0129)	County of home address (0.0128)	Prevalence of Hispanic residents living below poverty at patient residential area, % (0.0092)	Min hemoglobin within 8-365 days prior to surgery, g/dl (0.0136)
Number of hemoglobin tests within 7 days prior to surgery (0.012)	Charlson comorbidity index (0.008)	Median total income at patient residential area, USD (0.0087)	Min of Hematocrit in blood, % (0.0119)	ZIP Code of home address (0.0123)	Max hemoglobin within 8-365 days prior to surgery, g/dl (0.0119)	Average of Glucose in blood, mg/dL (0.0087)	Max hemoglobin within 8-365 days prior to surgery, g/dl (0.0122)
Max hemoglobin within 8-365 days prior to surgery, g/dl (0.0119)	Count of Urea nitrogen-Creatinine ratio (0.0079)	Max of Glucose in blood, mg/dL (0.0084)	Max hemoglobin within 8-365 days prior to surgery, g/dl (0.0111)	Scheduled post operation location (0.0122)	Max of Red cell distribution width in Blood, % (0.0119)	Prevalence of African American residents living below poverty at patient residential area, % (0.0087)	Min of Serum creatinine, mg/dL (0.0121)
Average of hemoglobin within 8-365 days prior to surgery, g/dl (0.0112)	Prevalence of residents living below poverty at patient residential area, % (0.0078)	Min of Serum Calcium, mmol/L (0.0083)	Average of Glucose in blood, mg/dL (0.0108)	Reference estimated glomerular filtration rate (0.0119)	Charlson comorbidity index (0.0118)	Charlson comorbidity index (0.0084)	County of home address (0.0115)

© 2022 Ren Y et al. *JAMA Network Open*.

Cardiovascular complication	Prolonged ICU stay	Neurological complication and delirium	Wound complication	Sepsis	Venous thromboembolism	Prolonged mechanical ventilation	Acute kidney injury
**Feature (importance weight)**							
Min hemoglobin within 8-365 days prior to surgery, g/dl (0.0105)	Prevalence of Hispanic residents living below poverty at patient residential area, % (0.0077)	Count of Potassium in serum test (0.0082)	Average of Hematocrit in blood, % (0.0106)	Prevalence of Hispanic residents living below poverty at patient residential area, % (0.0109)	Scheduled post operation location (0.0118)	Admission Source (0.0083)	Hypertension (0.0113)
Average of Serum Red Blood Cell, Million/uL (0.0105)	Count of Glucose in blood test (0.0075)	Admission Source (0.0081)	Max of Glucose in blood, mg/dL (0.0103)	Emergent or elective admission (0.0106)	Average of hemoglobin within 8-365 days prior to surgery, g/dl (0.0115)	Median total income at patient residential area, USD (0.0082)	Fluid and electrolyte disorders (0.0111)
Min of Serum Calcium, mmol/L (0.0105)	Min hemoglobin within 7 days prior to surgery, g/dl (0.0072)	Average of Serum Calcium, mmol/L (0.0081)	Charlson comorbidity index (0.01)	Distance of residence to hospital, km (0.0104)	Max of Glucose in blood, mg/dL (0.0115)	Distance of residence to hospital, km (0.0081)	Average of hemoglobin within 7 days prior to surgery, g/dl (0.0111)
Max of Urea nitrogen in blood, mg/dL (0.0093)	Average of Serum Calcium, mmol/L (0.0069)	Prevalence of residents living below poverty at patient residential area, % (0.0079)	Average of Red cell distribution width in Blood, % (0.0095)	Prevalence of African American residents living below poverty at patient residential area, % (0.0099)	Average of Glucose in blood, mg/dL (0.0114)	Prevalence of residents living below poverty at patient residential area, % (0.008)	Max of Glucose in blood, mg/dL (0.011)
Max of Glucose in blood, mg/dL (0.0091)	Average of Glucose in blood, mg/dL (0.0067)	Variance of Urea nitrogen-Creatinine ratio (0.0072)	Average of White Blood Cell in blood, thou/uL (0.0094)	Min of White Blood Cell in blood, thou/uL (0.0098)	Min hemoglobin within 8-365 days prior to	Count of Potassium in serum test (0.008)	Average of Hematocrit in blood, % (0.0109)

© 2022 Ren Y et al. *JAMA Network Open*.

Cardiovascular complication	Prolonged ICU stay	Neurological complication and delirium	Wound complication	Sepsis	Venous thromboembolism	Prolonged mechanical ventilation	Acute kidney injury
Feature (importance weight)							
					surgery, g/dl (0.0114)		
Insurance paying the bills (0.0089)	Min of Hematocrit in blood, % (0.0066)	Count of Serum CO2 test (0.007)	No of nephrotoxic drugs received within one year before admission date (0.0092)	Median total income at patient residential area, USD (0.0097)	Number of hemoglobin tests within 8-365 days prior to surgery (0.0109)	Count of Serum CO2 test (0.0077)	Count of Glucose in blood test (0.0105)
Average of Urea nitrogen in blood, mg/dL (0.0089)	Max of White Blood Cell in blood, thou/uL (0.0059)	Charlson comorbidity index (0.0069)	Number of hemoglobin tests within 8-365 days prior to surgery (0.0089)	Prevalence of residents living below poverty at patient residential area, % (0.0095)	Min hemoglobin within 7 days prior to surgery, g/dl (0.0109)	Min of Glucose in blood, mg/dL (0.0076)	Average of Glucose in blood, mg/dL (0.0096)
Average of Serum Calcium, mmol/L (0.0088)	County of home address (0.0058)	Max hemoglobin within 8-365 days prior to surgery, g/dl (0.0069)	Max of White Blood Cell in blood, thou/uL (0.0087)	Charlson comorbidity index (0.0092)	Min of Hematocrit in blood, % (0.0102)	Min hemoglobin within 7 days prior to surgery, g/dl (0.0073)	Min of Glucose in blood, mg/dL (0.009)
Average of Glucose in blood, mg/dL (0.0088)	Average of White Blood Cell in blood, thou/uL (0.0057)	Average of Glucose in blood, mg/dL (0.0068)	Average of The amount of hemoglobin relative to the size of the cell in blood, g/dL (0.0086)	Average of Serum Sodium, mmol/L (0.0088)	Average of The amount of hemoglobin relative to the size of the cell in blood, g/dL (0.0095)	Max hemoglobin within 8-365 days prior to surgery, g/dl (0.007)	Number of hemoglobin tests within 8-365 days prior to surgery (0.0085)
Congestive Heart Failure (0.0087)	Min of Glucose in blood, mg/dL (0.0056)	Max of Urea nitrogen-Creatinine ratio (0.0067)	Average of Potassium in serum, mmol/L (0.0086)	Average of Red cell distribution width in Blood, % (0.0084)	Average of Hematocrit in blood, % (0.0091)	Average of hemoglobin within 8-365 days prior to	Average of Serum Red Blood Cell,

© 2022 Ren Y et al. *JAMA Network Open*.

Cardiovascular complication	Prolonged ICU stay	Neurological complication and delirium	Wound complication	Sepsis	Venous thromboembolism	Prolonged mechanical ventilation	Acute kidney injury
**Feature (importance weight)**							
						surgery, g/dl (0.0064)	Million/uL (0.0083)
Average of Hematocrit in blood, % (0.0086)	Average of hemoglobin within 8-365 days prior to surgery, g/dl (0.0055)	County of home address (0.0067)	Average of Urea nitrogen-Creatinine ratio (0.0083)	Average of hemoglobin within 7 days prior to surgery, g/dl (0.0083)	Average of Urea nitrogen-Creatinine ratio (0.0089)	Medicine or surgery admitting (0.0064)	Average of Red cell distribution width in Blood, % (0.0082)
Min of Glucose in blood, mg/dL (0.0085)	Fluid and electrolyte disorders (0.0052)	Count of Glucose in blood test (0.0066)	Variance of Urea nitrogen-Creatinine ratio (0.0081)	Average of Serum Calcium, mmol/L (0.0083)	Max of Urea nitrogen-Creatinine ratio (0.0086)	Average of Red cell distribution width in Blood, % (0.0063)	Average of Urea nitrogen in blood, mg/dL (0.0081)
Min of platelet in blood, thou/uL (0.0085)	Min of White Blood Cell in blood, thou/uL (0.005)	Weight Loss (0.0064)	Average of Serum Red Blood Cell, Million/uL (0.008)	Average of Hematocrit in blood, % (0.008)	Min of platelet in blood, thou/uL (0.0084)	Min hemoglobin within 8-365 days prior to surgery, g/dl (0.0062)	Min of Serum Calcium, mmol/L (0.0079)
Average of Red cell distribution width in Blood, % (0.0076)	Min hemoglobin within 8-365 days prior to surgery, g/dl (0.0048)	Max of White Blood Cell in blood, thou/uL (0.0064)	Max of Urea nitrogen-Creatinine ratio (0.008)	Max of Glucose in blood, mg/dL (0.0078)	Average of hemoglobin within 7 days prior to surgery, g/dl (0.0083)	County of home address (0.0061)	Average of The amount of hemoglobin relative to the size of the cell in blood, g/dL (0.0077)
Average of hemoglobin within 7 days prior to surgery, g/dl (0.0076)	Max of Urea nitrogen-Creatinine ratio (0.0048)	Min of Glucose in blood, mg/dL (0.0061)	Admission Day (0.0079)	Min of Glucose in blood, mg/dL (0.0077)	Max of platelet in blood, thou/uL (0.0081)	Average of White Blood Cell in blood, thou/uL (0.0059)	Average of platelet in blood, thou/uL (0.0073)

© 2022 Ren Y et al. *JAMA Network Open*.

Cardiovascular complication	Prolonged ICU stay	Neurological complication and delirium	Wound complication	Sepsis	Venous thromboembolism	Prolonged mechanical ventilation	Acute kidney injury
**Feature (importance weight)**							
Average of platelet in blood, thou/uL (0.0076)	Number of hemoglobin tests within 8-365 days prior to surgery (0.0044)	Average of hemoglobin within 8-365 days prior to surgery, g/dl (0.0061)	Max of Red cell distribution width in Blood, % (0.0078)	County of home address (0.0073)	Variance of Urea nitrogen-Creatinine ratio (0.008)	Count of Serum Sodium test (0.0058)	Min of platelet in blood, thou/uL (0.0072)
Max of Urea nitrogen-Creatinine ratio (0.0076)	Average of Urea nitrogen-Creatinine ratio (0.0043)	Admission Month (0.0061)	Max of Serum Red Blood Cell, Million/uL (0.0075)	Max hemoglobin within 8-365 days prior to surgery, g/dl (0.0072)	Average of Serum Red Blood Cell, Million/uL (0.008)	Min of Hematocrit in blood, % (0.0057)	Max of White Blood Cell in blood, thou/uL (0.0071)
Number of hemoglobin tests within 8-365 days prior to surgery (0.0075)	Average of Red cell distribution width in Blood, % (0.0043)	Average of Urea nitrogen-Creatinine ratio (0.006)	Max of platelet in blood, thou/uL (0.0075)	Average of hemoglobin within 8-365 days prior to surgery, g/dl (0.0072)	Max of White Blood Cell in blood, thou/uL (0.0077)	Min of White Blood Cell in blood, thou/uL (0.0055)	Average of Serum Calcium, mmol/L (0.0071)
Max of Serum Red Blood Cell, Million/uL (0.0074)	Average of The amount of hemoglobin relative to the size of the cell in blood, g/dL (0.0043)	Min of Urea nitrogen-Creatinine ratio (0.0059)	Min of platelet in blood, thou/uL (0.0074)	Average of Glucose in blood, mg/dL (0.0072)	Average of White Blood Cell in blood, thou/uL (0.0076)	Max of White Blood Cell in blood, thou/uL (0.0055)	Scheduled post operation location (0.0071)
Average of The amount of hemoglobin relative to the size of the cell in blood, g/dL (0.0072)	Min of Urea nitrogen-Creatinine ratio (0.0043)	Count of Serum Sodium test (0.0059)	Average of platelet in blood, thou/uL (0.0073)	Admission Month (0.007)	Max of The amount of hemoglobin relative to the size of the cell in blood, g/dL (0.0075)	Max of Urea nitrogen-Creatinine ratio (0.0055)	Max of Red cell distribution width in Blood, % (0.007)

© 2022 Ren Y et al. *JAMA Network Open*.

Cardiovascular complication	Prolonged ICU stay	Neurological complication and delirium	Wound complication	Sepsis	Venous thromboembolism	Prolonged mechanical ventilation	Acute kidney injury
**Feature (importance weight)**							
Max of platelet in blood, thou/uL (0.0071)	Average of platelet in blood, thou/uL (0.0041)	Min of Hematocrit in blood, % (0.0057)	Average of chloride in Serum, mmol/L (0.0072)	Max of Red cell distribution width in Blood, % (0.0068)	Max of Serum Red Blood Cell, Million/uL (0.0075)	Admission Month (0.0055)	Average of Potassium in serum, mmol/L (0.007)
Average of Potassium in serum, mmol/L (0.007)	Min of platelet in blood, thou/uL (0.0041)	Average of Red cell distribution width in Blood, % (0.0057)	Max of The amount of hemoglobin relative to the size of the cell in blood, g/dL (0.0071)	Variance of Serum creatinine, mg/dL (0.0068)	Min of White Blood Cell in blood, thou/uL (0.0074)	Average of anion gap in blood, mmol/L (0.0054)	Average of White Blood Cell in blood, thou/uL (0.0068)
Average of Urea nitrogen-Creatinine ratio (0.007)	Average of anion gap in blood, mmol/L (0.004)	Min hemoglobin within 8-365 days prior to surgery, g/dl (0.0056)	Weight Loss (0.0068)	Average of Serum Red Blood Cell, Million/uL (0.0067)	Admission Day (0.0073)	Average of Urea nitrogen-Creatinine ratio (0.0052)	Variance of Urea nitrogen-Creatinine ratio (0.0068)
Min of Urea nitrogen-Creatinine ratio (0.007)	Average of Serum Red Blood Cell, Million/uL (0.0039)	Number of hemoglobin tests within 8-365 days prior to surgery (0.0056)	Medicine or surgery admitting (0.0068)	Count of Serum Sodium test (0.0067)	Average of Serum Calcium, mmol/L (0.0071)	Max of Red cell distribution width in Blood, % (0.0051)	Max of Urea nitrogen-Creatinine ratio (0.0067)
Average of Serum Sodium, mmol/L (0.0069)	Variance of Urea nitrogen-Creatinine ratio (0.0039)	Average of White Blood Cell in blood, thou/uL (0.0056)	Insurance paying the bills (0.0066)	Min hemoglobin within 8-365 days prior to surgery, g/dl (0.0065)	Average of Serum creatinine, mg/dL (0.0068)	Max of Serum creatinine, mg/dL (0.0051)	Count of Urea nitrogen-Creatinine ratio (0.0065)
Max of Serum creatinine, mg/dL (0.0068)	Yes if the admission we happened at night (0.0038)	Variance of Serum creatinine, mg/dL (0.0054)	Average of Serum creatinine, mg/dL (0.0066)	Number of hemoglobin tests within 8-365 days prior	Min of Serum creatinine, mg/dL (0.0068)	Average of The amount of hemoglobin relative to the	Average of anion gap in blood, mmol/L (0.0065)

© 2022 Ren Y et al. *JAMA Network Open*.

**Cardiovascular complication**	**Prolonged ICU stay**	**Neurological complication and delirium**	**Wound complication**	**Sepsis**	**Venous thromboembolism**	**Prolonged mechanical ventilation**	**Acute kidney injury**
**Feature (importance weight)**							
				to surgery (0.0065)		size of the cell in blood, g/dL (0.005)	
Admission Day (0.0066)	Average of Urea nitrogen in blood, mg/dL (0.0036)	Emergent or elective admission (0.0053)	Max hemoglobin within 7 days prior to surgery, g/dl (0.0065)	Variance of Urea nitrogen-Creatinine ratio (0.0063)	Average of Serum Sodium, mmol/L (0.0067)	Min of Urea nitrogen-Creatinine ratio (0.0049)	Max of Serum Red Blood Cell, Million/uL (0.0065)
Max of White Blood Cell in blood, thou/uL (0.0066)	Max of Serum Red Blood Cell, Million/uL (0.0036)	Average of The amount of hemoglobin relative to the size of the cell in blood, g/dL (0.0052)	Average of Serum Calcium, mmol/L (0.0064)	Average of The amount of hemoglobin relative to the size of the cell in blood, g/dL (0.0062)	Max of Serum creatinine, mg/dL (0.0065)	Average of Serum creatinine, mg/dL (0.0048)	Average of Serum Sodium, mmol/L (0.0065)
Max of Red cell distribution width in Blood, % (0.0066)	Max of Red cell distribution width in Blood, % (0.0036)	Min of platelet in blood, thou/uL (0.0052)	Average of Serum CO2, mmol/L (0.0064)	Variance of Hematocrit in blood, % (0.0061)	Average of chloride in Serum, mmol/L (0.0065)	Average of Serum CO2, mmol/L (0.0047)	Admission Day (0.0065)
Average of anion gap in blood, mmol/L (0.0065)	Average of Hematocrit in blood, % (0.0035)	Average of Potassium in serum, mmol/L (0.0051)	Min of Serum creatinine, mg/dL (0.0063)	Count of Hematocrit in blood test (0.0059)	Average of Serum CO2, mmol/L (0.0065)	Number of hemoglobin tests within 8-365 days prior to surgery (0.0047)	Variance of Serum creatinine, mg/dL (0.0064)
Average of Serum creatinine, mg/dL (0.0064)	Max of Serum creatinine, mg/dL (0.0035)	Average of Serum CO2, mmol/L (0.005)	Max of Serum creatinine, mg/dL (0.0062)	Max of Serum Red Blood Cell, Million/uL (0.0057)	Count of Serum Sodium test (0.0064)	Min of Serum CO2 , mmol/L (0.0046)	Min of White Blood Cell in blood, thou/uL (0.0064)
Average of White Blood Cell in blood,	Average of Serum creatinine, mg/dL (0.0035)	Average of platelet in blood, thou/uL (0.0049)	Indicator of receiving Vancomycin within one year	Average of Serum creatinine, mg/dL (0.0056)	Average of Urea nitrogen in blood, mg/dL (0.0061)	Average of Serum Red Blood Cell,	Average of Urea nitrogen-Creatinine ratio (0.0062)

© 2022 Ren Y et al. *JAMA Network Open*.

Cardiovascular complication	Prolonged ICU stay	Neurological complication and delirium	Wound complication	Sepsis	Venous thromboembolism	Prolonged mechanical ventilation	Acute kidney injury
**Feature (importance weight)**							
thou/uL (0.0064)			before admission date (0.0062)			Million/uL (0.0046)	
Average of Serum CO2, mmol/L (0.0063)	Average of Serum Sodium, mmol/L (0.0034)	Min of White Blood Cell in blood, thou/uL (0.0049)	Average of Serum Sodium, mmol/L (0.0059)	Min of Urea nitrogen-Creatinine ratio (0.0055)	Max of Serum Calcium, mmol/L (0.0059)	Max of Serum Calcium, mmol/L (0.0046)	Max of The amount of hemoglobin relative to the size of the cell in blood, g/dL (0.0061)
Variance of Urea nitrogen-Creatinine ratio (0.0063)	Admission Day (0.0034)	Min hemoglobin within 7 days prior to surgery, g/dl (0.0049)	Average of Urea nitrogen in blood, mg/dL (0.0058)	Max of Urea nitrogen-Creatinine ratio (0.0055)	Max hemoglobin within 7 days prior to surgery, g/dl (0.0058)	Min of Serum creatinine, mg/dL (0.0045)	Min of Urea nitrogen-Creatinine ratio (0.0061)
Min of White Blood Cell in blood, thou/uL (0.0062)	Average of Serum CO2, mmol/L (0.0034)	Max of platelet in blood, thou/uL (0.0048)	Min of Serum Calcium, mmol/L (0.0053)	Admission Source (0.0054)	Variance of Red cell distribution width in Blood, % (0.0058)	Average of Urea nitrogen in blood, mg/dL (0.0044)	Insurance paying the bills (0.0061)
Min of Serum creatinine, mg/dL (0.0061)	Average of hemoglobin within 7 days prior to surgery, g/dl (0.0033)	Average of Serum Red Blood Cell, Million/uL (0.0048)	Max of Serum Calcium, mmol/L (0.0051)	Average of Urea nitrogen-Creatinine ratio (0.0053)	Emergent or elective admission (0.0056)	Average of Potassium in serum, mmol/L (0.0043)	Average of Serum CO2, mmol/L (0.0057)
Max of The amount of hemoglobin relative to the size of the cell in blood, g/dL (0.006)	Min of Serum creatinine, mg/dL (0.0032)	Max of Red cell distribution width in Blood, % (0.0047)	Smoking Status (0.005)	Average of Urea nitrogen in blood, mg/dL (0.0053)	Min of Serum Calcium, mmol/L (0.0056)	Average of Hematocrit in blood, % (0.0043)	Number of hemoglobin tests within 7 days prior to surgery (0.0054)

© 2022 Ren Y et al. *JAMA Network Open*.

**Cardiovascular complication**	**Prolonged ICU stay**	**Neurological complication and delirium**	**Wound complication**	**Sepsis**	**Venous thromboembolism**	**Prolonged mechanical ventilation**	**Acute kidney injury**
**Feature (importance weight)**							
Count of Urea nitrogen-Creatinine ratio (0.0057)	Max of Serum Calcium, mmol/L (0.003)	Average of anion gap in blood, mmol/L (0.0046)	Max of Urea nitrogen in blood, mg/dL (0.0047)	Min of Serum creatinine, mg/dL (0.0052)	Variance of platelet in blood, thou/uL (0.0055)	Min of platelet in blood, thou/uL (0.0043)	Congestive Heart Failure (0.005)
Max of chloride in Serum, mmol/L (0.0056)	Count of Potassium in serum test (0.003)	Max of Serum Red Blood Cell, Million/uL (0.0044)	Min of Urea nitrogen in blood, mg/dL (0.0047)	Max of The amount of hemoglobin relative to the size of the cell in blood, g/dL (0.0052)	Count of Urea nitrogen-Creatinine ratio (0.0055)	Max of The amount of hemoglobin relative to the size of the cell in blood, g/dL (0.0042)	Max hemoglobin within 7 days prior to surgery, g/dl (0.0049)
Min of Serum CO2 , mmol/L (0.0053)	Max of chloride in Serum, mmol/L (0.0029)	Average of chloride in Serum, mmol/L (0.0044)	Emergent or elective admission (0.0046)	Max of Serum creatinine, mg/dL (0.0052)	Count of Glucose in blood test (0.0054)	Max of Serum Red Blood Cell, Million/uL (0.0042)	Max of chloride in Serum, mmol/L (0.0046)
Min of Urea nitrogen in blood, mg/dL (0.0052)	Max of Urea nitrogen in blood, mg/dL (0.0028)	Min of Serum CO2 , mmol/L (0.0044)	Fluid and electrolyte disorders (0.0044)	Average of chloride in Serum, mmol/L (0.0052)	Variance of hemoglobin within 7 days prior to surgery, g/dl (0.0054)	Max of platelet in blood, thou/uL (0.0041)	Smoking Status (0.0045)
Max of Serum Calcium, mmol/L (0.0051)	Min of Urea nitrogen in blood, mg/dL (0.0027)	Average of Hematocrit in blood, % (0.0043)	Variance of Hematocrit in blood, % (0.0043)	Average of Potassium in serum, mmol/L (0.0051)	Coagulopathy (0.0051)	Average of platelet in blood, thou/uL (0.0041)	Max of Serum Calcium, mmol/L (0.0042)
Max hemoglobin within 7 days prior to surgery, g/dl (0.005)	Max hemoglobin within 7 days prior to surgery, g/dl (0.0027)	Max of The amount of hemoglobin relative to the size of the cell in blood, g/dL (0.0042)	Min of Serum CO2 , mmol/L (0.0042)	Min of platelet in blood, thou/uL (0.0051)	Variance of White Blood Cell in blood, thou/uL (0.005)	Average of chloride in Serum, mmol/L (0.0041)	Automated urinalysis, urine protein presence within 365 days prior to surgery, mg/dL (0.0041)

© 2022 Ren Y et al. *JAMA Network Open*.

Cardiovascular complication	Prolonged ICU stay	Neurological complication and delirium	Wound complication	Sepsis	Venous thromboembolism	Prolonged mechanical ventilation	Acute kidney injury
**Feature (importance weight)**							
Smoking Status (0.0043)	Min of Serum CO2 , mmol/L (0.0025)	Variance of Hematocrit in blood, % (0.0042)	Variance of Serum CO2, mmol/L (0.004)	Max of platelet in blood, thou/uL (0.0051)	Min of Urea nitrogen in blood, mg/dL (0.005)	Fluid and electrolyte disorders (0.004)	Min of Urea nitrogen in blood, mg/dL (0.0039)
Count of Glucose in blood test (0.0042)	Variance of Hematocrit in blood, % (0.0025)	Average of Serum creatinine, mg/dL (0.0041)	Max of Serum Sodium, mmol/L (0.0039)	Average of Serum CO2, mmol/L (0.005)	Variance of Serum creatinine, mg/dL (0.0048)	Average of Serum Sodium, mmol/L (0.004)	Min of Serum CO2 , mmol/L (0.0038)
Variance of Serum creatinine, mg/dL (0.0034)	Count of Serum CO2 test (0.0024)	Average of Serum Sodium, mmol/L (0.0041)	Count of Urea nitrogen-Creatinine ratio (0.0038)	Average of platelet in blood, thou/uL (0.005)	Max of Serum Sodium, mmol/L (0.0046)	Max of chloride in Serum, mmol/L (0.0039)	Variance of chloride in Serum, mmol/L (0.0032)
Variance of Hematocrit in blood, % (0.0034)	Congestive Heart Failure (0.0023)	Min of Serum creatinine, mg/dL (0.0041)	Count of Glucose in blood test (0.0038)	Average of anion gap in blood, mmol/L (0.0049)	Max of chloride in Serum, mmol/L (0.0046)	Variance of Hematocrit in blood, % (0.0038)	Variance of platelet in blood, thou/uL (0.0031)
Variance of Serum CO2, mmol/L (0.0033)	Max of Serum Sodium, mmol/L (0.0022)	Max of Serum creatinine, mg/dL (0.004)	Variance of White Blood Cell in blood, thou/uL (0.0036)	Max of Urea nitrogen in blood, mg/dL (0.0048)	Count of Potassium in serum test (0.0045)	Average of hemoglobin within 7 days prior to surgery, g/dl (0.0038)	Variance of Serum CO2, mmol/L (0.0031)
Variance of hemoglobin within 7 days prior to surgery, g/dl (0.0033)	Hypertension (0.0022)	Average of hemoglobin within 7 days prior to surgery, g/dl (0.0039)	Variance of platelet in blood, thou/uL (0.0036)	Count of chloride in Serum test (0.0048)	Max of Urea nitrogen in blood, mg/dL (0.0045)	Min of Urea nitrogen in blood, mg/dL (0.0038)	Variance of hemoglobin within 7 days prior to surgery, g/dl (0.0031)
Automated urinalysis, urine protein presence within 365 days prior to surgery, mg/dL (0.0031)	Variance of Serum creatinine, mg/dL (0.0022)	Average of Urea nitrogen in blood, mg/dL (0.0039)	Automated urinalysis, urine protein presence within 365 days prior to surgery, mg/dL (0.0035)	Max of Serum Calcium, mmol/L (0.0045)	Medicine or surgery admitting (0.0045)	Max of Serum Sodium, mmol/L (0.0038)	Marital Status (0.003)

© 2022 Ren Y et al. *JAMA Network Open*.

Cardiovascular complication	Prolonged ICU stay	Neurological complication and delirium	Wound complication	Sepsis	Venous thromboembolism	Prolonged mechanical ventilation	Acute kidney injury
**Feature (importance weight)**							
Marital Status (0.0031)	Insurance paying the bills (0.0021)	Insurance paying the bills (0.0038)	Marital Status (0.0033)	Min of Serum CO2 , mmol/L (0.0045)	Count of Serum CO2 test (0.0043)	Variance of Urea nitrogen-Creatinine ratio (0.0034)	Variance of White Blood Cell in blood, thou/uL (0.0029)
Peripheral Vascular Disease (0.0029)	Automated urinalysis, urine protein presence within 365 days prior to surgery, mg/dL (0.002)	Admission Day (0.0038)	Indicator of receiving Antiemetic within one year before admission date (0.0033)	Max hemoglobin within 7 days prior to surgery, g/dl (0.0045)	Variance of Serum CO2, mmol/L (0.0043)	Max hemoglobin within 7 days prior to surgery, g/dl (0.0034)	Variance of Hematocrit in blood, % (0.0029)
Variance of Urea nitrogen in blood, mg/dL (0.0029)	Variance of hemoglobin within 7 days prior to surgery, g/dl (0.0019)	Max of Serum Calcium, mmol/L (0.0037)	Obesity (0.0033)	Max of Serum Sodium, mmol/L (0.0044)	Min of Serum CO2 , mmol/L (0.0043)	Max of Urea nitrogen in blood, mg/dL (0.0034)	No of nephrotoxic drugs received within one year before admission date (0.0029)
Variance of chloride in Serum, mmol/L (0.0029)	Smoking Status (0.0019)	Max of chloride in Serum, mmol/L (0.0037)	Number of urine hemoglobin tests within 8-365 days prior to surgery (0.0032)	Medicine or surgery admitting (0.0043)	Chronic anemia (0.0042)	Variance of hemoglobin within 7 days prior to surgery, g/dl (0.0034)	Variance of Red cell distribution width in Blood, % (0.0028)
Medicine or surgery admitting (0.0029)	Count of Serum Sodium test (0.0018)	Max hemoglobin within 7 days prior to surgery, g/dl (0.0034)	Variance of Serum creatinine, mg/dL (0.003)	Admission Day (0.0042)	Variance of Urea nitrogen in blood, mg/dL (0.004)	Admission Day (0.0034)	Weight Loss (0.0028)

© 2022 Ren Y et al. *JAMA Network Open*.

Cardiovascular complication	Prolonged ICU stay	Neurological complication and delirium	Wound complication	Sepsis	Venous thromboembolism	Prolonged mechanical ventilation	Acute kidney injury
**Feature (importance weight)**							
Variance of White Blood Cell in blood, thou/uL (0.0028)	Peripheral Vascular Disease (0.0018)	Max of Urea nitrogen in blood, mg/dL (0.0033)	Variance of Urea nitrogen in blood, mg/dL (0.003)	Count of Glucose in blood test (0.0039)	Automated urinalysis, urine protein presence within 365 days prior to surgery, mg/dL (0.0038)	Cerebrovascular Disease (0.0033)	Medicine or surgery admitting (0.0027)
Yes if the admission we happened at night (0.0028)	Variance of White Blood Cell in blood, thou/uL (0.0017)	Max of Serum Sodium, mmol/L (0.003)	Yes if the admission we happened at night (0.0029)	Automated urinalysis, urine protein presence within 365 days prior to surgery, mg/dL (0.0037)	Variance of chloride in Serum, mmol/L (0.0038)	Count of Glucose in blood test (0.0031)	Variance of Serum Calcium, mmol/L (0.0027)
Variance of Red cell distribution width in Blood, % (0.0028)	Count of Hematocrit in blood test (0.0017)	Min of Urea nitrogen in blood, mg/dL (0.003)	Variance of Serum Calcium, mmol/L (0.0028)	Min of Urea nitrogen in blood, mg/dL (0.0036)	Smoking Status (0.0038)	Smoking Status (0.0028)	Peripheral Vascular Disease (0.0026)
No of nephrotoxic drugs received within one year before admission date (0.0027)	No of nephrotoxic drugs received within one year before admission date (0.0017)	Max of Serum CO2, mmol/L (0.0028)	Chronic anemia (0.0026)	Variance of chloride in Serum, mmol/L (0.0034)	Variance of Serum Calcium, mmol/L (0.0037)	Variance of Red cell distribution width in Blood, % (0.0026)	Obesity (0.0026)
Variance of platelet in blood, thou/uL (0.0027)	Variance of Serum CO2, mmol/L (0.0017)	Variance of hemoglobin within 7 days prior to surgery, g/dl (0.0026)	Automated urinalysis, urine hemoglobin within 7 days prior to surgery, mg/dL (0.0025)	Variance of platelet in blood, thou/uL (0.0034)	Insurance paying the bills (0.0036)	Count of Hematocrit in blood test (0.0025)	Variance of Urea nitrogen in blood, mg/dL (0.0026)

© 2022 Ren Y et al. *JAMA Network Open*.

Cardiovascular complication	Prolonged ICU stay	Neurological complication and delirium	Wound complication	Sepsis	Venous thromboembolism	Prolonged mechanical ventilation	Acute kidney injury
**Feature (importance weight)**							
Count of Potassium in serum test (0.0025)	Variance of chloride in Serum, mmol/L (0.0017)	Variance of Red cell distribution width in Blood, % (0.0026)	Scheduled post operation location (0.0024)	Max of chloride in Serum, mmol/L (0.0034)	Peripheral Vascular Disease (0.0034)	Max of Serum CO2, mmol/L (0.0025)	Count of Serum Sodium test (0.0025)
Variance of Serum Calcium, mmol/L (0.0024)	Variance of platelet in blood, thou/uL (0.0016)	Variance of platelet in blood, thou/uL (0.0024)	Sex (0.0024)	Insurance paying the bills (0.0034)	Weight Loss (0.0032)	Variance of Serum CO2, mmol/L (0.0025)	Count of Potassium in serum test (0.0024)
Sex (0.0024)	Variance of Red cell distribution width in Blood, % (0.0016)	Variance of White Blood Cell in blood, thou/uL (0.0024)	Hypertension (0.0023)	Variance of White Blood Cell in blood, thou/uL (0.0033)	Admission Source (0.0032)	Variance of White Blood Cell in blood, thou/uL (0.0024)	CKD status at admission (0.0023)
Count of Hematocrit in blood test (0.0022)	Variance of Serum Calcium, mmol/L (0.0014)	Fluid and electrolyte disorders (0.0023)	Coagulopathy (0.0022)	Variance of Red cell distribution width in Blood, % (0.0033)	Marital Status (0.0031)	Insurance paying the bills (0.0024)	Sex (0.0022)
Hypertension (0.0021)	Variance of Urea nitrogen in blood, mg/dL (0.0014)	Variance of Serum CO2, mmol/L (0.0023)	Peripheral Vascular Disease (0.0022)	Variance of Serum CO2, mmol/L (0.0033)	Cerebrovascular Disease (0.0031)	Variance of platelet in blood, thou/uL (0.0023)	Chronic anemia (0.0019)
Chronic Pulmonary Disease (0.0021)	Marital Status (0.0013)	Variance of Urea nitrogen in blood, mg/dL (0.0022)	Chronic Pulmonary Disease (0.0021)	Count of Serum creatinine test (0.0033)	Hypertension (0.0028)	Variance of Serum creatinine, mg/dL (0.0023)	Cancer (0.0019)
Depression (0.002)	Cerebrovascular Disease (0.0013)	Smoking Status (0.0021)	Depression (0.0019)	Variance of hemoglobin within 7 days prior to surgery, g/dl (0.0033)	Fluid and electrolyte disorders (0.0027)	Variance of chloride in Serum, mmol/L (0.0021)	Chronic Pulmonary Disease (0.0018)

© 2022 Ren Y et al. *JAMA Network Open*.

Cardiovascular complication	Prolonged ICU stay	Neurological complication and delirium	Wound complication	Sepsis	Venous thromboembolism	Prolonged mechanical ventilation	Acute kidney injury
**Feature (importance weight)**							
Valvular Disease (0.002)	Cancer (0.0012)	Count of Serum creatinine test (0.0021)	Rural or city at patient residential area (0.0019)	Max of Serum CO_2, mmol/L (0.0031)	No of nephrotoxic drugs received within one year before admission date (0.0027)	Automated urinalysis, urine protein presence within 365 days prior to surgery, mg/dL (0.002)	Count of Urea nitrogen in blood test (0.0017)
Number of urine hemoglobin tests within 8-365 days prior to surgery (0.0019)	Sex (0.0011)	Variance of chloride in Serum, mmol/L (0.002)	Cancer (0.0019)	Variance of Urea nitrogen in blood, mg/dL (0.0027)	Number of urine hemoglobin tests within 8-365 days prior to surgery (0.0023)	Marital Status (0.0018)	Count of Serum CO_2 test (0.0016)
Cerebrovascular Disease (0.0019)	Emergent or elective admission (0.0011)	Count of Hematocrit in blood test (0.002)	Indicator of receiving Beta-blockers within one year before admission date (0.0019)	Variance of Serum Calcium, mmol/L (0.0025)	Chronic Pulmonary Disease (0.0021)	Hypertension (0.0018)	Race (0.0015)
Chronic anemia (0.0018)	Chronic Pulmonary Disease (0.0011)	Count of Urea nitrogen in blood test (0.002)	Automated urinalysis, urine hemoglobin within 8-365 days prior to surgery, mg/dL (0.0016)	Count of anion gap in blood test (0.0025)	Sex (0.0018)	Congestive Heart Failure (0.0018)	Diabetes (0.0015)
Diabetes (0.0015)	Indicator of receiving Antiemetic within one year before	Hypertension (0.0019)	Number of hemoglobin tests within 7 days prior to surgery (0.0016)	Smoking Status (0.0023)	Depression (0.0017)	Variance of Serum Calcium, mmol/L (0.0017)	Cerebrovascular Disease (0.0015)

© 2022 Ren Y et al. *JAMA Network Open*.

**Cardiovascular complication**	**Prolonged ICU stay**	**Neurological complication and delirium**	**Wound complication**	**Sepsis**	**Venous thromboembolism**	**Prolonged mechanical ventilation**	**Acute kidney injury**
**Feature (importance weight)**							
	admission date (0.0008)						
Race (0.0014)	Depression (0.0008)	Variance of Serum Calcium, mmol/L (0.0019)	Count of Hematocrit in blood test (0.0016)	Count of Urea nitrogen in blood test (0.0022)	Automated urinalysis, urine hemoglobin within 7 days prior to surgery, mg/dL (0.0017)	Emergent or elective admission (0.0016)	Admission Source (0.0013)
Count of Serum Sodium test (0.0014)	Rural or city at patient residential area (0.0008)	Automated urinalysis, urine protein presence within 365 days prior to surgery, mg/dL (0.0019)	Race (0.0015)	No of nephrotoxic drugs received within one year before admission date (0.002)	Count of Serum creatinine test (0.0016)	Variance of Urea nitrogen in blood, mg/dL (0.0016)	Coagulopathy (0.0013)
Automated urinalysis, urine hemoglobin within 8-365 days prior to surgery, mg/dL (0.0013)	Obesity (0.0008)	Marital Status (0.0018)	Diabetes (0.0014)	CKD status at admission (0.002)	Race (0.0016)	No of nephrotoxic drugs received within one year before admission date (0.0016)	Automated urinalysis, urine hemoglobin within 8-365 days prior to surgery, mg/dL (0.0013)
Count of White Blood Cell in blood test (0.0012)	Count of White Blood Cell in blood test (0.0008)	No of nephrotoxic drugs received within one year before admission date (0.0017)	Count of White Blood Cell in blood test (0.0013)	Count of Potassium in serum test (0.0019)	Rural or city at patient residential area (0.0016)	Count of chloride in Serum test (0.0015)	Number of urine hemoglobin tests within 8-365 days prior to surgery (0.0013)
Emergent or elective admission (0.0011)	Count of Urea nitrogen in blood test (0.0007)	Count of chloride in Serum test (0.0013)	CKD status at admission (0.0012)	Marital Status (0.0018)	CKD status at admission (0.0016)	Count of Serum creatinine test (0.0014)	Count of Serum Calcium test (0.0013)

© 2022 Ren Y et al. *JAMA Network Open*.

Cardiovascular complication	Prolonged ICU stay	Neurological complication and delirium	Wound complication	Sepsis	Venous thromboembolism	Prolonged mechanical ventilation	Acute kidney injury
Feature (importance weight)							
Admission Source (0.0011)	Race (0.0007)	Sex (0.0013)	Anesthesia Type (0.0011)	Sex (0.0015)	Count of Urea nitrogen in blood test (0.0015)	CKD status at admission (0.0014)	Count of White Blood Cell in blood test (0.0013)
Coagulopathy (0.0011)	Automated urinalysis, urine hemoglobin within 7 days prior to surgery, mg/dL (0.0007)	Peripheral Vascular Disease (0.0013)	Cerebrovascular Disease (0.0011)	Hypertension (0.0015)	Cancer (0.0015)	Cancer (0.0014)	Count of Hematocrit in blood test (0.0012)
Count of Serum CO2 test (0.0011)	Valvular Disease (0.0007)	Yes if the admission we happened at night (0.0013)	Alcohol abuse or drug (0.0011)	Chronic anemia (0.0014)	Diabetes (0.0015)	Chronic Pulmonary Disease (0.0013)	Indicator of receiving Antiemetic within one year before admission date (0.0012)
CKD status at admission (0.001)	CKD status at admission (0.0007)	Chronic Pulmonary Disease (0.0013)	Liver Disease (0.0011)	Yes if the admission we happened at night (0.0014)	Automated urinalysis, urine hemoglobin within 8-365 days prior to surgery, mg/dL (0.0014)	Peripheral Vascular Disease (0.0012)	Indicator of receiving Beta-blockers within one year before admission date (0.0012)
Liver Disease (0.001)	Diabetes (0.0006)	Depression (0.0013)	Valvular Disease (0.0011)	Count of White Blood Cell in blood test (0.0014)	Count of anion gap in blood test (0.0012)	Yes if the admission we happened at night (0.0012)	Alcohol abuse or drug (0.0011)
Automated urinalysis, urine hemoglobin within 7 days	Chronic anemia (0.0006)	Congestive Heart Failure (0.0012)	Admission Source (0.001)	Number of urine hemoglobin tests within 8-365 days prior	Count of chloride in Serum test (0.0012)	Count of White Blood Cell in blood test (0.0012)	Count of Serum creatinine test (0.0011)

© 2022 Ren Y et al. *JAMA Network Open*.

**Cardiovascular complication**	**Prolonged ICU stay**	**Neurological complication and delirium**	**Wound complication**	**Sepsis**	**Venous thromboembolism**	**Prolonged mechanical ventilation**	**Acute kidney injury**
**Feature (importance weight)**							
prior to surgery, mg/dL (0.001)				to surgery (0.0013)			
Count of anion gap in blood test (0.0009)	Indicator of receiving pressors or inotropes within one year before admission date (0.0006)	Count of anion gap in blood test (0.0011)	Congestive Heart Failure (0.001)	Congestive Heart Failure (0.0012)	Indicator of receiving Antiemetic within one year before admission date (0.0012)	Count of Urea nitrogen in blood test (0.0011)	Emergent or elective admission (0.0011)
Indicator of receiving pressors or inotropes within one year before admission date (0.0009)	Automated urinalysis, urine hemoglobin within 8-365 days prior to surgery, mg/dL (0.0006)	Indicator of receiving Antiemetic within one year before admission date (0.0011)	Count of Potassium in serum test (0.0009)	Chronic Pulmonary Disease (0.0012)	Alcohol abuse or drug (0.0012)	Valvular Disease (0.001)	Valvular Disease (0.001)
Alcohol abuse or drug (0.0009)	Metastatic Carcinoma (0.0005)	Cancer (0.001)	Count of Urea nitrogen in blood test (0.0009)	Peripheral Vascular Disease (0.0011)	Valvular Disease (0.0011)	Number of urine hemoglobin tests within 8-365 days prior to surgery (0.001)	Indicator of receiving Vancomycin within one year before admission date (0.001)
Count of Urea nitrogen in blood test (0.0009)	Count of chloride in Serum test (0.0005)	Count of White Blood Cell in blood test (0.001)	Indicator of receiving Aspirin within one year before admission date (0.0008)	Depression (0.0011)	Myocardial Infarction (0.001)	Sex (0.0009)	Indicator of receiving pressors or inotropes within one year before admission date (0.001)
Indicator of receiving Beta-blockers within	Coagulopathy (0.0005)	Diabetes (0.001)	Count of Serum CO2 test (0.0008)	Obesity (0.0011)	Congestive Heart Failure (0.001)	Indicator of receiving Antiemetic	Count of anion gap in blood test (0.0009)

© 2022 Ren Y et al. *JAMA Network Open*.

Cardiovascular complication	Prolonged ICU stay	Neurological complication and delirium	Wound complication	Sepsis	Venous thromboembolism	Prolonged mechanical ventilation	Acute kidney injury
**Feature (importance weight)**							
one year before admission date (0.0008)						within one year before admission date (0.0009)	
Metastatic Carcinoma (0.0007)	Count of Serum creatinine test (0.0005)	CKD status at admission (0.0009)	Count of anion gap in blood test (0.0008)	Indicator of receiving Vancomycin within one year before admission date (0.0011)	Indicator of receiving statin within one year before admission date (0.001)	Race (0.0008)	Liver Disease (0.0009)
Indicator of receiving Aspirin within one year before admission date (0.0007)	Indicator of receiving Beta-blockers within one year before admission date (0.0004)	Number of urine hemoglobin tests within 8-365 days prior to surgery (0.0009)	Count of Serum Sodium test (0.0007)	Rural or city at patient residential area (0.0011)	Liver Disease (0.001)	Depression (0.0008)	Indicator of receiving Diuretics within one year before admission date (0.0009)
Anesthesia Type (0.0007)	Count of anion gap in blood test (0.0004)	Rural or city at patient residential area (0.0008)	Count of Serum Calcium test (0.0006)	Cerebrovascular Disease (0.001)	Indicator of receiving pressors or inotropes within one year before admission date (0.0009)	Indicator of receiving pressors or inotropes within one year before admission date (0.0008)	Count of chloride in Serum test (0.0009)
Myocardial Infarction (0.0007)	Alcohol abuse or drug (0.0004)	Indicator of receiving pressors or inotropes within one year before admission date (0.0008)	Myocardial Infarction (0.0006)	Race (0.0009)	Indicator of receiving Beta-blockers within one year before admission date (0.0009)	Obesity (0.0008)	Automated urinalysis, urine hemoglobin within 7 days prior to surgery, mg/dL (0.0007)

© 2022 Ren Y et al. *JAMA Network Open*.

Cardiovascular complication	Prolonged ICU stay	Neurological complication and delirium	Wound complication	Sepsis	Venous thromboembolism	Prolonged mechanical ventilation	Acute kidney injury
**Feature (importance weight)**							
Indicator of receiving ACE Inhibitors within one year before admission date (0.0007)	Indicator of receiving Aspirin within one year before admission date (0.0004)	Obesity (0.0008)	Indicator of receiving Diuretics within one year before admission date (0.0005)	Cancer (0.0009)	Count of Serum Calcium test (0.0008)	Coagulopathy (0.0008)	Indicator of receiving Aspirin within one year before admission date (0.0007)
Count of Serum Calcium test (0.0006)	Count of Serum Calcium test (0.0003)	Valvular Disease (0.0008)	Count of Serum creatinine test (0.0005)	Automated urinalysis, urine hemoglobin within 8-365 days prior to surgery, mg/dL (0.0008)	Indicator of receiving Aspirin within one year before admission date (0.0008)	Alcohol abuse or drug (0.0008)	Indicator of receiving ACE Inhibitors within one year before admission date (0.0007)
Indicator of receiving statin within one year before admission date (0.0005)	Myocardial Infarction (0.0003)	Anesthesia Type (0.0007)	Indicator of receiving ACE Inhibitors within one year before admission date (0.0005)	Indicator of receiving pressors or inotropes within one year before admission date (0.0008)	Anesthesia Type (0.0007)	Rural or city at patient residential area (0.0007)	Myocardial Infarction (0.0006)
Count of chloride in Serum test (0.0005)	Indicator of receiving statin within one year before admission date (0.0003)	Coagulopathy (0.0007)	Ethnicity (0.0005)	Coagulopathy (0.0008)	Indicator of receiving Diuretics within one year before admission date (0.0007)	Automated urinalysis, urine hemoglobin within 7 days prior to surgery, mg/dL (0.0007)	Anesthesia Type (0.0006)
Count of Serum creatinine test (0.0005)	Anesthesia Type (0.0003)	Race (0.0007)	Indicator of receiving statin within one year before admission date (0.0005)	Liver Disease (0.0008)	Indicator of receiving ACE Inhibitors within one year before admission date (0.0007)	Diabetes (0.0007)	Indicator of receiving statin within one year before admission date (0.0005)

© 2022 Ren Y et al. *JAMA Network Open*.

Cardiovascular complication	Prolonged ICU stay	Neurological complication and delirium	Wound complication	Sepsis	Venous thromboembolism	Prolonged mechanical ventilation	Acute kidney injury
**Feature (importance weight)**							
Indicator of receiving Vancomycin within one year before admission date (0.0005)	Indicator of receiving Diuretics within one year before admission date (0.0002)	Automated urinalysis, urine hemoglobin within 7 days prior to surgery, mg/dL (0.0006)	Count of chloride in Serum test (0.0005)	Indicator of receiving Antiemetic within one year before admission date (0.0008)	Indicator of receiving Aminoglycosides within one year before admission date (0.0007)	Count of anion gap in blood test (0.0007)	Indicator of receiving Aminoglycosides within one year before admission date (0.0003)
Indicator of receiving Diuretics within one year before admission date (0.0005)	Indicator of receiving Bicarbonate within one year before admission date (0.0001)	Alcohol abuse or drug (0.0006)	Indicator of receiving Aminoglycosides within one year before admission date (0.0003)	Alcohol abuse or drug (0.0007)	Indicator of receiving Vancomycin within one year before admission date (0.0006)	Automated urinalysis, urine hemoglobin within 8-365 days prior to surgery, mg/dL (0.0007)	Ethnicity (0.0003)
Indicator of receiving Bicarbonate within one year before admission date (0.0003)	Indicator of receiving Aminoglycosides within one year before admission date (0.0001)	Indicator of receiving Aspirin within one year before admission date (0.0006)	Indicator of receiving Bicarbonate within one year before admission date (0.0003)	Count of Serum Calcium test (0.0007)	Scheduled room is trauma room or not (0.0003)	Metastatic Carcinoma (0.0006)	Indicator of receiving Bicarbonate within one year before admission date (0.0002)
Scheduled room is trauma room or not (0.0001)	Scheduled room is trauma room or not (0.0)	Automated urinalysis, urine hemoglobin within 8-365 days prior to surgery, mg/dL (0.0006)	Scheduled room is trauma room or not (0.0001)	Valvular Disease (0.0007)	Indicator of receiving Bicarbonate within one year before admission date (0.0003)	Count of Serum Calcium test (0.0006)	Scheduled room is trauma room or not (0.0)
		Indicator of receiving Beta-blockers within one year before		Diabetes (0.0007)		Indicator of receiving Beta-blockers within one year before	

© 2022 Ren Y et al. *JAMA Network Open*.

Cardiovascular complication	Prolonged ICU stay	Neurological complication and delirium	Wound complication	Sepsis	Venous thromboembolism	Prolonged mechanical ventilation	Acute kidney injury
Feature (importance weight)							
		admission date (0.0006)				admission date (0.0006)	
		Chronic anemia (0.0006)		Anesthesia Type (0.0006)		Myocardial Infarction (0.0006)	
		Liver Disease (0.0005)		Indicator of receiving Aspirin within one year before admission date (0.0006)		Native Language Spoken (0.0006)	
		Myocardial Infarction (0.0005)		Automated urinalysis, urine hemoglobin within 7 days prior to surgery, mg/dL (0.0006)		Chronic anemia (0.0006)	
		Count of Serum Calcium test (0.0005)		Indicator of receiving Beta-blockers within one year before admission date (0.0005)		Liver Disease (0.0006)	
		Metastatic Carcinoma (0.0005)		Metastatic Carcinoma (0.0005)		Indicator of receiving Aspirin within one year before admission date (0.0005)	
		Indicator of receiving Vancomycin within one year		Myocardial Infarction (0.0005)		Anesthesia Type (0.0004)	

© 2022 Ren Y et al. *JAMA Network Open*.

Cardiovascular complication	Prolonged ICU stay	Neurological complication and delirium	Wound complication	Sepsis	Venous thromboembolism	Prolonged mechanical ventilation	Acute kidney injury
Feature (importance weight)							
		before admission date (0.0004)					
		Indicator of receiving Diuretics within one year before admission date (0.0004)		Indicator of receiving Diuretics within one year before admission date (0.0004)		Scheduled room is trauma room or not (0.0004)	
		Indicator of receiving statin within one year before admission date (0.0004)		Indicator of receiving statin within one year before admission date (0.0004)		Indicator of receiving statin within one year before admission date (0.0004)	
		Indicator of receiving Bicarbonate within one year before admission date (0.0003)		Indicator of receiving ACE Inhibitors within one year before admission date (0.0004)		Indicator of receiving ACE Inhibitors within one year before admission date (0.0004)	
		Indicator of receiving ACE Inhibitors within one year before admission date (0.0003)		Ethnicity (0.0003)		Ethnicity (0.0003)	
		Indicator of receiving Aminoglycosides within one year before		Indicator of receiving Bicarbonate within one year before		Indicator of receiving Diuretics within one year before	

© 2022 Ren Y et al. *JAMA Network Open*.

Cardiovascular complication	Prolonged ICU stay	Neurological complication and delirium	Wound complication	Sepsis	Venous thromboembolism	Prolonged mechanical ventilation	Acute kidney injury
**Feature (importance weight)**							
		admission date (0.0002)		admission date (0.0002)		admission date (0.0003)	
		Ethnicity (0.0001)		Indicator of receiving Aminoglycosides within one year before admission date (0.0002)		Indicator of receiving Vancomycin within one year before admission date (0.0002)	
		Scheduled room is trauma room or not (0.0001)		Scheduled room is trauma room or not (0.0002)		Indicator of receiving Bicarbonate within one year before admission date (0.0002)	
		Native Language Spoken (0.0001)		Native Language Spoken (0.0001)		Indicator of receiving Aminoglycosides within one year before admission date (0.0002)	

Abbreviations: ICU, intensive care unit; ACE, Angiotensin-converting enzyme; CKD, chronic kidney disease.

© 2022 Ren Y et al. *JAMA Network Open*.

eTable 11. Comparison of Validation Cohort Model Performance With Respect to AUROC, Net Reclassification Index, Percentage of Event Reclassification, and Percentage of No Event Reclassification

Model Versions	Outcomes	Cardiovascular complication	Prolonged ICU stay	Neurological complication and delirium	Wound complication	Sepsis	Venous thromboembolism	Prolonged mechanical ventilation	Acute kidney injury
GAM V1.55	AUROC (95% CI)	0.8 (0.79, 0.8)	0.86 (0.86, 0.87)	0.85 (0.85, 0.86)	0.77 (0.76, 0.77)	0.84 (0.83, 0.84)	0.8 (0.79, 0.81)	0.89 (0.88, 0.9)	0.8 (0.79, 0.8)
GAM V1.101	AUROC (95% CI)	0.78 (0.77, 0.79)	0.85 (0.84, 0.86)	0.83 (0.82, 0.84)	0.69 (0.68, 0.7)	0.84 (0.83, 0.85)	0.78 (0.77, 0.79)	0.87 (0.86, 0.88)	0.79 (0.78, 0.79)
GAM V1.135	AUROC (95% CI)	0.81 (0.8, 0.82)	0.88 (0.87, 0.88)	0.86 (0.86, 0.87)	0.77 (0.77, 0.78)	0.86 (0.85, 0.86)	0.81 (0.8, 0.83)	0.91 (0.9, 0.91)	0.82 (0.81, 0.83)
RF V2.55	AUROC (95% CI)	0.8 (0.79, 0.81)	0.87 (0.87, 0.88)	0.87 (0.86, 0.87)	0.78 (0.77, 0.79)	0.84 (0.83, 0.85)	0.82 (0.81, 0.83)	0.9 (0.89, 0.91)	0.81 (0.8, 0.81)
RF V2.101	AUROC (95% CI)	0.79 (0.78, 0.8)	0.87 (0.86, 0.87)	0.85 (0.84, 0.86)	0.71 (0.7, 0.72)	0.84 (0.83, 0.85)	0.81 (0.79, 0.82)	0.89 (0.88, 0.9)	0.8 (0.79, 0.81)
RF V2.135	AUROC (95% CI)	0.81 (0.81, 0.82)	0.89 (0.88, 0.89)	0.87 (0.87, 0.88)	0.78 (0.78, 0.79)	0.86 (0.85, 0.87)	0.82 (0.81, 0.83)	0.91 (0.9, 0.91)	0.82 (0.82, 0.83)
GAM_V1.101 vs GAM_V1.55	AUROC p-value	<0.001	<0.001	<0.001	<0.001	<0.001	0.51	<0.001	<0.001
GAM_V1.135 vs GAM_V1.55	AUROC p-value	<0.001	<0.001	<0.001	<0.001	<0.001	<0.001	<0.001	<0.001
GAM_V1.135 vs GAM_V1.101	AUROC p-value	<0.001	<0.001	<0.001	<0.001	<0.001	<0.001	<0.001	<0.001
RF_V2.101 vs RF_V2.55	AUROC p-value	<0.001	<0.001	<0.001	<0.001	<0.001	0.67	0.002	0.06

© 2022 Ren Y et al. *JAMA Network Open*.

Model Versions	Outcomes	Cardiovascular complication	Prolonged ICU stay	Neurological complication and delirium	Wound complication	Sepsis	Venous thromboembolism	Prolonged mechanical ventilation	Acute kidney injury
RF_V2.135 vs RF_V2.55	AUROC p-value	<0.001	<0.001	<0.001	0.04	0.10	<0.001	<0.001	<0.001
RF_V2.135 vs RF_V2.101	AUROC p-value	<0.001	<0.001	<0.001	<0.001	<0.001	<0.001	<0.001	<0.001
RF_V2.55 vs GAM_V1.55	AUROC p-value	<0.001	<0.001	<0.001	<0.001	<0.001	<0.001	<0.001	<0.001
RF_V2.101 vs GAM_V1.101	AUROC p-value	<0.001	<0.001	<0.001	<0.001	<0.001	0.01	<0.001	<0.001
RF_V2.135 vs GAM_V1.135	AUROC p-value	0.19	<0.001	<0.001	<0.001	<0.001	0.09	0.50	0.002
RF_V2.135 vs GAM_V1.101	AUROC p-value	<0.001	<0.001	<0.001	<0.001	<0.001	<0.001	<0.001	<0.001
RF_V2.135 vs GAM_V1.55	AUROC p-value	<0.001	<0.001	<0.001	<0.001	<0.001	<0.001	<0.001	<0.001
RF_V2.101 vs GAM_V1.55	AUROC p-value	0.20	0.07	0.02	<0.001	0.34	0.02	0.85	0.15
GAM_V1.101 vs GAM_V1.55	NRI (95% CI), p-value	-0.023 (-0.037, -0.01), 0.001	-0.027 (-0.04, -0.016), <0.001	-0.05 (-0.065, -0.036), <0.001	-0.103 (-0.117, -0.088), <0.001	-0.015 (-0.037, 0.008), 0.18	-0.002 (-0.019, 0.017), 0.87	-0.055 (-0.08, -0.031), <0.001	-0.007 (-0.021, 0.006), 0.30
GAM_V1.135 vs	NRI (95% CI), p-value	0.021 (0.009,	0.022 (0.011,	0.01 (-0.001, 0.02), 0.06	0.012 (0.002, 0.022), 0.02	0.028 (0.008,	0.027 (0.013,	0.02 (0.001, 0.04), 0.05	0.037 (0.024,

© 2022 Ren Y et al. *JAMA Network Open*.

Model Versions	Outcomes	Cardiovascular complication	Prolonged ICU stay	Neurological complication and delirium	Wound complication	Sepsis	Venous thromboembolism	Prolonged mechanical ventilation	Acute kidney injury
GAM_V1.55		0.033), <0.001	0.031), <0.001			0.046), 0.003	0.043), <0.001		0.048), <0.001
GAM_V1.135 vs GAM_V1.101	NRI (95% CI), p-value	0.044 (0.032, 0.058), <0.001	0.049 (0.039, 0.059), <0.001	0.06 (0.046, 0.074), <0.001	0.115 (0.1, 0.129), <0.001	0.042 (0.023, 0.064), <0.001	0.029 (0.013, 0.046), <0.001	0.074 (0.055, 0.092), <0.001	0.044 (0.032, 0.057), <0.001
RF_V2.101 vs RF_V2.55	NRI (95% CI), p-value	-0.018 (-0.034, -0.004), 0.01	-0.018 (-0.029, -0.007), 0.002	-0.043 (-0.058, -0.029), <0.001	-0.105 (-0.119, -0.092), <0.001	-0.024 (-0.044, -0.003), 0.02	0.01 (-0.008, 0.027), 0.28	-0.031 (-0.056, -0.01), 0.007	-0.005 (-0.019, 0.009), 0.51
RF_V2.135 vs RF_V2.55	NRI (95% CI), p-value	0.015 (0.003, 0.027), 0.01	0.025 (0.015, 0.035), <0.001	0.01 (-0.0, 0.02), 0.05	0.004 (-0.005, 0.014), 0.35	0.0 (-0.018, 0.019), 0.97	0.031 (0.018, 0.045), <0.001	0.017 (0.001, 0.033), 0.05	0.028 (0.017, 0.039), <0.001
RF_V2.135 vs RF_V2.101	NRI (95% CI), p-value	0.033 (0.02, 0.047), <0.001	0.043 (0.033, 0.053), <0.001	0.053 (0.04, 0.068), <0.001	0.11 (0.095, 0.123), <0.001	0.025 (0.007, 0.043), 0.008	0.022 (0.008, 0.036), 0.004	0.048 (0.028, 0.068), <0.001	0.033 (0.021, 0.044), <0.001
RF_V2.55 vs GAM_V1.55	NRI (95% CI), p-value	0.008 (-0.003, 0.018), 0.16	0.021 (0.012, 0.029), <0.001	0.028 (0.018, 0.038), <0.001	0.023 (0.014, 0.033), <0.001	0.035 (0.016, 0.053), <0.001	0.008 (-0.005, 0.022), 0.24	0.024 (0.009, 0.039), 0.003	0.013 (0.002, 0.024), 0.02
RF_V2.101 vs GAM_V1.101	NRI (95% CI), p-value	0.012 (0.001, 0.024), 0.04	0.03 (0.02, 0.041), <0.001	0.035 (0.022, 0.048), <0.001	0.021 (0.009, 0.033), <0.001	0.026 (0.006, 0.045), 0.008	0.02 (0.006, 0.035), 0.01	0.047 (0.029, 0.065), <0.001	0.016 (0.004, 0.028), 0.01
RF_V2.135 vs GAM_V1.135	NRI (95% CI), p-value	0.001 (-0.01, 0.013), 0.81	0.024 (0.016, 0.033), <0.001	0.028 (0.019, 0.039), <0.001	0.016 (0.005, 0.025), 0.002	0.008 (-0.012, 0.027), 0.43	0.013 (-0.001, 0.028), 0.07	0.021 (0.004, 0.038), 0.02	0.004 (-0.008, 0.016), 0.49
RF_V2.135 vs GAM_V1.101	NRI (95% CI), p-value	0.045 (0.032, 0.061), <0.001	0.073 (0.062, 0.085), <0.001	0.088 (0.074, 0.104), <0.001	0.131 (0.115, 0.146), <0.001	0.05 (0.027, 0.073), <0.001	0.041 (0.023, 0.059), <0.001	0.095 (0.074, 0.115), <0.001	0.048 (0.035, 0.063), <0.001
RF_V2.135 vs	NRI (95% CI), p-value	0.022 (0.01, 0.036), <0.001	0.046 (0.036,	0.038 (0.026, 0.051), <0.001	0.028 (0.017,	0.035 (0.013,	0.04 (0.025, 0.055), <0.001	0.04 (0.021, 0.06), <0.001	0.041 (0.027,

© 2022 Ren Y et al. *JAMA Network Open*.

Model Versions	Outcomes	Cardiovascular complication	Prolonged ICU stay	Neurological complication and delirium	Wound complication	Sepsis	Venous thromboembolism	Prolonged mechanical ventilation	Acute kidney injury
GAM_V1.55			0.056), <0.001		0.038), <0.001	0.058), 0.002			0.054), <0.001
RF_V2.101 vs GAM_V1.55	NRI (95% CI), p-value	-0.011 (-0.026, 0.003), 0.16	0.003 (-0.009, 0.015), 0.63	-0.015 (-0.03, -0.0), 0.04	-0.082 (-0.096, -0.068), <0.001	0.011 (-0.011, 0.034), 0.35	0.018 (0.001, 0.036), 0.05	-0.007 (-0.032, 0.015), 0.54	0.008 (-0.006, 0.022), 0.26
GAM_V1.101 vs GAM_V1.55	Event reclassification (95% CI), p-value	1.313 (0.055, 2.626), 0.04	-2.311 (-3.379, -1.402), <0.001	1.541 (0.151, 2.873), 0.02	-8.827 (-10.017, -7.564), <0.001	-1.592 (-3.737, 0.631), 0.14	-7.947 (-9.587, -6.227), <0.001	-2.167 (-4.595, 0.081), 0.07	1.712 (0.449, 3.006), 0.01
GAM_V1.135 vs GAM_V1.55	Event reclassification (95% CI), p-value	5.551 (4.496, 6.632), <0.001	-1.1 (-2.064, -0.279), 0.01	3.911 (3.013, 4.887), <0.001	-4.31 (-5.199, -3.403), <0.001	-0.398 (-2.174, 1.328), 0.66	2.292 (0.882, 3.835), 0.002	-1.445 (-3.339, 0.478), 0.13	2.882 (1.741, 4.011), <0.001
GAM_V1.135 vs GAM_V1.101	Event reclassification (95% CI), p-value	4.238 (3.081, 5.429), <0.001	1.211 (0.311, 2.143), 0.007	2.37 (1.06, 3.719), <0.001	4.517 (3.276, 5.853), <0.001	1.194 (-0.772, 3.419), 0.25	10.239 (8.656, 11.864), <0.001	0.722 (-1.117, 2.514), 0.45	1.17 (0.029, 2.344), 0.06
RF_V2.101 vs RF_V2.55	Event reclassification (95% CI), p-value	-1.258 (-2.64, 0.055), 0.06	2.814 (1.869, 3.793), <0.001	-1.215 (-2.497, 0.059), 0.08	2.093 (0.821, 3.295), <0.001	-1.99 (-3.84, 0.0), 0.05	-3.923 (-5.584, -2.183), <0.001	-3.852 (-6.226, -1.828), <0.001	3.282 (1.914, 4.642), <0.001
RF_V2.135 vs RF_V2.55	Event reclassification (95% CI), p-value	0.355 (-0.673, 1.458), 0.51	2.924 (2.054, 3.759), <0.001	2.815 (1.825, 3.719), <0.001	3.626 (2.848, 4.472), <0.001	0.637 (-1.134, 2.429), 0.48	0.204 (-1.094, 1.454), 0.77	-0.722 (-2.324, 0.87), 0.38	4.309 (3.247, 5.438), <0.001
RF_V2.135 vs RF_V2.101	Event reclassification (95% CI), p-value	1.613 (0.403, 2.945), 0.009	0.11 (-0.813, 0.943), 0.80	4.03 (2.821, 5.3), <0.001	1.533 (0.207, 2.659), 0.01	2.627 (0.88, 4.404), 0.003	4.126 (2.738, 5.448), <0.001	3.13 (1.28, 5.15), 0.001	1.027 (-0.058, 2.083), 0.06
RF_V2.55 vs GAM_V1.55	Event reclassification (95% CI), p-value	-1.066 (-2.003, -0.083), 0.04	-3.694 (-4.421, -2.917), <0.001	1.304 (0.353, 2.209), 0.007	-4.828 (-5.657, -4.016), <0.001	2.866 (0.955, 4.71), 0.002	1.274 (0.0, 2.519), 0.06	-0.482 (-1.973, 0.977), 0.54	2.797 (1.778, 3.808), <0.001
RF_V2.101 vs	Event reclassification	-3.637 (-4.749, -	1.431 (0.657,	-1.452 (-2.626, -0.332), 0.01	6.092 (5.022,	2.468 (0.643,	5.298 (3.885,	-2.167 (-3.953, -0.494), 0.02	4.366 (3.273,

© 2022 Ren Y et al. *JAMA Network Open*.

Model Versions	Outcomes	Cardiovascular complication	Prolonged ICU stay	Neurological complication and delirium	Wound complication	Sepsis	Venous thromboembolism	Prolonged mechanical ventilation	Acute kidney injury
GAM_V1.101	(95% CI), p-value	2.581), <0.001	2.31), <0.001		7.066), <0.001	4.337), 0.009	6.869), <0.001		5.489), <0.001
RF_V2.135 vs GAM_V1.135	Event reclassification (95% CI), p-value	-6.262 (-7.24, -5.281), <0.001	0.33 (-0.357, 1.156), 0.38	0.207 (-0.714, 1.204), 0.67	3.108 (2.184, 3.993), <0.001	3.901 (2.062, 5.67), <0.001	-0.815 (-2.081, 0.587), 0.23	0.241 (-1.472, 1.959), 0.78	4.224 (3.155, 5.319), <0.001
RF_V2.135 vs GAM_V1.101	Event reclassification (95% CI), p-value	-2.024 (-3.31, -0.714), 0.002	1.541 (0.667, 2.529), 0.001	2.578 (1.218, 4.001), <0.001	7.625 (6.3, 8.858), <0.001	5.096 (2.92, 7.276), <0.001	9.424 (7.7, 11.077), <0.001	0.963 (-1.07, 2.946), 0.35	5.394 (4.125, 6.667), <0.001
RF_V2.135 vs GAM_V1.55	Event reclassification (95% CI), p-value	-0.711 (-1.779, 0.502), 0.23	-0.77 (-1.715, 0.079), 0.08	4.119 (3.046, 5.3), <0.001	-1.202 (-2.088, -0.347), 0.008	3.503 (1.348, 5.63), 0.001	1.477 (0.151, 2.917), 0.06	-1.204 (-3.088, 0.633), 0.21	7.106 (5.872, 8.357), <0.001
RF_V2.101 vs GAM_V1.55	Event reclassification (95% CI), p-value	-2.324 (-3.798, -0.924), <0.001	-0.88 (-1.865, 0.047), 0.08	0.089 (-1.329, 1.417), 0.90	-2.735 (-3.987, -1.489), <0.001	0.876 (-1.424, 3.18), 0.43	-2.649 (-4.323, -0.909), 0.003	-4.334 (-6.739, -2.092), <0.001	6.079 (4.77, 7.384), <0.001
GAM_V1.101 vs GAM_V1.55	No-Event reclassification (95% CI), p-value	-3.622 (-4.168, -3.06), <0.001	-0.389 (-0.981, 0.244), 0.22	-6.57 (-7.116, -5.997), <0.001	-1.517 (-2.161, -0.823), <0.001	0.1 (-0.456, 0.609), 0.7	7.791 (7.304, 8.297), <0.001	-3.307 (-3.852, -2.738), <0.001	-2.46 (-2.956, -1.956), <0.001
GAM_V1.135 vs GAM_V1.55	No-Event reclassification (95% CI), p-value	-3.455 (-3.928, -2.984), <0.001	3.282 (2.738, 3.799), <0.001	-2.949 (-3.331, -2.564), <0.001	5.507 (5.014, 5.998), <0.001	3.151 (2.725, 3.583), <0.001	0.423 (-0.089, 0.877), 0.07	3.411 (2.992, 3.841), <0.001	0.784 (0.343, 1.253), <0.001
GAM_V1.135 vs GAM_V1.101	No-Event reclassification (95% CI), p-value	0.166 (-0.371, 0.714), 0.56	3.671 (3.07, 4.248), <0.001	3.621 (3.088, 4.181), <0.001	7.024 (6.357, 7.747), <0.001	3.051 (2.54, 3.556), <0.001	-7.368 (-7.819, -6.918), <0.001	6.718 (6.232, 7.247), <0.001	3.244 (2.75, 3.783), <0.001
RF_V2.101 vs RF_V2.55	No-Event reclassification (95% CI), p-value	-0.574 (-1.174, -0.043), 0.05	-4.594 (-5.146, -4.073), <0.001	-3.081 (-3.596, -2.541), <0.001	-12.64 (-13.341, -11.938), <0.001	-0.428 (-0.924, 0.067), 0.11	4.904 (4.382, 5.476), <0.001	0.746 (0.252, 1.231), 0.003	-3.749 (-4.276, -3.235), <0.001
RF_V2.135 vs RF_V2.55	No-Event reclassification	1.116 (0.667,	-0.395 (-0.844, 0.076), 0.11	-1.765 (-2.156, -1.399), <0.001	-3.189 (-3.598, -	-0.604 (-0.997, -	2.941 (2.523,	2.404 (2.027, 2.789), <0.001	-1.513 (-1.953, -

© 2022 Ren Y et al. *JAMA Network Open*.

Model Versions	Outcomes	Cardiovascular complication	Prolonged ICU stay	Neurological complication and delirium	Wound complication	Sepsis	Venous thromboembolism	Prolonged mechanical ventilation	Acute kidney injury
	(95% CI), p-value	1.61), <0.001			2.74), <0.001	0.119), 0.009	3.388), <0.001		1.034), <0.001
RF_V2.135 vs RF_V2.101	No-Event reclassification (95% CI), p-value	1.69 (1.18, 2.219), <0.001	4.199 (3.665, 4.745), <0.001	1.316 (0.83, 1.785), <0.001	9.451 (8.772, 10.177), <0.001	-0.176 (-0.655, 0.276), 0.46	-1.962 (-2.393, -1.537), <0.001	1.658 (1.288, 2.068), <0.001	2.236 (1.808, 2.715), <0.001
RF_V2.55 vs GAM_V1.55	No-Event reclassification (95% CI), p-value	1.835 (1.385, 2.28), <0.001	5.761 (5.278, 6.202), <0.001	1.448 (1.082, 1.813), <0.001	7.144 (6.668, 7.615), <0.001	0.637 (0.219, 1.11), 0.006	-0.443 (-0.843, -0.01), 0.04	2.841 (2.447, 3.241), <0.001	-1.496 (-1.916, -1.051), <0.001
RF_V2.101 vs GAM_V1.101	No-Event reclassification (95% CI), p-value	4.882 (4.383, 5.339), <0.001	1.556 (0.997, 2.105), <0.001	4.937 (4.5, 5.394), <0.001	-3.978 (-4.528, -3.448), <0.001	0.109 (-0.381, 0.584), 0.65	-3.33 (-3.717, -2.895), <0.001	6.893 (6.463, 7.305), <0.001	-2.785 (-3.226, -2.32), <0.001
RF_V2.135 vs GAM_V1.135	No-Event reclassification (95% CI), p-value	6.406 (5.941, 6.883), <0.001	2.084 (1.596, 2.598), <0.001	2.632 (2.212, 3.048), <0.001	-1.551 (-2.019, -1.098), <0.001	-3.118 (-3.545, -2.642), <0.001	2.076 (1.704, 2.506), <0.001	1.834 (1.4, 2.262), <0.001	-3.794 (-4.32, -3.348), <0.001
RF_V2.135 vs GAM_V1.101	No-Event reclassification (95% CI), p-value	6.573 (5.978, 7.187), <0.001	5.755 (5.15, 6.377), <0.001	6.253 (5.701, 6.838), <0.001	5.473 (4.779, 6.188), <0.001	-0.067 (-0.594, 0.479), 0.81	-5.292 (-5.758, -4.8), <0.001	8.547 (8.04, 9.051), <0.001	-0.549 (-1.045, -0.011), 0.05
RF_V2.135 vs GAM_V1.55	No-Event reclassification (95% CI), p-value	2.951 (2.468, 3.47), <0.001	5.366 (4.789, 5.895), <0.001	-0.317 (-0.788, 0.117), 0.17	3.956 (3.429, 4.504), <0.001	0.033 (-0.462, 0.587), 0.90	2.499 (2.01, 2.991), <0.001	5.245 (4.81, 5.742), <0.001	-3.009 (-3.526, -2.46), <0.001
RF_V2.101 vs GAM_V1.55	No-Event reclassification (95% CI), p-value	1.261 (0.682, 1.788), <0.001	1.167 (0.602, 1.747), <0.001	-1.633 (-2.178, -1.079), <0.001	-5.495 (-6.186, -4.769), <0.001	0.209 (-0.337, 0.753), 0.46	4.461 (3.927, 5.027), <0.001	3.587 (3.054, 4.15), <0.001	-5.245 (-5.772, -4.72), <0.001

Model version key: V1=previous GAM versions of *MySurgeryRisk*, V2=latest Random Forest versions of *MySurgeryRisk*, .55=55 input features, .101=101 input features, .135=135 input features.
Abbreviations: GAM, generalized additive model; RF, random forest; NRI, net reclassification improvement; ICU, intensive care unit; CI, confidence interval; AUROC, area under the receiver operating characteristic curve; NRI, Net Reclassification index.
NRI is reported as summation of NRIe (fraction of event reclassification) and NRIne (fraction of no event reclassification). Reclassifications are reported in percentage.
P values for AUROC comparison was performed using Delong paired method.
For NRI, event reclassification, and no-event reclassification, t-test was performed to produce p-values.
95% Confidence intervals were produced using Bootstrap method for 1000 samples.

© 2022 Ren Y et al. *JAMA Network Open*.

eTable 12. Absolute and Relative Risk Associated With High-Risk and Low-Risk Groups for Postoperative Complications With 95% CIs Calculated by 1000 Bootstrap Samples on the Validation Cohort

Complication	Version	Absolute Risk		Relative Risk
		Low-Risk Group	High-Risk Group	High- vs. Low-Risk
Cardiovascular complication	GAM V1.55	6.63 (6.21, 7.03)	32.74 (31.73, 33.74)	4.93 (4.61, 5.32)
	GAM V1.101	6.63 (6.22, 7.02)	30.64 (29.74, 31.56)	4.62 (4.32, 4.94)
	GAM V1.135	5.5 (5.11, 5.92)	31.94 (30.99, 32.89)	5.8 (5.37, 6.26)
	RF V2.55	6.73 (6.32, 7.12)	33.8 (32.8, 34.8)	5.03 (4.71, 5.39)
	RF V2.101	7.08 (6.66, 7.49)	32.96 (31.99, 34.0)	4.66 (4.37, 4.97)
	RF V2.135	6.55 (6.13, 6.94)	34.8 (33.79, 35.9)	5.32 (4.98, 5.71)
Prolonged ICU stay	GAM V1.55	9.24 (8.72, 9.73)	55.27 (54.27, 56.28)	5.98 (5.68, 6.35)
	GAM V1.101	10.32 (9.8, 10.83)	54.19 (53.1, 55.2)	5.25 (4.99, 5.55)
	GAM V1.135	9.36 (8.88, 9.87)	58.21 (57.2, 59.26)	6.22 (5.9, 6.57)
	RF V2.55	10.18 (9.71, 10.66)	60.18 (59.11, 61.28)	5.91 (5.63, 6.23)
	RF V2.101	9.5 (9.02, 9.99)	56.12 (55.07, 57.09)	5.91 (5.61, 6.23)
	RF V2.135	9.02 (8.54, 9.54)	60.6 (59.59, 61.62)	6.72 (6.36, 7.1)
Neurological complication and delirium	GAM V1.55	4.97 (4.64, 5.29)	38.71 (37.53, 39.93)	7.8 (7.28, 8.41)
	GAM V1.101	5.05 (4.7, 5.4)	33.1 (32.08, 34.18)	6.56 (6.08, 7.1)
	GAM V1.135	4.31 (3.97, 4.63)	36.88 (35.78, 38.04)	8.57 (7.91, 9.35)
	RF V2.55	4.64 (4.34, 4.98)	40.78 (39.63, 42.03)	8.79 (8.16, 9.49)
	RF V2.101	5.05 (4.71, 5.41)	37.05 (35.9, 38.17)	7.34 (6.79, 7.92)
	RF V2.135	4.14 (3.83, 4.45)	39.62 (38.5, 40.8)	9.57 (8.88, 10.43)
Wound complication	GAM V1.55	10.73 (10.2, 11.22)	38.7 (37.61, 39.71)	3.61 (3.42, 3.82)
	GAM V1.101	13.69 (13.13, 14.27)	34.44 (33.47, 35.46)	2.52 (2.39, 2.65)
	GAM V1.135	11.3 (10.76, 11.78)	41.94 (40.76, 43.07)	3.72 (3.53, 3.93)
	RF V2.55	11.22 (10.73, 11.74)	43.38 (42.15, 44.54)	3.86 (3.65, 4.08)
	RF V2.101	12.45 (11.9, 13.05)	33.94 (32.98, 34.91)	2.72 (2.58, 2.88)
	RF V2.135	10.58 (10.11, 11.08)	41.6 (40.52, 42.68)	3.93 (3.73, 4.16)

© 2022 Ren Y et al. *JAMA Network Open*.

Complication	Version	Absolute Risk		Relative Risk
		**Low-Risk Group**	**High-Risk Group**	**High- vs. Low-Risk**
Sepsis	GAM V1.55	2.8 (2.56, 3.04)	22.55 (21.58, 23.56)	8.05 (7.35, 8.92)
	GAM V1.101	3.41 (3.15, 3.7)	27.25 (26.07, 28.49)	7.98 (7.26, 8.74)
	GAM V1.135	2.49 (2.27, 2.75)	23.35 (22.4, 24.37)	9.36 (8.43, 10.4)
	RF V2.55	2.66 (2.44, 2.91)	22.53 (21.63, 23.5)	8.46 (7.68, 9.39)
	RF V2.101	2.96 (2.69, 3.23)	25.37 (24.29, 26.52)	8.59 (7.78, 9.54)
	RF V2.135	2.54 (2.3, 2.79)	24.7 (23.69, 25.83)	9.72 (8.77, 10.78)
Venous thromboembolism	GAM V1.55	1.98 (1.76, 2.2)	13.24 (12.5, 14.0)	6.71 (5.94, 7.57)
	GAM V1.101	2.11 (1.9, 2.33)	13.04 (12.29, 13.76)	6.19 (5.51, 7.03)
	GAM V1.135	1.93 (1.72, 2.14)	14.52 (13.69, 15.31)	7.53 (6.65, 8.57)
	RF V2.55	1.74 (1.54, 1.94)	13.94 (13.19, 14.73)	8.02 (7.07, 9.2)
	RF V2.101	1.9 (1.69, 2.13)	13.45 (12.71, 14.26)	7.07 (6.26, 8.04)
	RF V2.135	1.71 (1.51, 1.92)	13.78 (13.03, 14.58)	8.06 (7.12, 9.26)
Prolonged mechanical ventilation	GAM V1.55	1.21 (1.06, 1.38)	19.1 (18.1, 20.1)	15.83 (13.73, 18.12)
	GAM V1.101	1.43 (1.24, 1.62)	16.58 (15.67, 17.45)	11.62 (10.19, 13.4)
	GAM V1.135	1.26 (1.1, 1.43)	21.72 (20.62, 22.83)	17.21 (15.13, 19.7)
	RF V2.55	1.23 (1.07, 1.4)	21.38 (20.25, 22.49)	17.42 (15.03, 19.96)
	RF V2.101	1.46 (1.29, 1.64)	21.28 (20.18, 22.45)	14.55 (12.79, 16.64)
	RF V2.135	1.25 (1.08, 1.43)	23.73 (22.47, 24.89)	19.03 (16.53, 22.01)
Acute kidney injury	GAM V1.55	7.24 (6.82, 7.7)	34.91 (33.82, 35.99)	4.82 (4.5, 5.16)
	GAM V1.101	7.07 (6.67, 7.5)	33.39 (32.38, 34.46)	4.73 (4.42, 5.07)
	GAM V1.135	6.51 (6.12, 6.94)	36.56 (35.42, 37.71)	5.61 (5.21, 6.02)
	RF V2.55	6.73 (6.33, 7.15)	34.5 (33.46, 35.61)	5.13 (4.79, 5.5)
	RF V2.101	6.26 (5.84, 6.66)	32.61 (31.61, 33.68)	5.22 (4.85, 5.63)
	RF V2.135	5.82 (5.45, 6.24)	34.57 (33.51, 35.68)	5.94 (5.5, 6.41)
30-day mortality	GAM V1.55	0.54 (0.42, 0.67)	5.16 (4.61, 5.76)	9.62 (7.5, 12.41)
	GAM V1.101	0.57 (0.44, 0.7)	4.64 (4.11, 5.17)	8.15 (6.37, 10.8)
	GAM V1.135	0.52 (0.41, 0.64)	5.36 (4.79, 5.97)	10.3 (8.1, 13.32)

© 2022 Ren Y et al. *JAMA Network Open*.

Complication	Version	Absolute Risk		Relative Risk
		Low-Risk Group	High-Risk Group	High- vs. Low-Risk
	RF V2.55	0.44 (0.34, 0.57)	4.89 (4.39, 5.44)	11.04 (8.54, 15.06)
	RF V2.101	0.69 (0.56, 0.82)	6.13 (5.46, 6.86)	8.96 (7.09, 11.37)
	RF V2.135	0.56 (0.44, 0.69)	6.09 (5.46, 6.78)	10.95 (8.71, 14.13)
90-day mortality	GAM V1.55	0.83 (0.7, 1.0)	7.56 (6.88, 8.29)	9.09 (7.32, 11.19)
	GAM V1.101	0.72 (0.58, 0.87)	6.42 (5.83, 6.97)	8.88 (7.3, 11.47)
	GAM V1.135	0.96 (0.81, 1.12)	7.59 (6.86, 8.31)	7.91 (6.64, 9.74)
	RF V2.55	1.01 (0.86, 1.18)	7.99 (7.28, 8.77)	7.92 (6.63, 9.6)
	RF V2.101	0.7 (0.56, 0.85)	6.96 (6.34, 7.58)	9.87 (8.07, 12.52)
	RF V2.135	0.8 (0.67, 0.96)	8.02 (7.32, 8.72)	9.94 (8.2, 12.38)

Model version key: V1=previous GAM versions of *MySurgeryRisk*, V2=latest Random Forest versions of *MySurgeryRisk*, .55=55 input features, .101=101 input features, .135=135 input features.
Abbreviations: GAM, generalized additive model; RF, random forest; ICU, intensive care unit.
Risk values are presented with 95% confidence intervals.

© 2022 Ren Y et al. *JAMA Network Open*.

eReferences

1. Bihorac A, Brennan M, Ozrazgat-Baslanti T, et al. National surgical quality improvement program underestimates the risk associated with mild and moderate postoperative acute kidney injury. *Crit Care Med.* 2013;41(11):2570-2583, PMID: 23928835, PMC3812338.
2. Datta S, Loftus TJ, Ruppert MM, et al. Added Value of Intraoperative Data for Predicting Postoperative Complications: The MySurgeryRisk PostOp Extension. *J Surg Res.* 2020;254:350-363, PMID: 32531520, PMC7755426.
3. United States Census Bureau. Decennial Census of Population and Housing by Decades. Accessed Sep 9, 2021. https://www.census.gov/programs-surveys/decennial-census/decade.2010.html
4. DeNavas-Walt C, Proctor BD, Smith JC. *Income, Poverty, and Health Insurance Coverage in the United States: 2009.* U.S. Government Printing Office, Washington, DC.; 2010.
5. Pebesma EJ, Bivand RS. Classes and methods for spatial data in R. *R News* 2005;5(2). http://cran.r-project.org/doc/Rnews/. .
6. Bihorac A, Ozrazgat-Baslanti T, Mahanna E, et al. Long-Term Outcomes for Different Forms of Stress Cardiomyopathy After Surgical Treatment for Subarachnoid Hemorrhage. *Anesth Analg.* 2016;122(5):1594-1602, PMID: 27007075, PMC4857194.
7. Adhikari L, Ozrazgat-Baslanti T, Ruppert M, et al. Improved predictive models for acute kidney injury with IDEA: Intraoperative Data Embedded Analytics. *PLoS One.* 2019;14(4):e0214904, PMID: 30947282, PMC6448850.
8. Bihorac A, Ozrazgat-Baslanti T, Ebadi A, et al. MySurgeryRisk: Development and Validation of a Machine-learning Risk Algorithm for Major Complications and Death After Surgery. *Ann Surg.* 2018;269(4):652-662, PMID: 29489489, PMC6110979.
9. Kidney Disease: Improving Global Outcomes (KDIGO) Acute Kidney Injury Work Group. KDIGO Clinical Practice Guideline for Acute Kidney Injury. *Kidney inter, Suppl.* 2012;2(1):1-138, PMID: PMC4089619.
10. *VIF Regression: A Fast Regression Algorithm for Large Data* [computer program]. Version 1.0March 2011.
11. Elixhauser A, Steiner C, Harris DR, Coffey RM. Comorbidity Measures for Use with Administrative Data. *Med Care.* 1998;36(1):8-27, PMID.
12. Wald R, Waikar SS, Liangos O, Pereira BJ, Chertow GM, Jaber BL. Acute renal failure after endovascular vs open repair of abdominal aortic aneurysm. *J Vasc Surg.* 2006;43(3):460-466; discussion 466, PMID: 16520155.
13. Charlson ME, Pompei P, Ales KL, MacKenzie CR. A new method of classifying prognostic comorbidity in longitudinal studies: development and validation. *J Chronic Dis.* 1987;40(5):373-383, PMID: 3558716.
14. U.S. Department of Veterans Affairs VHA. National Drug File – Reference Terminology (NDF-RT™) Documentation. 2015; http://evs.nci.nih.gov/ftp1/NDF-RT/NDF-RT%20Documentation.pdf Accessed 05/16/2017.
15. Boulle M. A grouping method for categorical attributes having very large number of values. *Machine Learning and Data Mining in Pattern Recognition, Proceedinds.* 2005;3587:228-242, PMID: ISI:000230895100023.
16. Pyle D. *Data preparation for data mining.* Vol 1: Morgan Kaufmann; 1999.
17. Korenkevych D, Ozrazgat-Baslanti T, Thottakkara P, et al. The Pattern of Longitudinal Change in Serum Creatinine and 90-Day Mortality After Major Surgery. *Ann Surg.* 2016;263(6):1219-1227, PMID: 26181482, PMC4829495.
18. Bishop CM. *Pattern recognition and machine learning.* New York: Springer; 2006.
19. AHRQ. Clinical Classifications Software for Services and Procedures; https://www.hcup-us.ahrq.gov/toolssoftware/ccs_svcsproc/ccssvcproc.jsp. Accessed Sep 9, 2021.

© 2022 Ren Y et al. *JAMA Network Open*.

20. Fluss R, Faraggi D, Reiser B. Estimation of the Youden Index and its associated cutoff point. *Biometrical Journal.* 2005;47(4):458-472, PMID.
21. Pardalos PM, Boginski VL, Vazacopoulos A. *Data mining in biomedicine.* New York, NY: Springer; 2007.
22. Hastie T, Tibshirani R. *Generalized additive models.* 1st ed. London ; New York: Chapman and Hall; 1990.
23. Wood SN. Fast stable restricted maximum likelihood and marginal likelihood estimation of semiparametric generalized linear models. *J R Stat Soc B.* 2011;73:3-36, PMID: ISI:000285970300002.
24. Breiman L. Random Forests. *Machine Learning.* 2001;45(1):5-32, PMID.
25. Breiman L. Bagging predictors. *Machine Learning.* 1996;24(2):123-140, PMID.
26. Ho TK. The random subspace method for constructing decision forests. *IEEE transactions on pattern analysis and machine intelligence.* 1998;20(8):832-844, PMID.
27. Gray KR, Aljabar P, Heckemann RA, Hammers A, Rueckert D, Initiative AsDN. Random forest-based similarity measures for multi-modal classification of Alzheimer's disease. *Neuroimage.* 2013;65:167-175, PMID.
28. Liaw A, Wiener M. Classification and regression by randomForest. *R news.* 2002;2(3):18-22, PMID.
29. Feng Z, Rana Bhat R, Yuan X, et al. Intelligent Perioperative System: Towards Real-time Big Data Analytics in Surgery Risk Assessment. *ArXiv e-prints.* Vol 17092017.

© 2022 Ren Y et al. *JAMA Network Open*.
